# A novel inflammatory response-related signature predicts the prognosis of cutaneous melanoma and the effect of antitumor drugs

**DOI:** 10.1186/s12957-022-02726-8

**Published:** 2022-08-19

**Authors:** Jiahua Xing, Yan Li, Youbai Chen, Yan Han

**Affiliations:** 1 Department of Plastic and Reconstructive Surgery, The First Medical Center, Chinese PLA General Hospital, Beijing, 100853 China; 2 School of Medicine, Nankai University, Tianjin, 300071 China

**Keywords:** Cutaneous melanoma, Inflammatory genes, Prognostic model, Immune states, Tumor environment, Drug sensitivity

## Abstract

Cutaneous melanoma (CM) is a skin cancer that is highly metastatic and aggressive, with a dismal prognosis. This is the first study to use inflammatory response-related genes to build a model and evaluate their predictive significance in CM. This study used public databases to download CM patients’ mRNA expression profiles and clinical data to create multigene prognostic markers in the UCSC cohort. We compared overall survival (OS) between high- and low-risk groups using the Kaplan-Meier curve and determined independent predictors using Cox analysis. We also used enrichment analysis to assess immune cell infiltration fraction and immune pathway-related activity using KEGG enrichment analysis. Furthermore, we detected prognostic genes’ mRNA and protein expression in CM and normal skin tissues using qRT-PCR and immunohistochemistry. Finally, we developed a 5-gene predictive model that showed that patients in the high-risk group had a considerably shorter OS than those in the low-risk group. The analysis of the receiver operating characteristic (ROC) curve proved the model’s predictive ability. We also conducted a drug sensitivity analysis and discovered that the expression levels of prognostic genes were substantially linked with cancer cell sensitivity to antitumor medicines. The findings show that the model we developed, which consists of five inflammatory response-related genes, can be used to forecast the prognosis and immunological state of CM, giving personalized and precision medicine a new goal and direction.

**Supplementary Information:**

The online version contains supplementary material available at 10.1186/s12957-022-02726-8.

## Introduction

Melanoma is a highly aggressive and invasive tumor that arises from melanocytes, often in the skin and mucous membranes [1]. According to the American Joint Committee on Cancer (AJCC), between 2018 and 2021, there will be 91,270 and 106,110 new melanoma cases, respectively [2, 3]. According to statistics, the global incidence of melanoma was around 3.9/100,000 in 2017, representing a 41.2% increase since 1990 [4]. Despite accounting for a small percentage of skin cancers, cutaneous melanoma (CM) has a significant recurrence rate, mortality, and medication resistance compared to other skin malignancies [5–7]. After complete resection, early-stage CM has a high cure rate, with a mortality rate of 70% and a 5-year survival rate of fewer than 16% in stages 3/4 patients [8]. As a result, novel CM biomarkers must be investigated to guide clinical treatment and enhance the prognosis of CM patients. Gene-based biomarkers have gotten much attention since human gene sequencing technologies developed [9].

Researchers have been studying the role of inflammation in the genesis and progression of cancer [10–12]. Inflammation can both promote and prevent cancer [13, 14]. The link between cancer and indicators of the inflammatory response can be investigated by evaluating basic blood measurements. Many inflammatory response-related characteristics in the peripheral blood of patients with CM, such as thrombocytosis, leukocytosis, hypoproteinemia, and increased plasma fibrin, have been established in investigations [15, 16]. In the overall survival analysis of cancer, clinical systemic inflammatory markers such as neutrophil ratio, platelet-lymphocyte ratio, and lymphocyte-monocyte ratio revealed substantial predictive potential independent of previously identified prognostic variables for CM [17]. The link between inflammatory response-related genes and CM prognosis, on the other hand, is uncertain [18].

In this study, we utilized the UCSC database to download the mRNA expression profiles of CM patients as well as the corresponding clinical data, used differential analysis to find the inflammatory response-related genes differentially expressed in CM, built a CM prognostic marker model, and validated the model’s stability and reliability using training and validation sets. We used functional enrichment analysis to investigate the model’s potential mechanisms of action and looked at the association between predictive gene expression and the type of immune infiltration. In addition, we looked at the relationship between prognostic gene expression and tumor chemoresistance, and our findings were then confirmed using qRT-PCR and IHC (data from the Human Protein Atlas), resulting in novel concepts for predicting CM prognosis.

## Material and methods

### Data download

We used the UCSC database (https://xena.ucsc.edu/) to find the GDC TCGA melanoma dataset and downloaded the HTSeq-fpkm dataset (*n* = 472) as the tumor group. As the control group (*n* = 323), we looked for the GTEX dataset on UCSC, retrieved the TOIL RSEM fpkm dataset, and extracted the skin data from it. We next joined the tumor and control groups to create our gene expression matrix. In addition, we obtained data on inflammation-related genes (*n* = 200) from the GSEA database (http://www.gsea-msigdb.org/). For the following study analysis, we shall merge the above datasets.

### Screening for differential genes (DEGs) related to inflammatory response

We used the limma package in R language to identify differential genes (DEGs) associated with inflammatory responses, and we set the value of corFilter ≥ 0.4 and pvaluefilter ≤ 0.01. In the search for differential genes (DEGs), we set the significance threshold to |log_2_FC| ≥ 2 and the false-positive rate (FDR) ≤ 0.05.

### Modeling of inflammatory response-related prognosis in the training and validation groups

Overall survival (OS) was used as a clinical endpoint in our study, and to improve the model’s accuracy, we screened patients with prognostic information in the tumor group patient group and then divided them into a training group (*n* = 228) and a validation group (*n* = 226). We then used one-way Cox analysis to screen for overall survival (OS) associated with inflammatory differential genes (*n* = 15) and then adjusted *P*-values using the Benjamini and Hochberg (BH) method. Hazard ratios (HR) were utilized to assess if an inflammatory factor was a risk factor (*HR* > 1) or a protective factor (*HR* < 1).

We used the least absolute shrinkage and selection operator (LASSO) Cox regression analysis to develop prognostic models for fitting overall survival (OS) in CM patients based on these inflammatory differential genes. We used the R language’s glmnet package to select and shrink variables such that some of the regression coefficients were strictly equal to 0 to obtain the best prognostic model [19]. We used the normalized expression matrix of potential prognostic factors identified by DEGs as the independent variable and the overall survival (OS) and status of patients as the dependent variable in the regression analysis. We used tenfold cross-validation to determine the prediction model’s penalty parameter (λ) and followed the minimum criterion (the value of *λ* corresponds to the lowest likelihood deviation). We produced patient risk scores and risk profile models based on the expression levels of important inflammatory response genes and their accompanying regression coefficients. The risk score is calculated as follows: *β* (hub gene_1_) × expression (hub gene_1_) + *β* (hub gene_2_) × expression (hub gene_2_) + *β* (hub gene_n_) × expression (hub gene_n_)

### Model evaluation

We classified patients in the training and validation groups into low- and high-risk groups using the risk score formula and the median score value of the risk score in the training group as the cutoff point. We then used the R package survminer to plot Kaplan-Meier survival curves for overall survival (OS) in low- and high-risk groups, as well as ROC (receiver operating characteristic) curves to assess the model’s prediction usefulness and accuracy. We used the rtsne and ggplot2 packages in R to perform principal component analysis (PCA) [20] and t-SNE [21] analysis on the gene expression levels in the built models to minimize the dimensionality and analyze the distribution of the low- and high-risk groups.

### Independent prognostic analysis

We employed univariate and multivariate Cox regression to determine if our prognostic model was independent of other commonly used clinical variables (e.g., age, gender) associated with OS in CM patients. Independent prognosis analyses were performed on all sample groups, the training group, and the validation group (*P* < 0.05). To predict the chance of survival in CM patients, we utilized a multifactor logistic model that included a risk score model and traditional clinical data (including age and gender). We used the model and examined its accuracy in the training and validation groups.

### Functional enrichment: GSEA analysis

Gene set enrichment analysis (GSEA) ranks genes based on their differential expression in two types of samples and then determines whether the preset gene set is enriched at the top or bottom of this ranking table. We analyzed inflammatory response-related prognostic genes across high- and low-risk groups using GSEA analysis (version 4.2.0) to identify differential KEGG signaling networks and putative biological processes influencing tumor growth [22]. We set the number of permutations to 1000 and the type of permutation to phenotypic, with a significance threshold of *P* < 0.05. The immune infiltration status of patients with CM and the immunological differences between high- and low-risk groups were determined using single-sample gene set enrichment analysis (ssGSEA). We calculated the infiltration fraction of 16 immune cells and the activation of 13 immune-related pathways in the high-risk and low-risk groups using the R language package GSVA.

### Tumor microenvironment (TME) and immune scoring

Malignant tissues include not just tumor cells but also normal epithelial and stromal cells, immune cells, and vascular cells associated with tumors. Infiltrating stromal and immune cells, on the other hand, constitute the majority of normal cells in tumor tissue and play a critical role in tumor biology. We typically utilize immune, stem cell, and stromal scores to quantify the degree of immune and mesenchymal stromal cell infiltration in various tumor tissues. Spearman correlation analysis determined the link between risk and various scores. To examine the link between risk score and immune infiltration subtypes, we utilized a two-way analysis of variance (2-way ANOVA). Tumor stem cell characteristics are frequently employed to quantify the stem cell-like properties of tumors, and the link between tumor stemness and risk score was analyzed using Spearman correlation. The ESTIMATE algorithm in the R language ESTIMATE package was used to estimate the rate of the immune stromal component in TME for each sample, which is reported as three scores: stemnessScore, immuneScore, and stromalScore. Additionally, we employed computational approaches such as ESTIMATE [23], TIMER [24], MCP-counter [25], CIBERSORTx [26], and ssGSEA in the training group to synthesize immunological differences in the risk model using the R language’s pheatmap package to visualize the graphs.

### Drug sensitivity analysis

To study the association between inflammation-related prognostic gene expression and drug sensitivity, we used the CellMiner database (https://discover.nci.nih.gov/cellminer/home.do) for drug sensitivity prediction and Pearson correlation analysis. After selecting the processed data set and downloading the RNA expression data and drug data (compound activity: DTP NCI-60), we read the drug-related data and screened the drug criteria, and after completing the preparation of the gene expression data, we completed the drug sensitivity analysis using the R packages impute and limma. Finally, we visualized and completed the scatter plot using the R packages ggplot2 and ggpubr.

### Validation of key genes in CM and paraneoplastic tissues

Following permission from the Chinese PLA General Hospital’s Human Research Ethics Committee, we collected eight pairs of CM and para-cancerous normal tissue specimens. All patients were informed and signed an informed consent form. The relative expression of five key genes was determined using qRT-PCR. We isolated total RNA from cancer and paraneoplastic tissues using TRIzol and determined the quantity of RNA using a NanoDrop spectrophotometer. We then reverse-transcribed the RNA into cDNA using a 5 × RT Master Mix (BioRad) and analyzed mRNA expression levels using a 2 × SYBR Green PCR Kit. The expression of each gene was standardized to GAPDH. Using the 2^−∆∆Ct^ technique, we quantified the real-time PCR analysis and determined the relative expression of genes linked with the validation reaction. The following primer sequences were used: C3AR1: forward, AAG CCA ATC TGG TGT CAG AAT C; reverse, CAG GAA TGC ACA TCA CAA AAG C; CXCL10: forward, GTG GCA TTC AAG GAG TAC CTC; reverse, TGA TGG CCT TCG ATT CTG GAT T; EIF2AK2: forward, GCC GCT AAA CTT GCA TAT CTT CA; reverse, TCA CAC GTA GTA GCA AAA GAA CC; EMP3: forward, CCT GAA TCT CTG GTA CGA CTG C; reverse, GCC ATT CTC GCT GAC ATT ACT G; ICAM1: forward, ATG CCC AGA CAT CTG TGT CC; and reverse, GGG GTC TCT ATG CCC AAC AA. The Huada Corporation synthesized all our primers (Beijing, China). We next used the online database HPA (https://www.proteinatlas.org/) to confirm the differential expression of immunohistochemical proteins associated with important genes in normal tissues and CM.

### Statistical analysis

The Wilcoxon test was used to compare DEGs in CM and para-cancerous normal tissue, and the chi-square test was used to compare various proportions. The Mann-Whitney test was used to compare the ssGSEA scores of immune cells or immunological pathways across high- and low-risk groups, and the *P*-values were adjusted using the BH technique. We analyzed variations in OS between groups using Kaplan-Meier analysis and screened for independent determinants of OS using univariate and multivariate Cox analysis. The correlation study of prognostic model risk scores or prognostic gene expression levels with stemnessScore, immuneScore, stromalScore, and drug sensitivity was performed using Spearman and Pearson correlation analysis. All statistical analyses were conducted using the R programming language (version 4.1.1). All statistical tests were two sided, and a significance level of *P* < 0.05 was considered statistically significant.

## Result

### Acquisition of genes related to inflammatory response

**Fig. 1:**
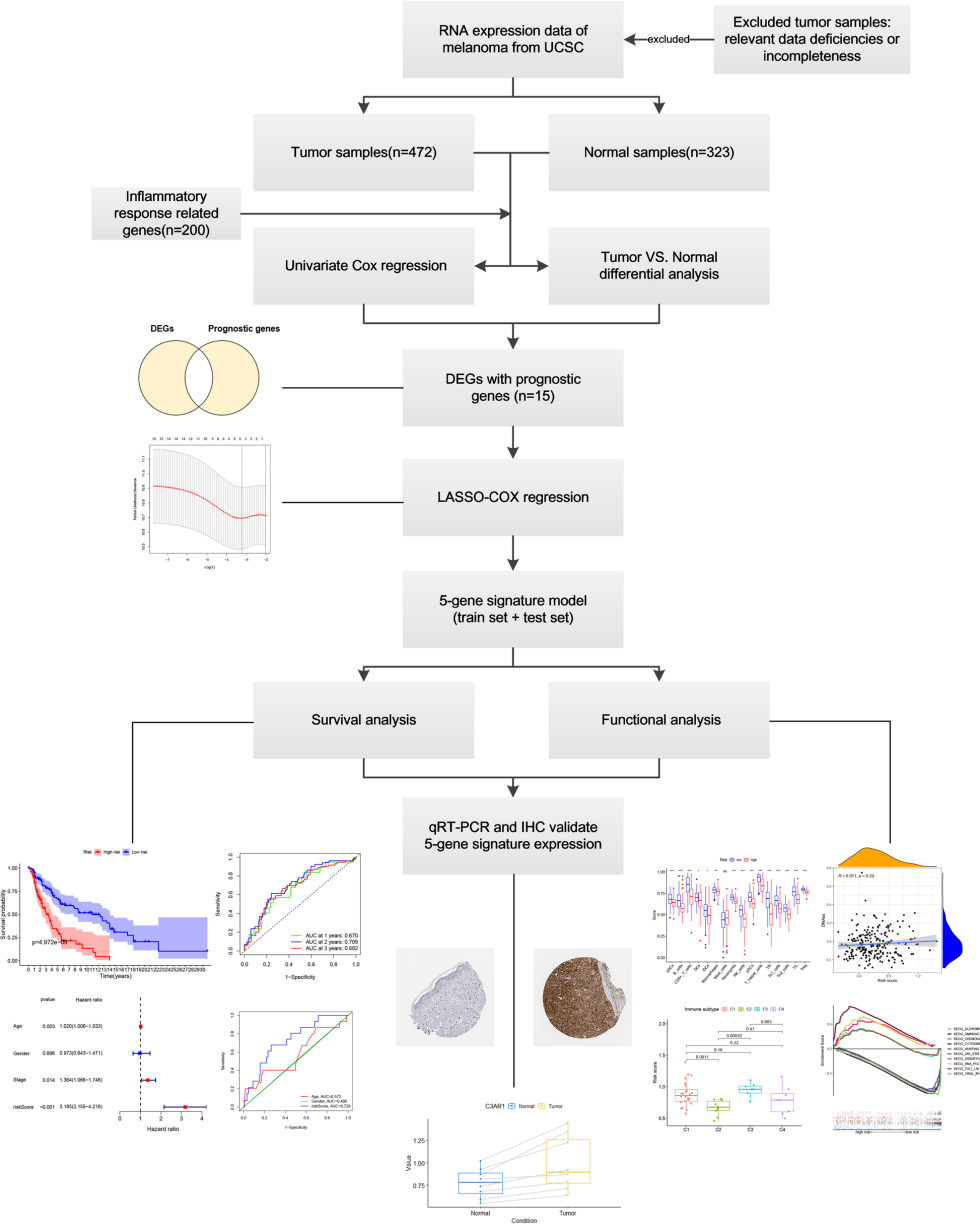
Flow chart of this study, including data collection, analysis, and experiment

The study’s flow chart is depicted in Fig. 1. The study population included 472 patients with CM from the UCSC cohort and 323 patients with normal skin tissue expression data. The clinical characteristics of these patients are summarized in Table 1 (including age, gender, stage, and TNM stage). The screening of prognostic genes associated with inflammation was conducted with data on 200 prognostic genes associated with inflammation received from the GSEA database (Additional file 1).

**Table 1:** Characteristics of patients with skin cutaneous melanoma (SKCM) in TCGA

Variables	Number of cases
Tumor sample	471
Normal sample	1
Age (years): < 60/≥ 60/unknown	252/212/8
Gender: male/female	98/80
Stage: I/II/III/IV/NA	77/140/171/24/60
T: T0/T1/T2/T3/T4/NA	23/42/78/90/154/55
N: N0/N1/N2/N3/NA	235/74/49/56/36
M: M0/M1/NA	418/25/29

### Construction and validation of a predictive model related to inflammatory response

**Fig. 2:**
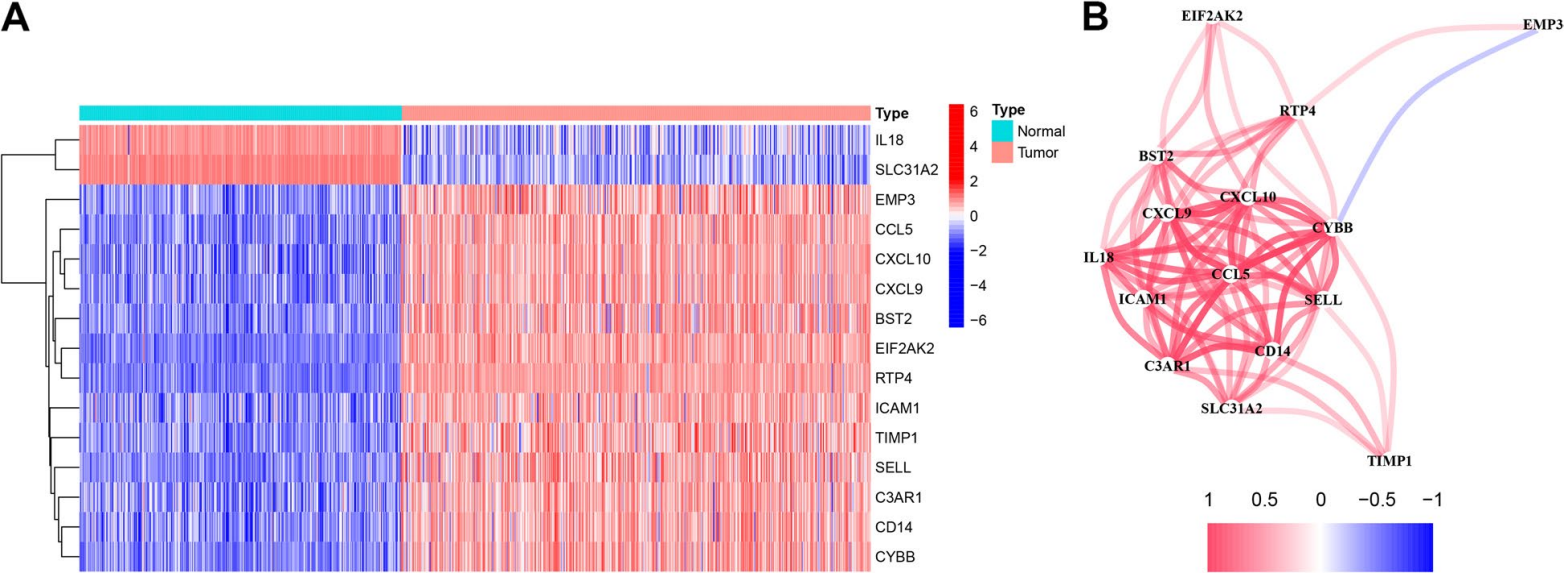
Identification of the candidate inflammatory response-related genes in the UCSC cohort. **A** The 15 overlapping genes expression between cutaneous melanoma and adjacent normal tissues. **B** The correlation network of 15 candidate genes

**Fig. 3:**
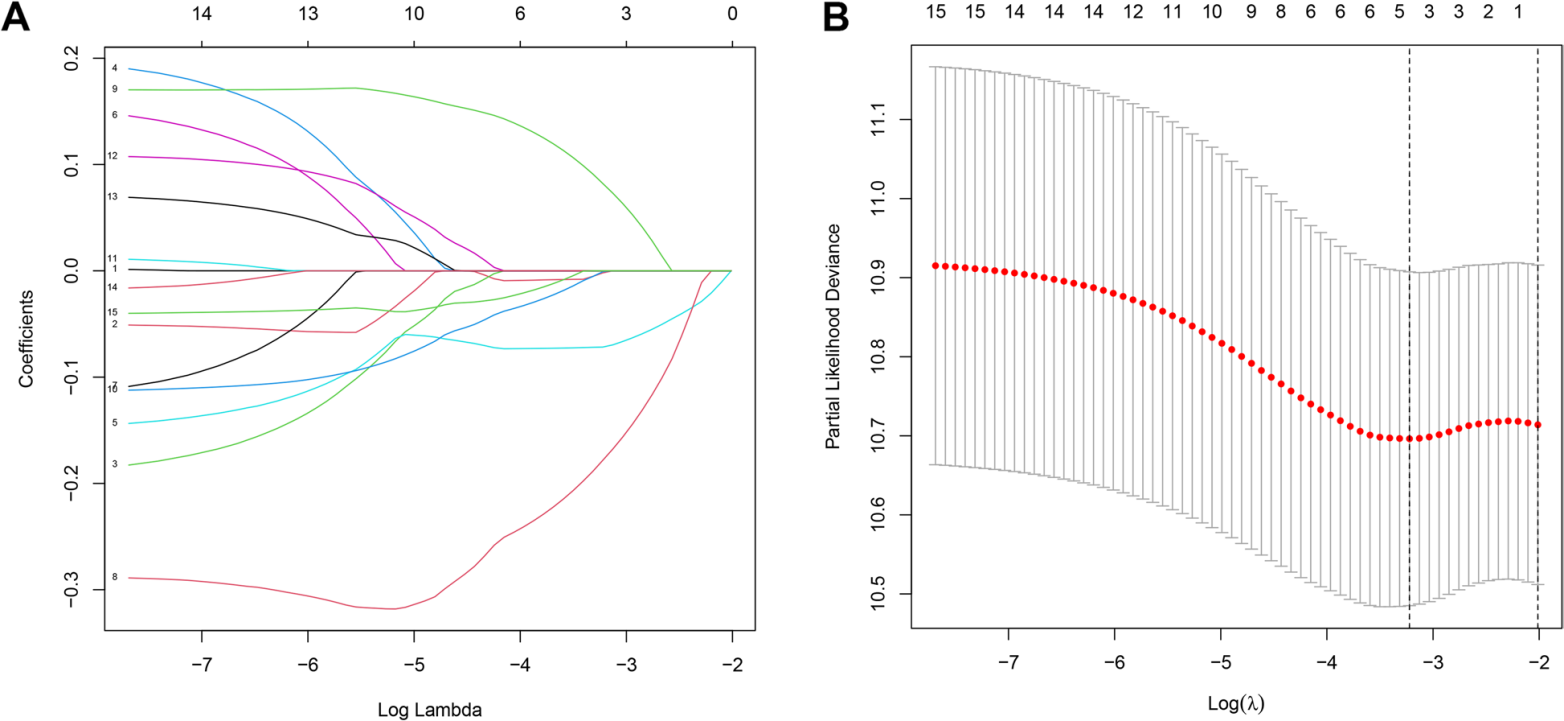
Constructing five-gene-based classifier by LASSO Cox regression model. **A** Trajectory of each independent variable. Horizontal axis represents log of independent variable *λ*. Vertical axis represents coefficient of independent variable. **B** Tenfold cross-validation of tuning parameters in LASSO model

We removed samples with identical ID values and missing clinical data, leaving 454 samples with survival days and survival status. These samples were randomly divided into a training group (*n* = 228) (Additional file 2) and a validation group (*n* = 226) (Additional file 3). We initially screened using one-way Cox analysis and identified 15 inflammatory genes linked with OS (*P* < 0.01) (Fig. 2A) (Additional file 4) and demonstrated their correlation (Fig. 2B). We then performed LASSO regression analysis on these genes and screened inflammatory genes with more than 900 replicates in 1000 replacement samples. We then constructed a model containing five genes to predict the prognosis of CM patients based on their *λ* values (Fig. 3). The model consists of five key genes, including C3AR1, CXCL10, EIF2AK2, EMP3, and ICAM1. The hazard ratio (HR), 95% confidence interval (95% CI), and *P*-value of five hub gene could be seen at Table 2. The risk score is calculated as follows: (−0.00195702921106954) × expression (C3AR1) + (−0.0717393323519231) × expression (CXCL10) + (−0.178228168474953) × expression (EIF2AK2) + (0.0821050910327823) × expression (EMP3) + (−0.000851776035203931) × expression (ICAM1).

**Table 2:** Hazard ratio (HR), 95% confidence interval (95%CI), and *P*-value of hub gene

Hub gene	HR	95% CI	***p***-value
C3AR1	0.79	(0.71, 0.89)	7.70564873394629e-05
CXCL10	0.85	(0.80, 0.90)	9.77794000855977e-08
EIF2AK2	0.71	(0.57, 0.90)	0.00408088388593594
EMP3	1.28	(1.11, 1.46)	0.000421012048490471
ICAM1	0.84	(0.77, 0.93)	0.000392970240176971

**Fig. 4:**
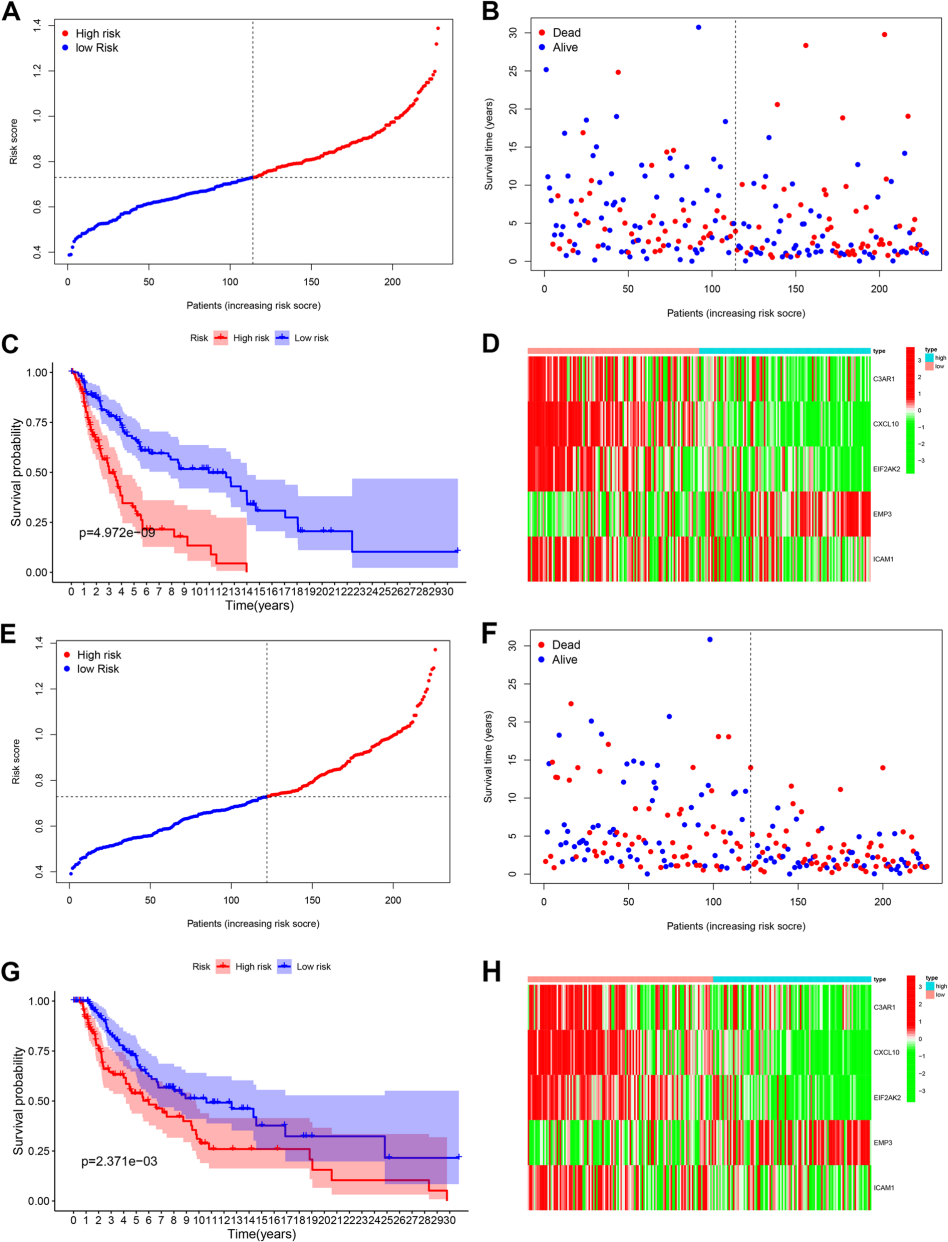
Development and prognostics analysis of 5-gene signature in the training set (**A**, **B**, **C**, **D**) and test set (**E**, **F**, **G**, **H**). **A** and **E** The median value and distribution of the risk score. **B** and **F** The distribution of overall survival (OS) status. **C** and **G** Kaplan-Meier curves for OS of patients in the high-risk and low-risk groups. **D** and **H** Heat map for training group and validation group

**Fig. 5:**
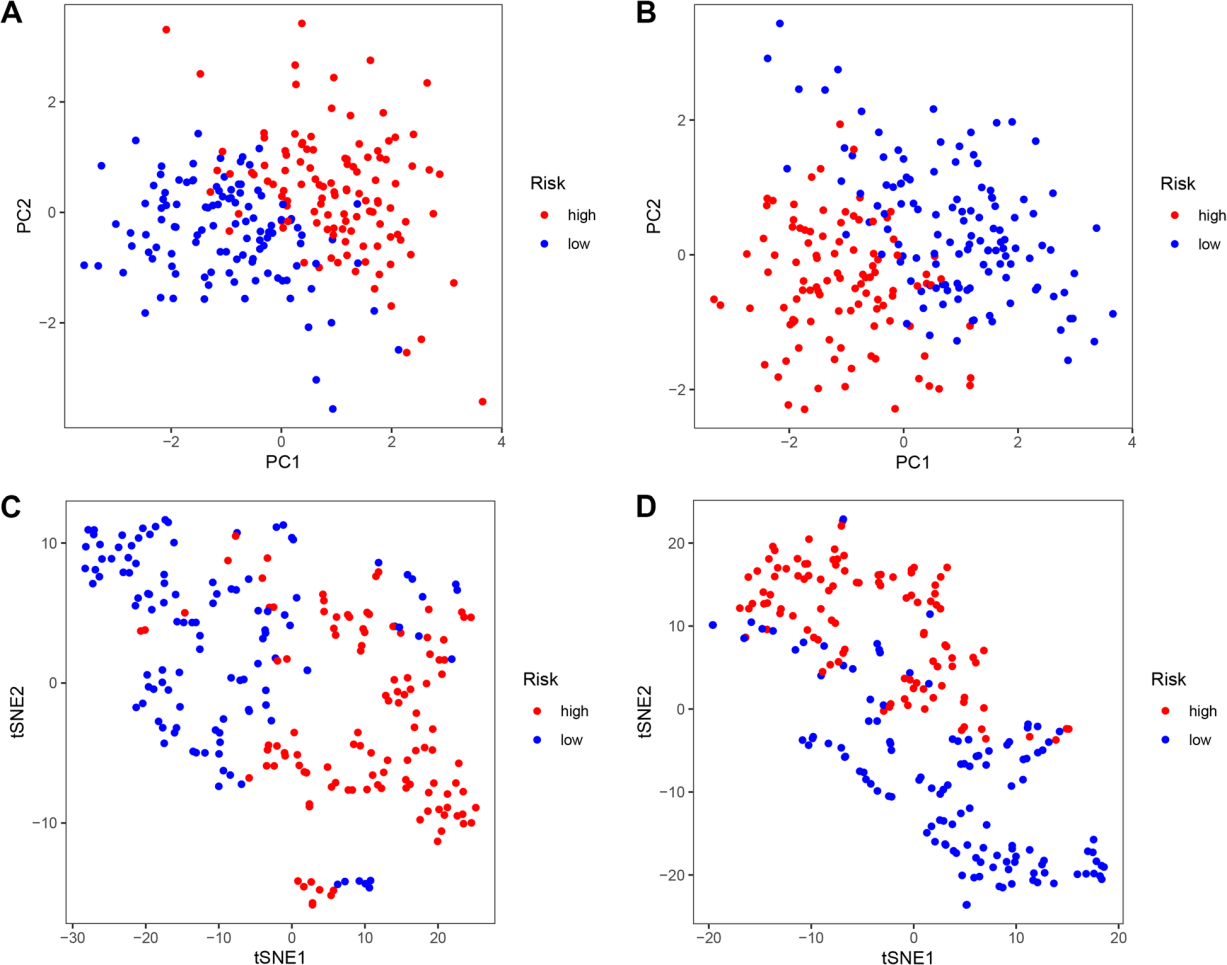
**A** and **B** Principal component analysis (PCA) was performed in training group and validation group, respectively. **C** and **D***t*-Distributed stochastic neighbor embedding (t-SNE) analysis was performed in the training and validation groups, respectively

We split all patients in the training and validation groups into high- and low-risk groups based on the training group’s median risk score. In the training group, we discovered that patients’ chance of death increased, and their survival time reduced (Fig. 4A). Scatter plot analysis revealed that patients classified as high risk had a greater likelihood of dying sooner than patients classified as low risk (Fig. 4B). Kaplan-Meier curves revealed that OS was substantially worse in high-risk individuals than in low-risk patients (*P* < 0.01) (Fig. 4C). The risk heat map depicted the expression of many inflammatory genes associated with prognosis in high- and low-risk groups (Fig. 4D). We next revalidated our results in the validation group, where we noticed substantial variations in OS between the high-risk and low-risk groups (Fig. 4E–G) and inflammatory prognostic gene expression in the heat map (Fig. 4H) proving the model’s accuracy once again. We then used principal component analysis (PCA) (Fig. 5A–B) and t-SNE (Fig. 5C–D) analysis to demonstrate the discrete distribution of patients into different risk groups. Because patients in the high-risk group may die earlier and have shorter survival times than those in the low-risk group, we can intuitively assume that the model constructed using inflammatory prognosis-related genes can better differentiate the prognosis of patients with CM.

### Independent prognostic analysis of OS

**Fig. 6:**
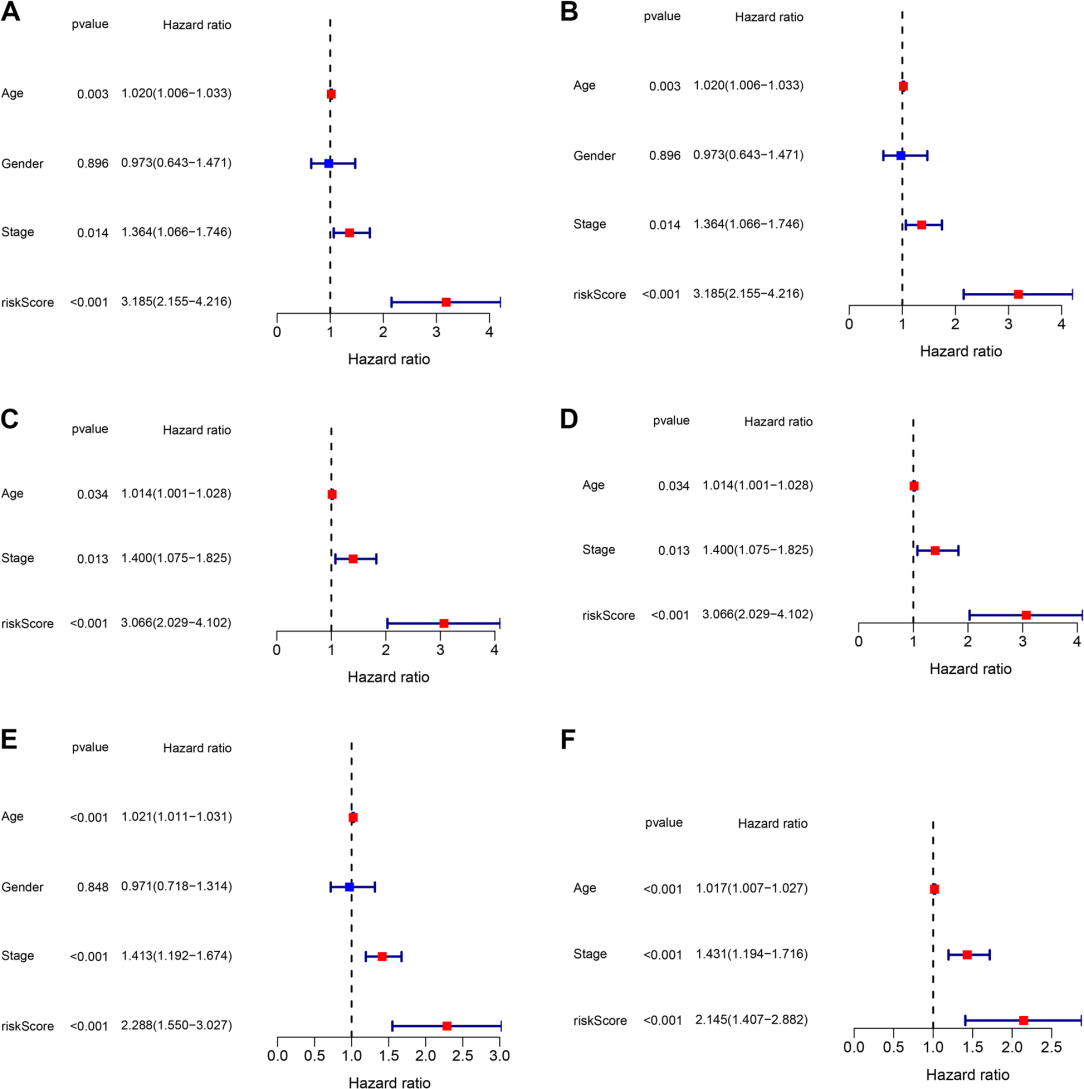
Independent prognostic factors for cutaneous melanoma overall survival (OS). Univariate Cox regression analysis in (**A**, **C**, **E**) for training sets, validation sets, and all samples. Multivariate Cox regression analysis in (**B**, **D**, **F**) for training sets, validation sets, and all samples

**Fig. 7:**
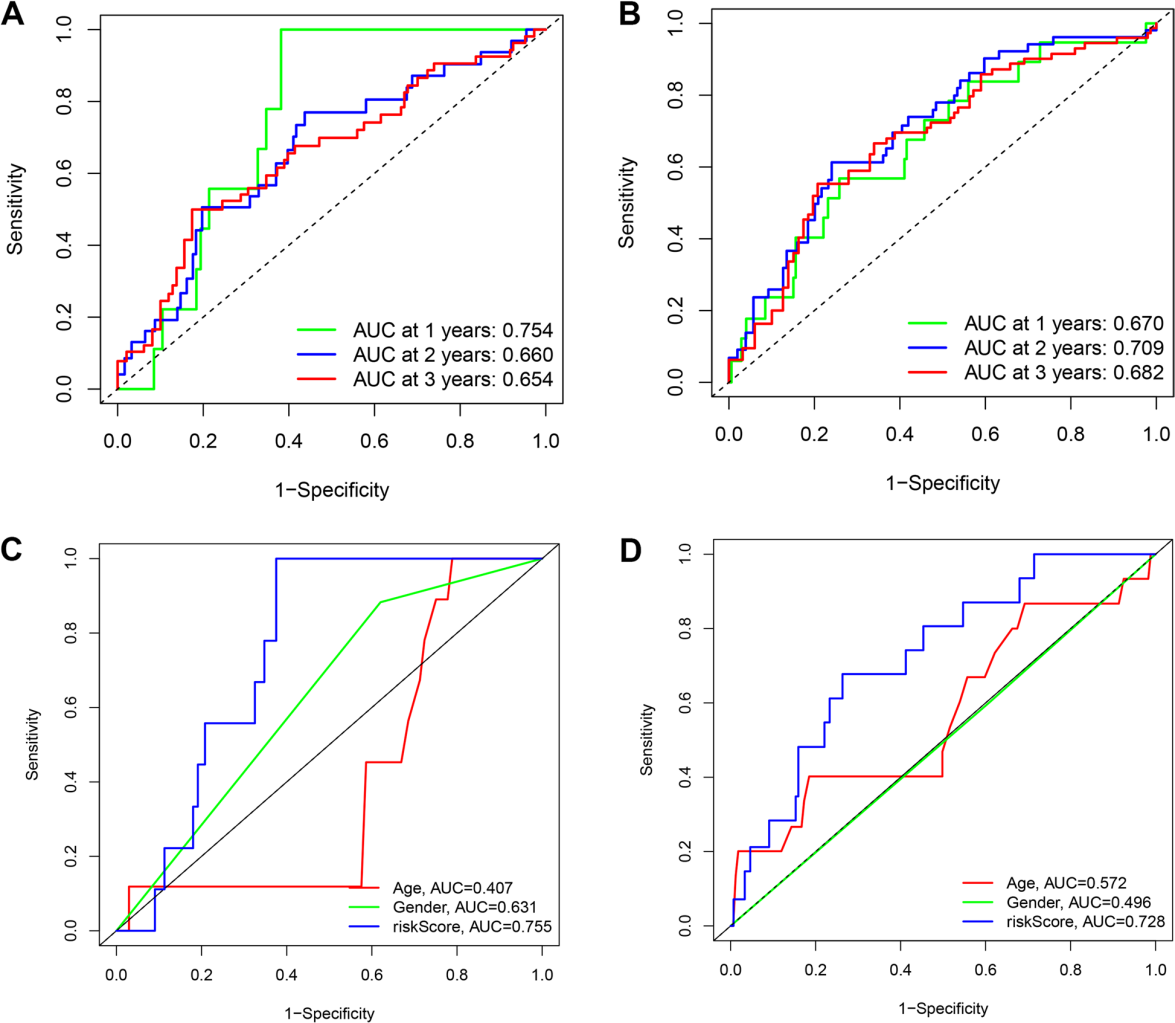
AUC time-dependent receiver operating characteristic curve (ROC) curves. **A** and **B** The ROC analysis of risk scores based on 1-, 2-, and 3-year OS in training and validation groups. **C** and **D** The ROC analysis of risk scores and other clinical characteristics based on OS in training and validation groups

We utilized univariate and multivariate Cox regression analyses to determine if clinical parameters (age, gender), and risk score was significant independent predictors of OS. We showed that risk score, gender, and age were independent predictive predictors of OS in univariate and multivariate Cox regression analyses in the training group (Fig. 6A–B), validation group (Fig. 6C–D), and all samples (Fig. 6E–F). Additionally, the ROC areas at 1, 2, and 3 years were 0.754, 0.660, 0.654 (Fig. 7A), and 0.670, 0.709, 0.682 (Fig. 7B), respectively, for the training and validation groups. Our findings indicated that the risk score model was considerably better in predicting OS in patients with CM than other clinical factors, such as age and gender (Fig. 7C–D).

### Immune status and tumor microenvironment analysis

**Fig. 8:**
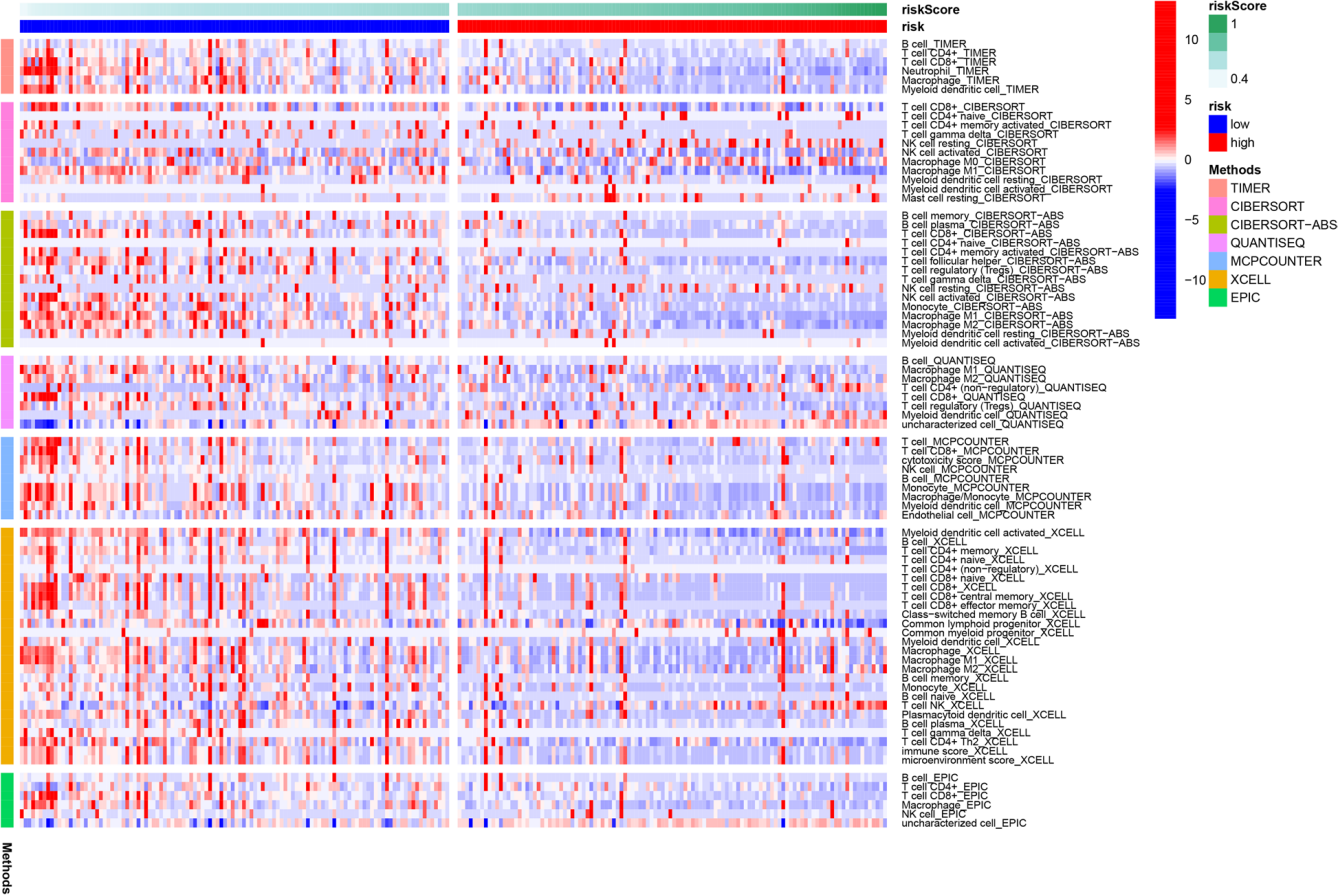
Heat map for immune cell infiltration in training group

**Fig. 9:**
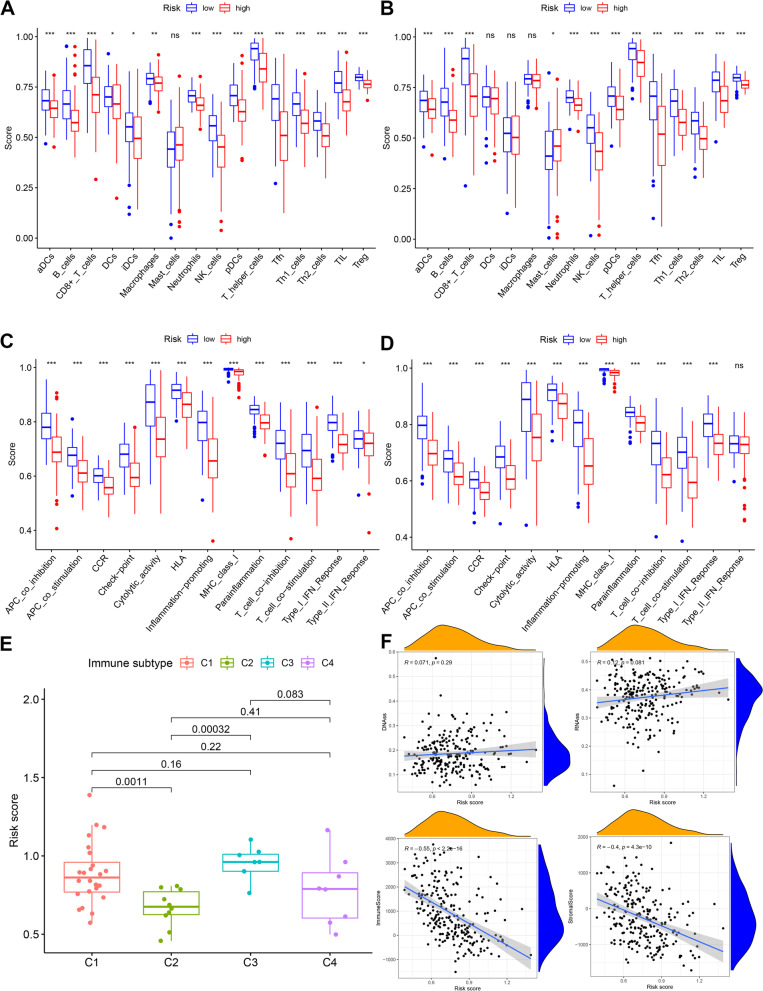
Immune status between different risk groups and the association between risk score and tumor microenvironment. **A** and **B** The scores of 16 immune cells in training set and test set and **C** and **D** 13 immune-related functions in training set and test set were shown in boxplots. **E** Comparison of the risk score in different immune infiltration subtypes. **F** The relationship between risk score and DNAss, RNAss, immuneScore, and stromalScore. Explanation of abbreviated symbols in the figure: aDCs, activated dendritic cells; DCs, dendritic cells; iDCs, immature DCs; pDCs, plasmacytoid dendritic cells; Tfh, follicular helper T cell; TIL, tumor-infiltrating lymphocyte; Tregs, regulatory T cells. *P*-values were shown as follows: ns (not significant); *P < 0.05; **P < 0.01; ***P < 0.001

To further synthesize the immune cell infiltration in CM patients and to explore the differences in immune status between high- and low-risk groups, we applied TIMER, CIBERSORT, CIBERSORT-ABS, QUANTISEQ, MCPCOUNTER, XCELL, and EPIC in the training group, and the results were presented in a heat map (Fig. 8). We then used ssGSEA to quantify the enrichment scores of different immune cell subpopulations, related functions, and pathways to explore the differences in immune cell enrichment scores between the high-risk and low-risk groups. In the training set, we found that the antigen presentation process contained significantly higher levels of aDCs, pDCs, APC-co inhibition, APC-co stimulation, HLA, and MHC class 1 in the low-risk group compared to the high-risk group. In addition, we found that CD8^+^T cells, T helper cells, Tfh cells, Th1 cells, Th2 cells, TIL cells, Treg cells, T-cell co-inhibition, and T-cell co-stimulation associated with T-cell regulation were all significantly higher in the low-risk group than in the high-risk group. The T helper cells, Tfh cells, Th1 cells, Th2 cells, TIL cells, Treg cells, T-cell co-inhibition, and T-cell co-stimulation were all significantly higher than those in the high-risk group, suggesting that there were differences in T-cell regulation between the low-risk and high-risk groups. In addition, we found that the score of CCR and checkpoint, cytolytic activity, B cells, NK cells, neutrophils, inflammation promoting, para-inflammation, type 1 IFN response, and other biological processes and cellular contents were significantly higher in the low-risk group. Response and other biological processes and cellular content were significantly higher than those in the high-risk group (Fig. 9A and C). We found similar results in the validation set as in the training set (Fig. 9B and D).

To examine the association between risk scores and immunological components in further detail, we conducted a correlation analysis between risk scores and immune infiltration. Six distinct immune infiltration types were identified in human tumors, including C1 (wound healing), C2 (IFN-γ dominant), C3 (inflammatory), C4 (lymphocyte depleted), C5 (immunologically quiet), and C6 (TGF-β dominant), although the C5 and C6 immune subtypes were excluded due to their absence in CM patients. We evaluated immune infiltration of CM in the training set and connected it with the risk score, finding that a high-risk score was strongly associated with C3, whereas a low-risk score was significantly associated with C2 (Fig. 9E).

Tumor stemness can be determined using RNA stemness based on mRNA expression (RNAss) or DNA stemness based on DNA methylation pattern (DNAss). Additionally, we evaluated tumor immunological microenvironment using the immuneScore and stromalScore. Correlation analysis examined the relationship between risk score and tumor stemness and immune microenvironment. The results indicated that the risk score was not significantly correlated with DNAss or RNAss but was significantly negatively correlated with immuneScore and stromalScore (*P* < 0.05) (Fig. 9F).

### Biological function and pathway analysis

**Fig. 10:**
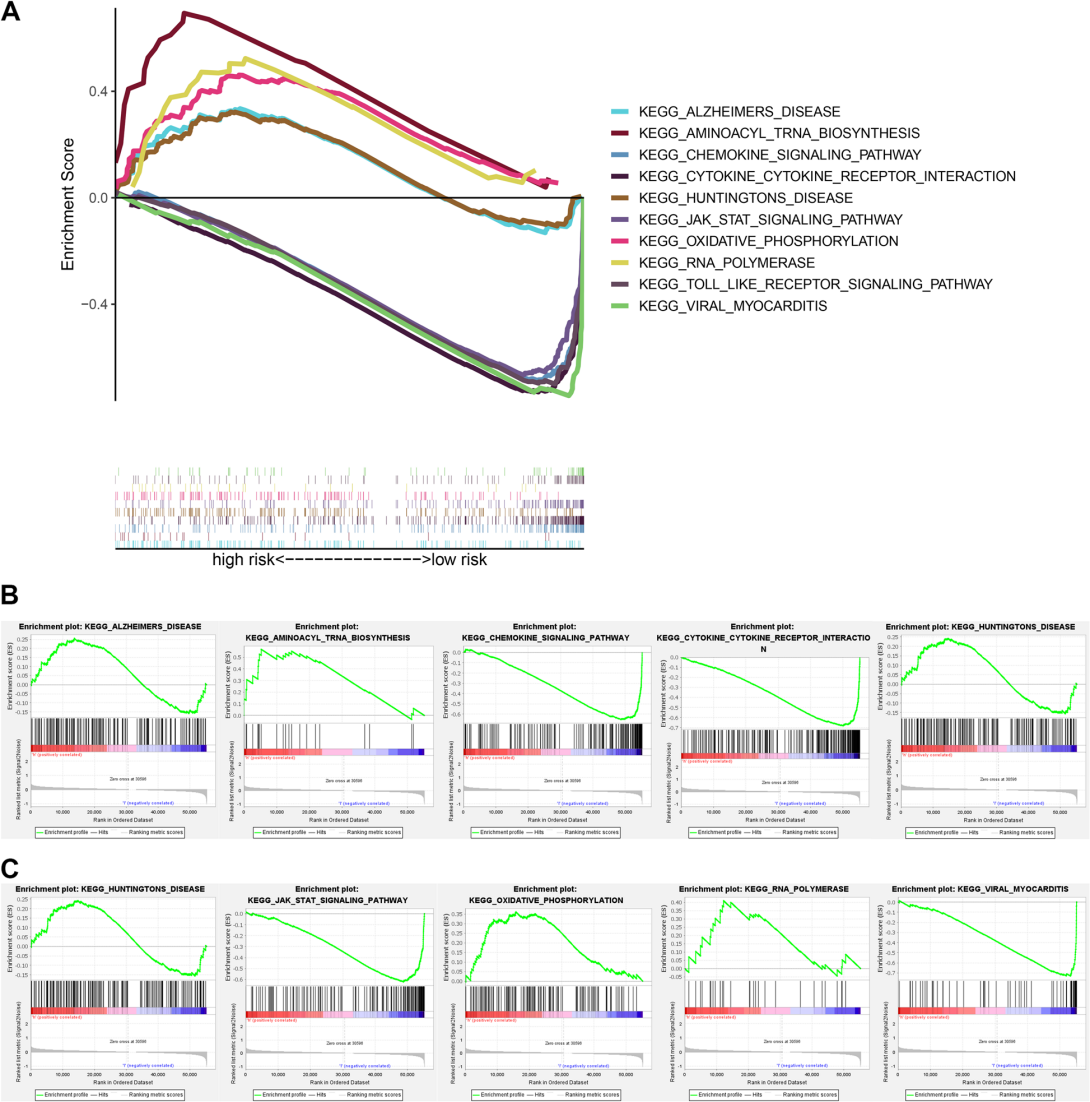
Gene set enrichment analysis (GSEA) of biological functions and pathways. **A** KEGG, Kyoto Encyclopedia of Genes and Genomes. **B** The top 5 significant results of GSEA in high-risk group. **C** The top 5 significant results of GSEA in low-risk group

We used GSEA to conduct enrichment analysis on KEGG pathways in the high- and low-risk group (Fig. 10A). The results analysis revealed that the five pathways with the highest content in the high-risk group were Alzheimer’s disease, aminoacyl tRNA biosynthesis, chemokine signaling pathway, cytokine receptor interaction, and Huntington’s disease (*P* < 0.05, *FDR* < 25%) (Fig. 10B). The JAK-STAT signaling route, oxidative phosphorylation, RNA polymerase, toll-like receptor signaling pathway, and viral myocarditis were the five pathways with the highest levels in the low-risk group (*P* < 0.05, *FDR* < 25%) (Fig. 10C).

### Prognostic gene expression and sensitivity of melanoma cells to chemotherapy

**Fig. 11:**
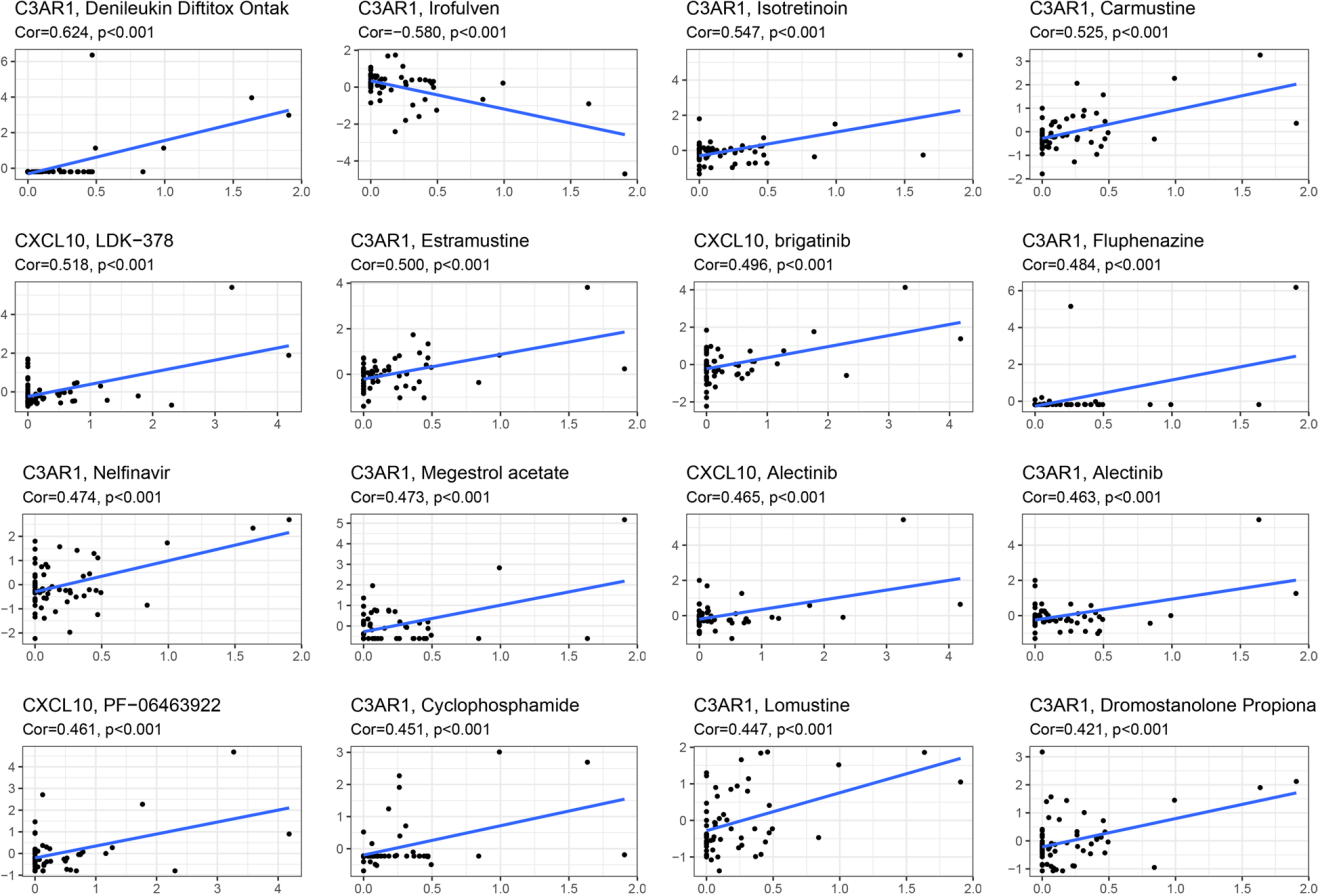
Scatter plot of relationship between prognostic gene expression and drug sensitivity

To make this study more therapeutically relevant, we examined the expression of prognostic genes in NCI-60 cell lines and the association between prognostic gene expression levels and drug sensitivity. The results indicated that all prognostic genes were significantly associated with drug susceptibility to certain chemotherapy agents (*P* < 0.01). The figure depicts the findings of the drug sensitivity analysis with the highest correlation. CXCL10 expression was associated with increased cancer cell resistance to LDK-378, brigatinib, alectinib, and PF-06463922, among others. C3AR1 expression was associated with higher resistance of cancer cells to denileukin diftitox ontak, isotretinoin, carmustine, estramustine, fluphenazine, nelfinavir, megestrol acetate, alectinib, cyclophosphamide, lomustine, and dromostanolone propiona, among others (Fig. 11).

### Validation of key genes in melanoma and paraneoplastic tissues

**Fig. 12:**
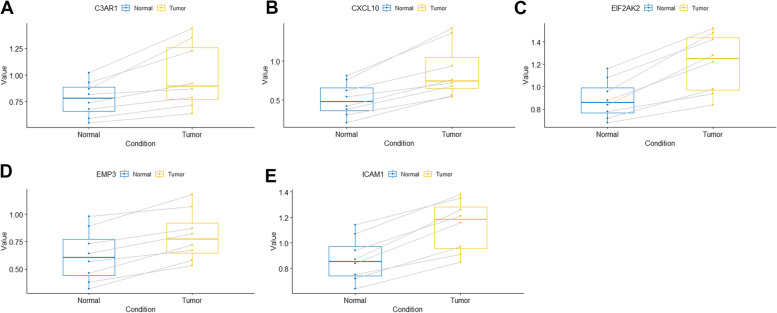
Experiment confirmed the difference of the prognostic gene expression between cutaneous melanoma and adjacent non-tumor tissues. **A**, **B**, **C**, **D**, **E** The mRNA expression analysis by qRT-PCR of C3AR1, CXCL10, EIF2AK2, EMP3, and ICAM1

**Fig. 13:**
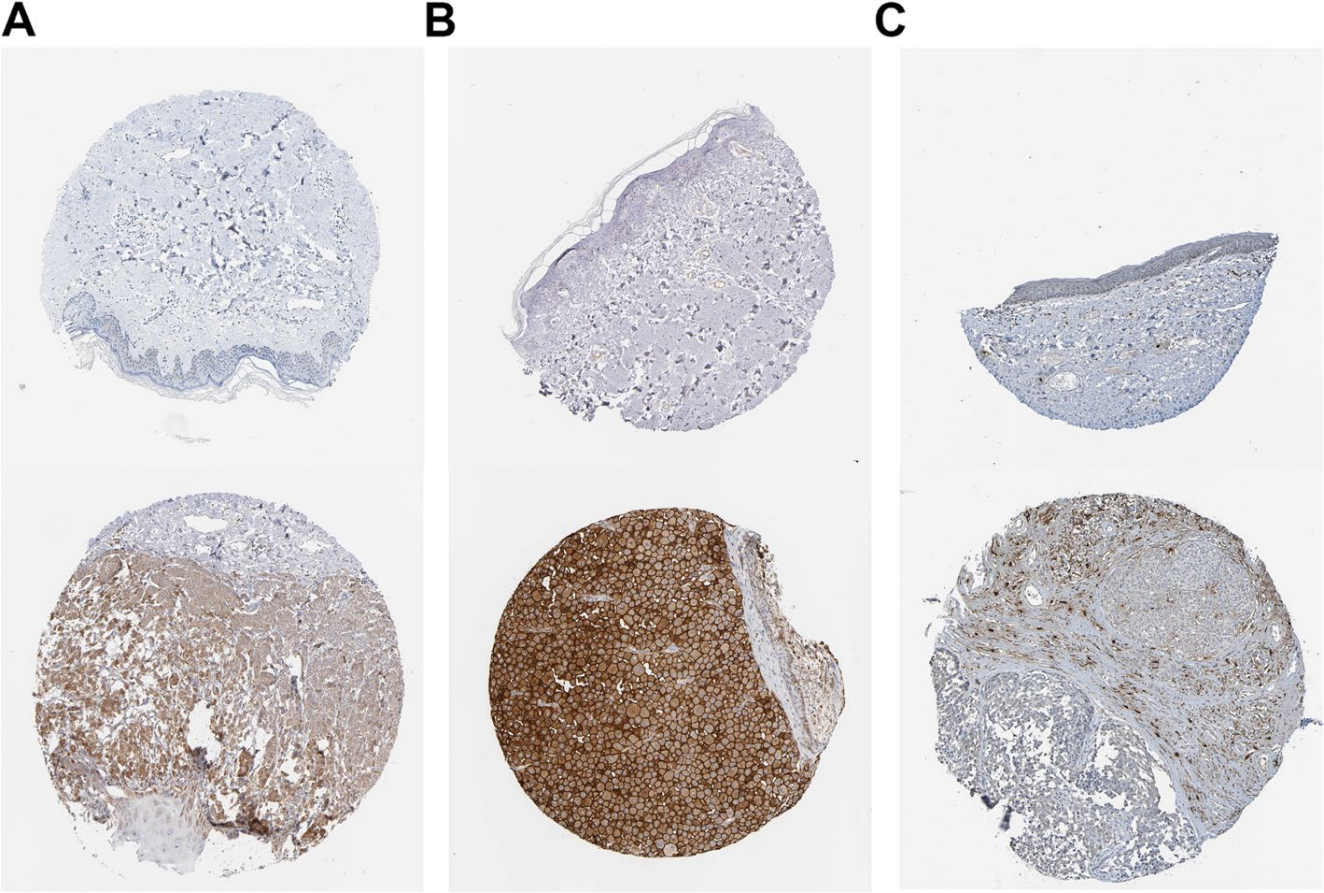
IHC results obtained at Human Protein Atlas (HPA) between melanoma and adjacent non-tumor tissues. **A**, **B**, **C** The IHC results of EIF2AK2, ICAM1, C3AR1

To confirm the changes in expression of 5 important genes (C3AR1, CXCL10, EIF2AK2, EMP3, ICAM1) between CM and para-cancerous normal tissue, we first investigated their mRNA expression using quantitative real-time PCR (qRT-PCR). qRT-PCR analysis revealed that prognostic genes were expressed at a higher level in melanoma than in normal tissues adjacent to malignancy (*P* < 0.01) (Fig. 12A–E). We then validated the expression of key genes in CM and normal skin using the Human Protein Atlas (HPA) database; however, because the HPA database lacked immunohistochemical images of normal and tumor tissues for CXCL10 and EMP3, we validated only the expression of the remaining three key genes, which were all significantly different (Fig. 13A–C).

## Discussion

Cutaneous melanoma (CM), a highly malignant tumor that develops on the skin and mucosa, has increased incidence and increased clinical attention in recent years. Although CM treatment and prognosis have improved in recent years, clinical efficacy has not yet reached the predicted level due to the disease’s complicated genetic and molecular pathways. Apart from the conventional gold standard for pathological diagnosis, we frequently struggle with the early diagnosis and prognosis of CM due to the scarcity of melanoma biomarkers. S100B [27], VEGF (vascular endothelial growth factor) [28], IGF-1R (the insulin-like growth factor 1 receptor) [29], Wnt-5a [30], LDH (lactate dehydrogenase) [31], and MIA (melanoma inhibitory activity) [32] have all been shown to correlate positively with prognosis in CM. In contrast, indicators such as RUNX3 (human runt-related transcription factor 3) [33] have a high degree of accuracy in predicting the prognosis of CM, and traditional inflammation-related serum biomarkers such as neutrophil ratio and lymphocyte/monocyte ratio have also performed well in predicting the prognosis of CM, but no report of inflammatory response-related gene models as prognostic markers of CM has been made. Thus, in this investigation, we attempted to leverage genes associated with the inflammatory response to develop a predictive model for CM patients to enhance overall survival.

This study used multivariate Cox regression analysis to assess five prognostic genes related to inflammation and successfully developed a clinical prediction model for CM. We randomly assigned CM patients to train and validation groups based on median cutoff values and evaluated risk score as an independent risk factor for this prognostic model using univariate and multivariate data analysis of patients in high- and low-risk groups. In univariate and multivariate analyses, we observed that the risk score was more accurate than clinicopathological factors (such as age and gender) in predicting OS in CM patients. The AUC curves demonstrated our model’s excellent predictive performance in training and validation groups. The predictive model created in this work comprises five inflammatory response-related genes: C3AR1, CXCL10, EIF2AK2, EMP3, and ICAM1, all of which are all overexpressed in melanoma tissue and are associated with a poor prognosis. C3AR1 (complement component 3a receptor 1) is a protein-coding gene encodes a component of the complement system’s g protein-coupled transmembrane that spans the C3a receptor [34]. C3AR1 was discovered to promote voluntary exercise behavior in melanoma mice by altering inflammatory and immunological responses in a study conducted by Zhi et al. [35]. Although the effect of C3AR1 expression on melanoma is unknown, a study by Nabizadeh discovered that in the absence of complement C3aR (receptor for complement C3a), the development and growth of B16-F0 melanoma were inhibited in mice. In contrast, C3aR antagonists inhibited the growth of established melanoma [36]. CXCL10 (chemokine ligand 10) is also known as interferon-γ-inducible protein 10 (IP 10). It is a member of the CXC chemokine superfamily and plays a role in immune response regulation, angiogenesis, apoptosis, the cell cycle, and cell proliferation [37, 38]. CXCL10 has been demonstrated to induce apoptosis in HeLa cells by suppressing the production of the HPV oncogenic proteins E6 and E7, consequently promoting the sustained expression of P53 in tumor cells [39]. Additionally, CXCL10 has been shown to decrease estrogen-induced pro-tumor development by inhibiting VEGF production [40]. CXCL10 was discovered to repress tumors and stimulate their development via a variety of mechanisms. Wennerberg et al. discovered that NK cells have a greater capacity to metastasis to melanomas that exhibit positive CXCL10 expression than melanomas that express negative CXCL10 expression, resulting in decreased tumor load and increased survival time [41]. CXCL10 is a powerful angiogenesis inhibitor that binds to CXCR3 receptors and suppresses melanoma’s angiogenesis by decreasing intra-tumor vascular density and boosting apoptosis and necrosis of malignant tissue [42]. EIF2AK2 (eukaryotic translation initiation factors 2 AK2), commonly known as PKR (dsRNA-dependent protein kinase), was initially discovered as a pathogen recognition molecule, with the N-terminal of this protein binding to double-stranded RNA to induce activation of the C-terminal catalytic region [43, 44]. Paola’s study demonstrated that when cancer cells are stimulated with pro-ICD (immunogenic cell death) drugs, PKR, a key mediator of eIF2 phosphorylation, can promote CTR translocation to the surface of melanoma cells by promoting cancer cell death and the release of damage-associated molecular patterns (DAMP) from dead cells [45]. The EMP3 (epithelial membrane protein 3) gene is a member of the TMP22 (peripheral myelin protein) gene family [46]. The EMP3 protein’s primary roles are assumed to be related to cell proliferation, differentiation, activation of the caspase apoptotic pathway, and intercellular connections [47]. Exogenous expression of the EMP3 gene has been found to limit tumor cell proliferation and act as a tumor suppressor gene [48]. It has been demonstrated that mRNAs associated with epithelial cell lineage interactions (EMP3 and EMP1) are frequently overexpressed in uveal melanoma (UM) with a high risk of metastasis, which is thought to promote plasticity in UM cells, thereby increasing their resistance to conventional chemotherapeutic agents [49, 50]. Although ICAM1 (intercellular adhesion molecule 1) is ubiquitous on the cell surface, its overexpression in several cancers promotes tumor cell invasion and metastasis and is inversely connected with patient prognosis [51]. It has been demonstrated that human primary melanoma cells (T1) produce high levels of ICAM1, and that increased ICAM1 expression associated with PI3K/AKT pathway activation can be exploited by metastatic melanoma cells to resist CTL-mediated lysis [52]. ICAM1 has been proposed as a novel target for a range of solid tumors in recent years, including melanoma and mesenchymal thyroid carcinoma (ATC), and has significant research potential [53].

We employed the GSEA technique to further investigate the biological pathways in high- and low-risk groups. Alzheimer’s disease, aminoacyl tRNA production, chemokine signaling, cytokine receptor interaction, and Huntington’s disease were all significantly enriched in the high-risk group. The aminoacyl-tRNA synthetase (ARS) is a critical enzyme that accurately translates the genetic information contained in messenger RNA into the amino acid sequence of proteins. It has a strong effect on cancer through its effects on apoptosis, RNA splicing, and angiogenesis [54]. T-3861174, a prolyl-tRNA synthetase (PRS) inhibitor, was reported to promote apoptosis in melanoma SK-MEL-2 cells by activating the GCN2-ATF4 pathway, and the apoptosis-inducing activity of T-3861174 was eliminated when GCN2 was knocked down [55]. This indicates that various ARS linked with cancers may be valuable therapeutic targets.

Numerous studies have demonstrated that the immune microenvironment plays a critical role in carcinogenesis, and that invading immune cells can operate as tumor growth promoters and inhibitors. To better understand the relationship between immunotherapy, the immune microenvironment, and neoplastic processes, we used the ssGSEA technique to determine the immune cell infiltration status of CM patients. We compared immune cell infiltration status and tumor microenvironment between high- and low-risk groups. We used correlation analysis to examine the relationship between risk scores and tumor stem cells and the immune microenvironment to understand better the prediction model’s potential mechanisms and predictive performance. CD8^+^ T cells and T helper cells were abundant in the low-risk group of patients. By secreting perforin, serine esterase, and lymphotoxin, CD8^+^T (CTL) lymphocytes contribute significantly to tumor clearance. CTL cells recognize target cells via the membrane receptor Ti-CD3 (TCR), which results in guanine and lymphotoxin binding to the CTL membrane. Phospholipase C activation in conjunction with nucleotide-binding proteins completes the activation of second messengers and information transduction, finally resulting in Ca^2+^ release and protein kinase activation, driving tumor cell death and breakdown [56]. T helper cells are activated when they respond with peptide antigens presented by MHC II (major histocompatibility complex), which regulates or assists the immune response by secreting cytokines against tumor cells [57]. The decreased amounts of immune cells such as CTL and T helper cells in the high-risk group show that immune control is disrupted, and antitumor immune function is weakened in this group, which may contribute significantly to their poor prognosis. As a result, we believe that CTL and T helper cells may be critical therapeutic targets for CM patients.

We explored the function of risk scores in the kind of immune infiltration better to understand the relationship between risk scores and immunological components. We discovered that high-risk scores were strongly linked with C3. In contrast, low-risk scores were significantly connected with C2, implying that C3 as a risk factor may promote carcinogenesis and progression, whilst C2 as a protective factor may retard tumorigenesis and progression. This conclusion is consistent with prior findings that C3 and C4, as immune infiltration types with moderate toxicity, are related to infiltration of suppressive immune cell populations, and patients have a poor prognosis [58]. Many researchers now assume that the tumor microenvironment is formed of a network of stromal cells (fibroblasts, vascular cells, and inflammatory immune cells) [13]. Tumor stem cells accelerate the clinical course of malignancies due to their high capacity for self-renewal and invasion, resulting in treatment resistance [59]. As a result of our research, the high infiltration of tumor immune histiocytes in the high-risk group of patients was negatively connected with the immune and stromal scores, which was consistent with the risk score results.

To make this analysis more practically applicable, we examined the connection between important gene expression levels and chemotherapeutic drug sensitivity using data from the cellMiner database for the NCI-60 cell line. Numerous medications have been found in clinical trials to inhibit melanoma growth. Denileukin diftitox ontak, for example, is a diphtheria toxin-based fusion protein that has been licensed for the treatment of persistent skin malignancies such as cutaneous T-cell lymphoma and melanoma via CD25 depletion [60]. Carmustine functions as a chloroethylating nitrosoureas (CNU) by alkylating DNA bases to generate interstrand cross-links (ICL), which inhibit DNA replication and transcription by covalently connecting DNA strands, resulting in severe cytotoxic DNA damage and hence anticancer activity. Because of its potent antitumor properties, Carmustine has been utilized clinically to treat malignant gliomas and melanomas [61]. As a result of our findings, we hypothesize that some prognostic genes could be employed as therapeutic targets to overcome treatment resistance or adjuvant drug sensitivity. We also validated the model’s accuracy by demonstrating the expression levels of important genes in CM and normal skin tissues using qRT-PCR and IHC.

Since Virchow speculated in 1863 that tumors originate from chronic inflammation, there is growing evidence that inflammation plays an essential role in tumorigenesis, development, and evolution. A variety of cytokines produced during inflammation regulate the activation and migration of endothelial cells and their proliferation, survival, and apoptosis, thus playing an essential role in angiogenesis [62]. From our pathway enrichment and immunoassay results, it is clear that patients in the high-risk group have less inflammation-associated cell infiltration, are in an overall low immune infiltration state, have less ability to target tumor clearance, which has more rapid tumor progression, and are more likely to metastasize to distant sites. Therefore, we hypothesize that in CM, as inflammatory infiltration deepens, tumor risk grade becomes higher, overall immune infiltration content becomes lower, and tumors are more likely to spread, metastasize, and recur. We suggest that the inflammatory environment and TME together constitute the tumor ecosphere. Inflammation in TME can accelerate alterations in epigenetic and TME components, jointly promoting tumor development [63]. Hashimoto et al.’s study reported a rare case of undifferentiated pleomorphic sarcoma (UPS) in a high C-reactive protein (CRP) senior patient [64]. Considering that both UPS and CM are in a state of hyper-immune infiltration compared to normal tissue and can be classified as “hot tumors,” we speculate that wide margin resection as soon as possible after exclusion of bacteremia-like infections may be a new option for the treatment of CM in a severe inflammatory state. Schuckmann et al.’s study demonstrated that people who regularly used anti-inflammatory drugs and statins had a much lower rate of CM than those who did not take such drugs [65]. Dipak et al.’s study showed that ketorolac could indirectly stimulate some T cells of the immune system by preferentially inhibiting the COX-1 enzyme, enhancing the immune checkpoint inhibitor’s effect. Ketorolac can eradicate cancer metastasis and prolong survival in mouse models by administering the drug preoperatively [66]. Therefore, we suggest that anti-inflammatory drugs can alter the prognosis of skin tumors. If used as a preventive effect, these drugs need to be taken regularly in daily life. If one already has CM, preoperative administration has been shown in animal models to eradicate cancer metastases and prolong survival, but further confirmation in clinical studies is needed.

This work creates a predictive model for CM patients based on inflammatory response genes. This study, however, has several drawbacks. First, we employed only the UCSC dataset to assess our model’s predictive ability, and we used limited validation approaches due to a lack of sufficient clinical samples. Our model may encourage others to conduct additional research on inflammatory prognostic variables in CM despite its shortcomings.

## Conclusion

In summary, we established a new predictive model based on five inflammatory response genes associated with prognosis (C3AR1, CXCL10, EIF2AK2, EMP3, ICAM1). This prognostic model was demonstrated to be independently linked with overall survival (OS) in the UCSC cohort. It gives new directions and research value in the tumor microenvironment, functional analysis, immune response, and treatment sensitivity. Our research on inflammatory response-related prognostic genes not only elucidates their involvement in cancer but also lays the groundwork for the future development of personalized and precision medicine.

## Supplementary Information

**Additional file 1.**

**Additional file 2.**

**Additional file 3.**

**Additional file 4.**

## Data Availability

All data generated or analyzed during this study are included in this published article and its supplementary files.
