# Supplementary material for: A novel inflammatory response-related signature predicts the prognosis of cutaneous melanoma and the effect of antitumor drugs
Source: World J Surg Oncol. 2022 Aug 19;20:263. doi: 10.1186/s12957-022-02726-8 (PMC9389732; doi:10.1186/s12957-022-02726-8)
Supplement: Supplementary file 1 — Additional file 1. [file 12957_2022_2726_MOESM1_ESM.docx]

| ABCA1 |
| --- |
| ABI1 |
| ACVR1B |
| ACVR2A |
| ADM |
| ADORA2B |
| ADRM1 |
| AHR |
| APLNR |
| AQP9 |
| ATP2A2 |
| ATP2B1 |
| ATP2C1 |
| AXL |
| BDKRB1 |
| BEST1 |
| BST2 |
| BTG2 |
| C3AR1 |
| C5AR1 |
| CALCRL |
| CCL17 |
| CCL2 |
| CCL20 |
| CCL22 |
| CCL24 |
| CCL5 |
| CCL7 |
| CCR7 |
| CCRL2 |
| CD14 |
| CD40 |
| CD48 |
| CD55 |
| CD69 |
| CD70 |
| CD82 |
| CDKN1A |
| CHST2 |
| CLEC5A |
| CMKLR1 |
| CSF1 |
| CSF3 |
| CSF3R |
| CX3CL1 |
| CXCL10 |
| CXCL11 |
| CXCL6 |
| CXCL9 |
| CXCR6 |
| CYBB |
| DCBLD2 |
| EBI3 |
| EDN1 |
| EIF2AK2 |
| EMP3 |
| ADGRE1 |
| EREG |
| F3 |
| FFAR2 |
| FPR1 |
| FZD5 |
| GABBR1 |
| GCH1 |
| GNA15 |
| GNAI3 |
| GP1BA |
| GPC3 |
| GPR132 |
| GPR183 |
| HAS2 |
| HBEGF |
| HIF1A |
| HPN |
| HRH1 |
| ICAM1 |
| ICAM4 |
| ICOSLG |
| IFITM1 |
| IFNAR1 |
| IFNGR2 |
| IL10 |
| IL10RA |
| IL12B |
| IL15 |
| IL15RA |
| IL18 |
| IL18R1 |
| IL18RAP |
| IL1A |
| IL1B |
| IL1R1 |
| IL2RB |
| IL4R |
| IL6 |
| IL7R |
| CXCL8 |
| INHBA |
| IRAK2 |
| IRF1 |
| IRF7 |
| ITGA5 |
| ITGB3 |
| ITGB8 |
| KCNA3 |
| KCNJ2 |
| KCNMB2 |
| KIF1B |
| KLF6 |
| LAMP3 |
| LCK |
| LCP2 |
| LDLR |
| LIF |
| LPAR1 |
| LTA |
| LY6E |
| LYN |
| MARCO |
| MEFV |
| MEP1A |
| MET |
| MMP14 |
| MSR1 |
| MXD1 |
| MYC |
| NAMPT |
| NDP |
| NFKB1 |
| NFKBIA |
| NLRP3 |
| NMI |
| NMUR1 |
| NOD2 |
| NPFFR2 |
| OLR1 |
| OPRK1 |
| OSM |
| OSMR |
| P2RX4 |
| P2RX7 |
| P2RY2 |
| PCDH7 |
| PDE4B |
| PDPN |
| PIK3R5 |
| PLAUR |
| PROK2 |
| PSEN1 |
| PTAFR |
| PTGER2 |
| PTGER4 |
| PTGIR |
| PTPRE |
| PVR |
| RAF1 |
| RASGRP1 |
| RELA |
| RGS1 |
| RGS16 |
| RHOG |
| RIPK2 |
| RNF144B |
| ROS1 |
| RTP4 |
| SCARF1 |
| SCN1B |
| SELE |
| SELL |
| SELENOS |
| SEMA4D |
| SERPINE1 |
| SGMS2 |
| SLAMF1 |
| SLC11A2 |
| SLC1A2 |
| SLC28A2 |
| SLC31A1 |
| SLC31A2 |
| SLC4A4 |
| SLC7A1 |
| SLC7A2 |
| SPHK1 |
| SRI |
| STAB1 |
| TACR1 |
| TACR3 |
| TAPBP |
| TIMP1 |
| TLR1 |
| TLR2 |
| TLR3 |
| TNFAIP6 |
| TNFRSF1B |
| TNFRSF9 |
| TNFSF10 |
| TNFSF15 |
| TNFSF9 |
| TPBG |
| VIP |
