# Supplementary material for: A novel inflammatory response-related signature predicts the prognosis of cutaneous melanoma and the effect of antitumor drugs
Source: World J Surg Oncol. 2022 Aug 19;20:263. doi: 10.1186/s12957-022-02726-8 (PMC9389732; doi:10.1186/s12957-022-02726-8)
Supplement: Supplementary file 2 — Additional file 2. [file 12957_2022_2726_MOESM2_ESM.docx]

| id | futime | fustat | BST2 | C3AR1 | CCL5 | CD14 | CXCL10 | CXCL9 | CYBB | EIF2AK2 | EMP3 | ICAM1 | IL18 | RTP4 | SELL | SLC31A2 | TIMP1 |
| --- | --- | --- | --- | --- | --- | --- | --- | --- | --- | --- | --- | --- | --- | --- | --- | --- | --- |
| TCGA-FS-A1ZH | 2.728767 | 1 | 6.828652 | 4.117188 | 6.153608 | 8.092881 | 5.603768 | 6.321701 | 5.481674 | 3.068983 | 5.926901 | 5.377476 | 2.22319 | 2.733912 | 4.328878 | 1.169288 | 11.50263 |
| TCGA-D3-A51H | 4.69589 | 0 | 6.948914 | 3.666355 | 6.811269 | 6.332961 | 5.630144 | 6.569226 | 5.841767 | 2.860083 | 6.380644 | 4.999167 | 4.720179 | 3.944198 | 8.033017 | 0.586463 | 9.581285 |
| TCGA-EE-A2GT | 3.739726 | 0 | 6.386552 | 3.445272 | 4.893636 | 5.35057 | 2.207664 | 3.507138 | 4.122085 | 2.199743 | 8.236801 | 6.034935 | 2.493793 | 3.052128 | 6.361381 | 0.898788 | 8.421176 |
| TCGA-EE-A29Q | 5.561644 | 1 | 5.159824 | 1.695836 | 2.517703 | 4.937072 | 1.252989 | 0.827987 | 2.517423 | 3.662078 | 7.363574 | 5.551702 | 0.836701 | 4.002489 | 1.488006 | 0.382584 | 8.733064 |
| TCGA-D3-A5GO | 11.49315 | 0 | 8.681574 | 2.790574 | 5.698423 | 6.229382 | 6.791197 | 6.666452 | 3.340108 | 3.897135 | 8.033017 | 6.092531 | 2.225587 | 5.141647 | 2.556616 | 1.402197 | 9.947093 |
| TCGA-EE-A2MJ | 8.019178 | 1 | 9.13588 | 3.872675 | 6.882696 | 6.608574 | 7.300321 | 9.289949 | 5.069255 | 3.702098 | 6.766316 | 6.779902 | 2.573711 | 4.407194 | 5.787002 | 1.09508 | 9.241958 |
| TCGA-EB-A44O | 0.221918 | 0 | 8.799477 | 2.284301 | 2.581299 | 2.964514 | 2.710279 | 3.379586 | 1.906004 | 3.95236 | 6.138973 | 6.60147 | 1.207877 | 2.863812 | 2.344824 | 0.978885 | 8.865828 |
| TCGA-D9-A4Z2 | 0.520548 | 1 | 7.48764 | 2.168596 | 3.624704 | 5.662878 | 3.175691 | 2.903462 | 2.848119 | 3.074562 | 6.637062 | 6.055891 | 0.530635 | 3.673846 | 2.367314 | 0.087297 | 10.01891 |
| TCGA-GN-A26C | 2.249315 | 1 | 9.103504 | 4.671911 | 8.346721 | 8.206697 | 6.704789 | 7.698557 | 6.083569 | 4.581065 | 6.481044 | 7.193503 | 4.030538 | 5.072697 | 3.913889 | 1.458677 | 10.2121 |
| TCGA-D3-A8GS | 9.764384 | 1 | 9.336361 | 2.951455 | 6.992529 | 7.037601 | 4.858121 | 5.939048 | 3.633494 | 2.940823 | 7.660811 | 5.025705 | 2.961364 | 5.029074 | 2.628023 | 0.341128 | 9.463852 |
| TCGA-GN-A267 | 5.369863 | 1 | 8.496625 | 2.3519 | 6.163384 | 5.342788 | 5.312731 | 4.580553 | 2.907161 | 3.442284 | 7.39195 | 5.124149 | 1.852851 | 5.293808 | 2.379128 | 0.289768 | 8.720292 |
| TCGA-EE-A20F | 7.630137 | 0 | 8.370865 | 3.415061 | 5.463601 | 6.370978 | 5.535564 | 5.801818 | 4.369894 | 3.080498 | 6.955331 | 6.90424 | 2.155984 | 5.391631 | 2.63959 | 0.76972 | 9.669984 |
| TCGA-ER-A3PL | 2.767123 | 0 | 8.187025 | 3.656082 | 4.278234 | 5.995377 | 4.676105 | 3.873056 | 3.087763 | 3.843007 | 6.802687 | 5.063013 | 1.525813 | 4.226292 | 1.197694 | 0.60849 | 8.980385 |
| TCGA-FS-A1ZN | 2 | 1 | 4.965549 | 1.847202 | 2.243294 | 4.47279 | 1.869851 | 1.457003 | 1.523153 | 3.293153 | 7.363574 | 5.263841 | 1.906004 | 4.73166 | 2.534379 | 0.65744 | 8.907748 |
| TCGA-RP-A694 | 0.057534 | 0 | 8.247378 | 3.33482 | 4.657104 | 5.413919 | 4.753061 | 4.721242 | 2.669225 | 3.159744 | 7.541294 | 5.178268 | 2.196151 | 3.592759 | 2.056783 | 0.949754 | 7.958578 |
| TCGA-D3-A1Q5 | 9.380822 | 1 | 7.824028 | 0.895342 | 3.597626 | 2.586814 | 2.356773 | 0.88697 | 0.559646 | 3.030263 | 6.796978 | 1.991747 | 0.279299 | 1.350467 | 1.99776 | 0.193201 | 12.64537 |
| TCGA-EE-A29X | 1.493151 | 1 | 7.601866 | 4.124118 | 5.971011 | 6.68665 | 3.749969 | 4.406265 | 3.991637 | 3.276915 | 6.744385 | 7.874921 | 1.676536 | 3.83855 | 3.378214 | 1.05789 | 10.15165 |
| TCGA-DA-A1I5 | 11.25205 | 0 | 7.958578 | 4.064035 | 6.386552 | 7.230837 | 5.799272 | 7.111111 | 4.785047 | 3.360324 | 7.456423 | 5.406713 | 3.385223 | 3.410681 | 4.112284 | 1.638293 | 10.12502 |
| TCGA-FW-A3I3 | 1.454795 | 0 | 8.786252 | 1.813823 | 3.076071 | 4.41948 | 2.810461 | 2.054383 | 1.935173 | 3.160056 | 7.070036 | 5.335305 | 2.42424 | 4.193675 | 0.611062 | 0.190191 | 7.445703 |
| TCGA-IH-A3EA | 1.435616 | 0 | 8.101991 | 2.954205 | 4.559768 | 5.365966 | 1.748549 | 1.799133 | 2.382689 | 2.546999 | 9.241958 | 6.430613 | 1.81238 | 3.709693 | 0.910252 | 0.576186 | 10.09768 |
| TCGA-WE-A8JZ | 2.00274 | 0 | 6.710106 | 1.856127 | 3.549904 | 3.495602 | 3.272038 | 4.309414 | 2.045685 | 3.375532 | 6.478848 | 5.232125 | 0.799782 | 3.358694 | 1.868358 | 0.390265 | 7.769483 |
| TCGA-BF-A9VF | 1.205479 | 0 | 3.078942 | 2.147561 | 3.852581 | 3.048013 | 1.160472 | 1.056694 | 0.714127 | 2.542766 | 8.839039 | 5.49616 | 2.241188 | 3.175368 | 0.270052 | 0.293755 | 7.513933 |
| TCGA-EE-A29W | 16.25205 | 0 | 4.624495 | 2.607385 | 2.6359 | 5.51091 | 0.982756 | 0.881394 | 2.98068 | 3.520395 | 5.569785 | 2.115523 | 0.849715 | 1.363033 | 0.897891 | 0.367426 | 9.072918 |
| TCGA-Z2-AA3V | 1.331507 | 0 | 9.352444 | 4.382735 | 6.72588 | 8.720292 | 3.50246 | 5.645365 | 4.300165 | 2.159908 | 8.733064 | 5.790685 | 2.518655 | 3.363006 | 4.332411 | 1.785208 | 9.415135 |
| TCGA-D3-A3CC | 7.243836 | 0 | 4.192048 | 3.355732 | 2.531061 | 6.075771 | 2.066298 | 1.811778 | 3.810748 | 3.301597 | 6.206777 | 4.882811 | 1.842462 | 3.615081 | 1.177324 | 0.978047 | 7.382795 |
| TCGA-BF-AAP6 | 0.890411 | 0 | 8.681574 | 1.90542 | 4.323187 | 4.047255 | 1.923731 | 1.156086 | 1.959274 | 3.626445 | 8.268444 | 5.930877 | 0.498978 | 3.531406 | 0.529275 | 0.358539 | 9.463852 |
| TCGA-ER-A2NH | 3.463014 | 0 | 8.279393 | 4.845757 | 8.922381 | 7.397015 | 8.408325 | 9.180879 | 5.930877 | 3.68983 | 6.210251 | 6.766316 | 3.609812 | 4.656055 | 4.299313 | 1.630502 | 10.58227 |
| TCGA-D3-A5GS | 1.515068 | 0 | 5.668237 | 4.010395 | 5.379266 | 6.094075 | 6.768916 | 5.612279 | 4.179658 | 2.928942 | 7.711737 | 6.174995 | 2.536174 | 4.851699 | 3.059609 | 1.369607 | 9.688332 |
| TCGA-D3-A3C7 | 3.915068 | 0 | 8.852298 | 4.469953 | 6.259385 | 7.508591 | 4.331117 | 6.116462 | 5.137198 | 2.751416 | 7.0518 | 4.435321 | 3.656786 | 3.265845 | 3.219191 | 1.689397 | 12.96557 |
| TCGA-GN-A26D | 4 | 1 | 3.487843 | 2.558081 | 3.527986 | 4.733881 | 2.81385 | 2.111237 | 2.219322 | 2.962001 | 8.334715 | 6.476693 | 1.310385 | 3.204402 | 2.260167 | 1.083936 | 9.103504 |
| TCGA-EE-A3J7 | 5.339726 | 0 | 7.205721 | 2.07565 | 4.336494 | 4.398 | 4.014395 | 3.341721 | 2.178183 | 3.423098 | 6.834409 | 5.779842 | 0.449226 | 4.275192 | 1.827806 | 0.536477 | 6.530735 |
| TCGA-EE-A29P | 4.70137 | 0 | 9.598808 | 4.400788 | 8.656072 | 7.730674 | 9.970523 | 9.43193 | 6.060614 | 3.67064 | 7.666866 | 7.83865 | 3.608779 | 4.681845 | 4.034102 | 1.520048 | 9.15014 |
| TCGA-EB-A51B | 2.550685 | 0 | 5.640952 | 1.151822 | 4.632232 | 4.015587 | 7.743395 | 6.110043 | 1.613995 | 3.587873 | 8.839039 | 6.314155 | 1.175606 | 4.008469 | 0.415861 | 0.277371 | 8.643297 |
| TCGA-OD-A75X | 24.82466 | 1 | 9.103504 | 2.57586 | 3.731903 | 5.331182 | 5.914582 | 3.920829 | 3.064919 | 3.532413 | 6.788251 | 6.749759 | 1.500604 | 4.611089 | 1.574957 | 0.711214 | 8.024331 |
| TCGA-FR-A7U9 | 1.564384 | 0 | 7.256045 | 4.898442 | 6.074119 | 6.913876 | 6.048457 | 5.476823 | 5.08951 | 3.38822 | 8.334715 | 4.056013 | 2.730517 | 3.320675 | 3.398056 | 2.376691 | 10.67807 |
| TCGA-FS-A4FC | 4.534247 | 1 | 5.266222 | 2.424708 | 4.290079 | 5.031132 | 4.595878 | 5.088801 | 3.348831 | 3.900577 | 8.226828 | 3.037887 | 2.544037 | 4.827075 | 2.956716 | 0.754631 | 9.634608 |
| TCGA-D3-A1QA | 7.575342 | 0 | 8.922381 | 2.778497 | 4.290517 | 4.022257 | 4.570606 | 3.424464 | 2.748343 | 4.708191 | 7.584319 | 6.418429 | 1.188537 | 4.310793 | 2.174924 | 0.67101 | 7.776094 |
| TCGA-D3-A8GM | 8.928767 | 1 | 8.226828 | 2.391643 | 5.332848 | 5.545684 | 4.605463 | 5.835481 | 3.66457 | 4.762519 | 6.89809 | 5.573823 | 1.856485 | 4.874369 | 2.713051 | 1.159295 | 9.196354 |
| TCGA-D3-A2JH | 3.506849 | 0 | 9.180879 | 4.209291 | 9.057216 | 6.321701 | 6.945637 | 7.999387 | 5.056886 | 4.309414 | 6.712726 | 6.696849 | 3.139319 | 4.975733 | 3.981492 | 1.427992 | 7.83865 |
| TCGA-EB-A5UN | 4.909589 | 0 | 9.289949 | 3.636598 | 4.182651 | 4.176259 | 2.853365 | 1.982202 | 1.850738 | 3.546122 | 7.904414 | 8.247378 | 0.361618 | 4.85405 | 2.587113 | 0.93108 | 10.41592 |
| TCGA-D3-A3MR | 8.632877 | 0 | 8.950546 | 3.541347 | 8.70773 | 6.94248 | 6.264617 | 7.809954 | 3.845553 | 2.724625 | 7.359111 | 6.426532 | 3.945325 | 4.321881 | 4.42688 | 0.254808 | 10.04373 |
| TCGA-FS-A1ZD | 4.460274 | 1 | 3.466861 | 2.901289 | 1.404704 | 6.483103 | 0.961334 | 0.795989 | 4.019542 | 3.725191 | 7.291223 | 2.928639 | 1.107242 | 0.363093 | 0.570262 | 0.629425 | 8.759469 |
| TCGA-ER-A42L | 12.41918 | 0 | 8.980385 | 5.071336 | 7.692498 | 8.630449 | 5.338646 | 6.499815 | 5.668237 | 2.811055 | 6.876569 | 6.122744 | 3.15017 | 3.278877 | 3.557806 | 1.615407 | 9.598808 |
| TCGA-EB-A4OZ | 1.69863 | 0 | 8.656072 | 3.11524 | 7.402129 | 6.317951 | 5.62693 | 5.76205 | 3.422461 | 3.068983 | 9.103504 | 4.595878 | 4.115513 | 3.939999 | 3.773381 | 0.489295 | 10.15165 |
| TCGA-D3-A1Q4 | 9.336986 | 0 | 5.026384 | 3.506175 | 3.533799 | 5.826608 | 2.642969 | 3.917367 | 3.983397 | 3.693379 | 6.134201 | 5.376598 | 1.358154 | 1.474722 | 3.194721 | 0.56524 | 9.415135 |
| TCGA-EE-A2MR | 11.2 | 0 | 6.424496 | 5.088801 | 6.785484 | 8.075346 | 6.020281 | 6.718114 | 6.837192 | 4.185614 | 6.062019 | 5.429472 | 4.104206 | 3.311857 | 6.639493 | 1.241844 | 9.799361 |
| TCGA-EE-A3AD | 2.39726 | 1 | 5.874798 | 1.173014 | 2.749233 | 3.609812 | 1.752814 | 1.183077 | 0.963794 | 3.296091 | 6.618162 | 6.689249 | 0.643565 | 4.15954 | 2.169191 | 0.319989 | 9.196354 |
| TCGA-EE-A3JH | 11.19452 | 0 | 8.681574 | 4.795918 | 7.942817 | 7.567918 | 6.466031 | 7.749891 | 5.766811 | 3.308653 | 7.378207 | 6.707448 | 4.029376 | 4.650217 | 5.655324 | 1.776299 | 9.119603 |
| TCGA-FS-A4F8 | 14.56986 | 1 | 7.256045 | 2.957951 | 6.196628 | 5.907864 | 5.068561 | 6.266454 | 4.790193 | 3.401107 | 6.907455 | 5.394217 | 2.971296 | 3.068354 | 6.707448 | 0.45861 | 9.448603 |
| TCGA-ER-A2NC | 3.652055 | 1 | 8.016265 | 4.985434 | 4.24821 | 8.980385 | 2.971606 | 1.900096 | 5.804256 | 3.355732 | 5.739733 | 5.078265 | 2.664432 | 2.280372 | 2.458693 | 0.984279 | 10.15165 |
| TCGA-D9-A4Z3 | 1.383562 | 0 | 8.907748 | 3.28342 | 4.265761 | 6.165024 | 3.09094 | 2.553906 | 3.920829 | 2.764631 | 7.129786 | 5.07548 | 1.43269 | 1.062172 | 0.590769 | 1.206357 | 7.93477 |
| TCGA-BF-A5EQ | 0.884932 | 0 | 7.666866 | 2.814485 | 5.476823 | 5.943017 | 4.72839 | 5.705264 | 3.012722 | 3.219532 | 7.867894 | 6.550894 | 1.629695 | 4.697916 | 3.462447 | 0.433174 | 9.13588 |
| TCGA-D3-A8GL | 7.427397 | 1 | 7.803093 | 1.19915 | 1.23504 | 2.770188 | 1.055631 | 0.571435 | 1.058081 | 4.076559 | 7.354337 | 5.001813 | 0.28775 | 3.756474 | 1.289682 | 0.277036 | 6.696849 |
| TCGA-EE-A17Y | 2.268493 | 1 | 3.570924 | 4.597876 | 2.483741 | 4.568102 | 1.955699 | 2.104298 | 1.742047 | 3.477691 | 8.496625 | 5.038563 | 0.650898 | 2.252077 | 1.719819 | 0.443142 | 10.34278 |
| TCGA-DA-A3F5 | 18.83014 | 1 | 7.769483 | 2.509507 | 3.543331 | 3.810927 | 1.333667 | 2.006771 | 2.730844 | 3.290881 | 6.945637 | 3.250895 | 0.785193 | 2.082923 | 2.910248 | 0.752623 | 10.89923 |
| TCGA-BF-A5EP | 0.917808 | 0 | 8.359438 | 2.111594 | 2.705028 | 3.893662 | 1.749692 | 1.62848 | 1.306765 | 3.21513 | 7.853397 | 5.099338 | 0.826876 | 2.974107 | 0.705652 | 0.456532 | 9.780437 |
| TCGA-HR-A2OG | 0.019178 | 0 | 8.433625 | 3.89022 | 5.70068 | 7.304577 | 5.619622 | 6.571463 | 4.769267 | 2.916799 | 6.656557 | 7.041155 | 1.936052 | 3.262571 | 2.319848 | 0.743212 | 10.18136 |
| TCGA-FS-A4FD | 6.723288 | 1 | 7.692498 | 2.328231 | 4.335609 | 5.054897 | 4.829938 | 4.87736 | 3.09347 | 3.125338 | 6.21372 | 4.660767 | 1.385229 | 2.012808 | 1.106972 | 0.643706 | 6.861068 |
| TCGA-EE-A2MK | 15.03288 | 0 | 9.258211 | 4.158307 | 7.062695 | 8.016265 | 6.779902 | 7.363574 | 5.593221 | 3.359346 | 6.153608 | 7.824028 | 2.732703 | 4.8022 | 2.522569 | 1.079139 | 10.18136 |
| TCGA-FS-A1ZC | 29.78082 | 1 | 3.724108 | 1.70286 | 0.920052 | 4.879736 | 0.847488 | 0.479716 | 2.165596 | 3.097866 | 7.466493 | 5.475918 | 0.542419 | 0.941768 | 0.300012 | 0.609879 | 8.907748 |
| TCGA-EE-A29C | 6.580822 | 1 | 5.370323 | 2.843257 | 3.311534 | 5.705264 | 2.050012 | 2.200084 | 2.79762 | 3.257402 | 7.809954 | 5.055547 | 1.223572 | 2.394356 | 2.76066 | 1.270101 | 8.965263 |
| TCGA-EB-A3XF | 0.761644 | 0 | 8.773145 | 3.056513 | 4.177921 | 5.435062 | 8.549656 | 6.183425 | 4.414015 | 3.716894 | 7.21417 | 7.466493 | 2.083509 | 5.074088 | 3.163252 | 0.733082 | 9.072918 |
| TCGA-ER-A193 | 2.616438 | 1 | 8.99563 | 3.735173 | 7.336058 | 5.757281 | 6.936064 | 8.279393 | 4.432535 | 3.909344 | 6.327438 | 6.955331 | 2.286747 | 3.828144 | 2.793969 | 1.114159 | 9.320966 |
| TCGA-EE-A3AC | 5.336986 | 0 | 7.331705 | 3.761692 | 5.872205 | 5.42125 | 6.747052 | 6.610932 | 4.920814 | 4.267482 | 7.524388 | 6.466031 | 2.068934 | 3.229175 | 2.740648 | 1.236345 | 8.033017 |
| TCGA-EE-A2GB | 4.939726 | 0 | 8.589809 | 2.750789 | 3.359029 | 4.742023 | 4.566639 | 4.696304 | 3.169022 | 3.375188 | 7.776094 | 6.10055 | 0.675821 | 3.646462 | 2.00075 | 0.547218 | 6.674014 |
| TCGA-GN-A4U9 | 1.843836 | 1 | 7.451022 | 2.049259 | 3.194064 | 4.732833 | 4.34151 | 5.176056 | 3.434209 | 3.175691 | 5.966649 | 2.572875 | 1.215818 | 0.455931 | 1.433552 | 0.09833 | 7.406944 |
| TCGA-EB-A6QY | 1.046575 | 0 | 3.107591 | 1.86598 | 2.806443 | 3.139319 | 2.522833 | 2.776986 | 1.543139 | 3.222127 | 6.163384 | 4.815384 | 3.455991 | 2.977539 | 0.903816 | 1.649254 | 7.649042 |
| TCGA-BF-A1PV | 0.038356 | 0 | 7.541294 | 1.787918 | 1.609979 | 3.833367 | 1.584053 | 1.073383 | 1.74055 | 3.530726 | 8.084116 | 5.712048 | 0.631193 | 4.207975 | 1.052444 | 0.712832 | 7.557357 |
| TCGA-BF-AAP0 | 1.243836 | 0 | 7.769483 | 3.812917 | 7.803093 | 6.671517 | 7.066337 | 7.15767 | 4.447051 | 3.274313 | 7.322356 | 6.901181 | 3.455991 | 4.200025 | 5.533503 | 0.827322 | 10.09768 |
| TCGA-DA-A1IA | 5.493151 | 1 | 7.344869 | 2.272241 | 4.019344 | 3.867095 | 0.631121 | 1.308856 | 1.249942 | 2.268334 | 7.282239 | 6.066519 | 0.457919 | 4.217774 | 3.542669 | 0.639598 | 8.643297 |
| TCGA-D3-A2JP | 4.964384 | 0 | 7.222808 | 3.535575 | 6.418429 | 7.308973 | 5.737357 | 6.367035 | 3.911231 | 2.887996 | 7.578904 | 6.153608 | 2.653866 | 4.290962 | 2.916473 | 0.635064 | 11.02135 |
| TCGA-EB-A5VV | 0.586301 | 0 | 6.99612 | 2.926174 | 7.974782 | 5.646451 | 6.451344 | 7.39195 | 5.813069 | 3.08113 | 6.528434 | 5.490445 | 4.80049 | 3.214798 | 8.681574 | 0.524313 | 7.567918 |
| TCGA-D3-A5GL | 10.48219 | 0 | 3.656786 | 2.692278 | 3.199867 | 4.400336 | 1.573226 | 1.43241 | 1.576893 | 3.083347 | 8.359438 | 5.423995 | 1.103748 | 3.595504 | 0.540987 | 0.871436 | 9.352444 |
| TCGA-EB-A3XD | 3.178082 | 0 | 6.143819 | 2.92143 | 6.074119 | 6.030441 | 3.41456 | 6.255887 | 3.314724 | 2.144283 | 7.277861 | 4.832216 | 4.863402 | 2.76066 | 3.318408 | 1.943702 | 6.895262 |
| TCGA-GN-A8LL | 1.780822 | 1 | 9.289949 | 1.662647 | 4.135215 | 4.19164 | 0.945016 | 1.755014 | 0.570262 | 3.462447 | 7.387576 | 3.397084 | 0.634948 | 4.999821 | 1.042284 | 0.228444 | 7.218327 |
| TCGA-FS-A1ZJ | 3.947945 | 1 | 7.991229 | 2.126924 | 4.351853 | 4.864593 | 4.009238 | 4.749759 | 2.528319 | 3.304135 | 6.926438 | 3.369934 | 1.51549 | 2.316872 | 2.299383 | 0.569086 | 7.567918 |
| TCGA-W3-A828 | 10.09041 | 1 | 5.199953 | 3.175368 | 4.179202 | 6.05292 | 2.932683 | 3.546793 | 4.041793 | 3.332211 | 6.252268 | 5.155553 | 1.704596 | 1.471065 | 1.885549 | 0.716844 | 9.818344 |
| TCGA-EE-A2MQ | 3.60274 | 1 | 8.395557 | 4.198719 | 3.855939 | 3.949609 | 4.015587 | 4.305483 | 2.965733 | 3.801161 | 6.045474 | 5.642036 | 1.595374 | 3.423796 | 1.91533 | 0.765458 | 10.45413 |
| TCGA-EE-A2MH | 1.413699 | 1 | 9.742234 | 2.855212 | 5.92828 | 6.122744 | 4.861639 | 4.862238 | 3.861163 | 3.372566 | 6.365155 | 6.027505 | 2.416555 | 4.154915 | 5.420319 | 0.568292 | 9.634608 |
| TCGA-D3-A1Q9 | 2.632877 | 1 | 5.404089 | 2.954843 | 6.204989 | 5.843018 | 4.751935 | 4.108692 | 3.77005 | 3.606001 | 7.125979 | 5.499133 | 1.452821 | 3.985358 | 2.011259 | 1.287408 | 9.289949 |
| TCGA-DA-A1IB | 3.383562 | 1 | 7.317853 | 4.573066 | 7.919211 | 7.243384 | 6.699641 | 8.746058 | 6.201606 | 2.96045 | 8.128757 | 6.569226 | 4.58463 | 3.720473 | 6.90424 | 1.038483 | 9.598808 |
| TCGA-EE-A2MN | 3.961644 | 1 | 8.312242 | 4.429691 | 6.108429 | 6.495597 | 5.435062 | 6.186645 | 4.973876 | 3.238297 | 6.736535 | 6.656557 | 2.541895 | 5.016435 | 3.395085 | 1.455039 | 7.831019 |
| TCGA-D3-A1Q6 | 5.983562 | 1 | 3.495282 | 4.44796 | 3.342736 | 4.465634 | 4.461937 | 2.1464 | 3.233094 | 4.180524 | 7.643215 | 3.826832 | 0.821446 | 3.294806 | 2.024443 | 1.662073 | 11.329 |
| TCGA-D3-A8GE | 2.20274 | 0 | 6.840305 | 4.786218 | 5.730404 | 6.606234 | 7.226721 | 6.424496 | 4.582052 | 3.379914 | 5.788227 | 4.562647 | 2.142158 | 2.960774 | 2.803713 | 2.114331 | 11.16651 |
| TCGA-EB-A24D | 1.767123 | 0 | 5.750221 | 1.914737 | 4.297535 | 3.506803 | 4.064035 | 5.55478 | 2.565645 | 2.957028 | 6.314155 | 4.527444 | 1.953308 | 4.973876 | 2.222646 | 0.660303 | 6.297491 |
| TCGA-DA-A1I1 | 18.54247 | 0 | 8.865828 | 4.031325 | 6.108429 | 6.982079 | 7.295768 | 7.426424 | 5.294618 | 3.767833 | 6.97555 | 7.853397 | 2.118826 | 4.767066 | 3.310289 | 1.206862 | 9.780437 |
| TCGA-D3-A1Q1 | 1.380822 | 1 | 4.003276 | 2.180613 | 2.395237 | 5.286427 | 1.727513 | 1.392248 | 2.752915 | 3.499383 | 7.966601 | 5.747916 | 0.754631 | 1.160472 | 0.498646 | 0.311617 | 7.416572 |
| TCGA-XV-A9W5 | 1.073973 | 0 | 8.066661 | 1.971782 | 4.561693 | 5.508969 | 4.171608 | 4.399389 | 2.566247 | 2.764005 | 6.916855 | 4.891831 | 1.994437 | 4.303257 | 1.611127 | 0.722974 | 11.71852 |
| TCGA-ER-A198 | 4.230137 | 1 | 8.980385 | 3.917367 | 5.448754 | 5.634483 | 5.526426 | 4.975125 | 4.674527 | 4.248628 | 7.445703 | 6.145504 | 1.735593 | 4.072103 | 2.315031 | 1.090383 | 7.942817 |
| TCGA-D3-A51F | 4.643836 | 0 | 9.513137 | 5.491363 | 10.15165 | 9.415135 | 7.336058 | 7.737162 | 6.321701 | 3.302893 | 7.982479 | 7.344869 | 5.040607 | 4.751386 | 4.679308 | 1.788456 | 10.96016 |
| TCGA-D3-A1QB | 7.978082 | 0 | 9.336361 | 3.879906 | 8.48398 | 7.541294 | 8.773145 | 7.776094 | 4.584118 | 4.009642 | 6.744385 | 7.313439 | 3.153629 | 4.575057 | 4.559297 | 1.434874 | 7.503515 |
| TCGA-EE-A2GS | 6.767123 | 1 | 9.196354 | 2.986316 | 5.33779 | 4.914661 | 6.557743 | 5.621706 | 3.551655 | 4.523475 | 9.513137 | 5.178268 | 1.202026 | 3.762035 | 3.36065 | 0.394086 | 8.936502 |
| TCGA-EE-A2A5 | 3.273973 | 1 | 8.509861 | 3.164196 | 2.519863 | 5.463601 | 2.583731 | 2.134387 | 3.260939 | 3.351401 | 5.739733 | 4.978943 | 1.045026 | 2.941451 | 1.689705 | 0.507581 | 8.323727 |
| TCGA-FS-A1Z3 | 1.742466 | 1 | 4.102966 | 2.211842 | 2.431662 | 4.754117 | 2.179416 | 3.643679 | 2.859434 | 3.603899 | 6.733851 | 4.929516 | 0.631193 | 3.896746 | 1.980412 | 0.393806 | 7.436078 |
| TCGA-WE-A8ZM | 8.443836 | 0 | 4.727299 | 1.294294 | 0.697911 | 3.944943 | 0.148348 | 0.097462 | 0.907388 | 3.230161 | 6.885774 | 5.118374 | 0.178615 | 3.356404 | 1.596004 | 0.428769 | 10.09768 |
| TCGA-FR-A69P | 1.309589 | 0 | 4.240521 | 2.555739 | 3.26323 | 5.429472 | 2.518959 | 3.624704 | 2.69563 | 3.065868 | 6.965222 | 5.583045 | 0.811312 | 1.498363 | 1.492765 | 0.429599 | 7.927317 |
| TCGA-Z2-AA3S | 8.082192 | 0 | 9.057216 | 2.498995 | 4.238782 | 5.171486 | 2.685817 | 3.420131 | 3.204104 | 3.840427 | 5.882708 | 4.072502 | 1.577751 | 3.650398 | 2.132896 | 0.387409 | 5.985265 |
| TCGA-D3-A3MU | 3.312329 | 0 | 8.458672 | 3.068983 | 4.476155 | 5.042584 | 4.008076 | 4.529994 | 3.445652 | 2.756889 | 7.711737 | 6.472349 | 1.512419 | 3.804089 | 2.856139 | 0.872945 | 9.598808 |
| TCGA-EB-A5SH | 4.50137 | 0 | 4.484661 | 1.835278 | 4.326258 | 4.949731 | 2.917088 | 3.619315 | 2.155984 | 2.558081 | 8.603012 | 4.38548 | 1.102525 | 3.698835 | 4.024614 | 0.878205 | 6.453496 |
| TCGA-FW-A3R5 | 3.079452 | 0 | 4.676105 | 3.161333 | 3.981122 | 3.52627 | 4.715788 | 5.932236 | 3.21513 | 3.165489 | 7.137556 | 4.918986 | 1.668143 | 1.838783 | 3.04264 | 1.283205 | 8.643297 |
| TCGA-D3-A8GB | 2.569863 | 1 | 7.349502 | 3.033923 | 7.654825 | 6.255887 | 6.913876 | 5.905183 | 3.932335 | 3.43762 | 8.041226 | 4.65763 | 2.875247 | 4.887011 | 4.933278 | 0.492914 | 9.818344 |
| TCGA-GN-A263 | 1.279452 | 1 | 4.13645 | 3.518381 | 4.353639 | 3.550602 | 4.355401 | 4.331117 | 3.40904 | 4.35629 | 7.942817 | 5.054229 | 1.896045 | 3.630672 | 3.359346 | 0.084459 | 7.83865 |
| TCGA-FW-A3TV | 1.126027 | 0 | 4.287455 | 3.65926 | 3.938824 | 6.034935 | 2.917088 | 4.061627 | 3.270421 | 3.566779 | 7.889773 | 5.202155 | 2.019641 | 3.877619 | 3.464133 | 1.412319 | 9.497298 |
| TCGA-EE-A2MU | 4.438356 | 0 | 9.320966 | 4.537419 | 7.508591 | 7.145736 | 6.59212 | 7.578904 | 4.97831 | 3.313126 | 7.397015 | 6.576047 | 2.570082 | 4.666072 | 4.254264 | 1.322855 | 8.99563 |
| TCGA-EE-A20C | 12.60548 | 1 | 8.81294 | 2.497156 | 1.520355 | 4.770358 | 1.20709 | 0.392215 | 2.322251 | 3.984195 | 4.285255 | 5.338646 | 1.013487 | 3.665282 | 1.261667 | 0.655491 | 10.58227 |
| TCGA-FS-A4F2 | 4.178082 | 1 | 7.846293 | 3.819962 | 1.409597 | 7.269034 | 1.253939 | 0.591827 | 3.771892 | 3.276597 | 6.585266 | 6.659034 | 1.301353 | 2.738853 | 0.911162 | 0.689511 | 8.852298 |
| TCGA-EB-A42Z | 1.208219 | 0 | 6.819979 | 2.766804 | 4.292719 | 5.279968 | 3.693003 | 4.138497 | 2.478626 | 3.047097 | 7.853397 | 5.256652 | 1.157033 | 4.405585 | 0.891874 | 2.528319 | 10.49427 |
| TCGA-D3-A51R | 5.317808 | 0 | 3.416058 | 0.22313 | 0.519321 | 2.414184 | 0.497003 | 0.244968 | 0.519202 | 2.654748 | 3.391824 | 2.873389 | 0.081651 | 0.18447 | 0.191979 | 0.06254 | 7.209836 |
| TCGA-EE-A29R | 1.205479 | 0 | 7.809954 | 1.875555 | 2.48358 | 4.398458 | 1.872985 | 2.787164 | 1.761818 | 2.78373 | 6.889013 | 5.349734 | 0.535422 | 2.846321 | 1.561286 | 0.466403 | 10.41592 |
| TCGA-EE-A2GL | 6.638356 | 0 | 7.860614 | 2.363438 | 5.949985 | 4.874369 | 3.801161 | 5.494194 | 4.83519 | 3.087146 | 7.950456 | 6.293738 | 2.104014 | 3.968271 | 6.557743 | 0.422581 | 8.70773 |
| TCGA-DA-A1I0 | 1.69863 | 1 | 4.713641 | 2.879861 | 4.353189 | 5.616495 | 1.154228 | 2.872444 | 3.054944 | 2.211842 | 7.625453 | 5.225149 | 1.564921 | 2.497466 | 4.550848 | 0.60162 | 11.24696 |
| TCGA-D9-A148 | 12.6274 | 0 | 8.024331 | 2.491017 | 5.081679 | 4.456385 | 4.19164 | 5.024398 | 3.023733 | 3.234068 | 5.124828 | 5.758424 | 2.09529 | 4.086252 | 1.975622 | 0.817383 | 7.235035 |
| TCGA-D3-A51K | 2.745205 | 0 | 8.643297 | 2.811996 | 5.521516 | 6.424496 | 3.669552 | 3.191831 | 2.58188 | 3.440983 | 7.756158 | 4.656576 | 2.008879 | 4.732246 | 3.011804 | 0.318942 | 7.059001 |
| TCGA-EE-A3J3 | 14.34795 | 1 | 9.688332 | 3.285706 | 5.460785 | 5.36265 | 5.125551 | 4.910318 | 3.784622 | 4.075338 | 8.312242 | 5.210583 | 1.719291 | 4.680326 | 2.766471 | 0.528388 | 9.651786 |
| TCGA-FS-A1ZK | 1.994521 | 1 | 6.656557 | 1.35928 | 1.516673 | 3.50481 | 1.309898 | 0.954257 | 1.718362 | 3.025961 | 7.009619 | 5.851858 | 0.343764 | 3.15655 | 0.891661 | 0.637959 | 7.173327 |
| TCGA-BF-A3DM | 1.646575 | 0 | 10.58227 | 2.294554 | 3.618256 | 5.368573 | 4.499939 | 1.500604 | 2.053206 | 4.247337 | 8.289803 | 6.926438 | 0.528387 | 4.786798 | 3.004961 | 0.429792 | 9.336361 |
| TCGA-GF-A6C8 | 0.169863 | 0 | 9.103504 | 3.016177 | 5.263841 | 5.749125 | 6.481044 | 4.688829 | 3.460063 | 4.114697 | 7.317853 | 6.560032 | 1.15206 | 4.603888 | 3.914651 | 0.865928 | 9.818344 |
| TCGA-EE-A2M8 | 1.646575 | 1 | 7.882242 | 5.633394 | 9.530744 | 8.395557 | 8.323727 | 8.907748 | 6.537258 | 3.854821 | 6.955331 | 6.453496 | 4.095214 | 4.12453 | 5.175275 | 1.716034 | 9.688332 |
| TCGA-D3-A3CF | 2.043836 | 1 | 6.517303 | 4.15118 | 5.143815 | 6.596799 | 5.621706 | 5.61436 | 5.052827 | 3.922373 | 8.334715 | 4.972583 | 2.954526 | 4.047659 | 5.882708 | 1.359833 | 9.48095 |
| TCGA-ER-A19G | 25.1726 | 0 | 8.907748 | 3.490516 | 6.608574 | 6.867298 | 8.89291 | 6.502157 | 5.538602 | 4.51957 | 6.211944 | 8.323727 | 2.230488 | 5.006465 | 3.632453 | 1.270605 | 9.119603 |
| TCGA-BF-A1PU | 1.060274 | 0 | 3.038554 | 1.092723 | 1.955699 | 4.376264 | 0.205649 | 0.853155 | 0.807709 | 2.286747 | 9.226197 | 5.837889 | 0.600149 | 4.181357 | 1.746405 | 0.899156 | 9.530744 |
| TCGA-FR-A3YN | 7.747945 | 0 | 8.196775 | 3.16388 | 5.702981 | 5.583045 | 5.008452 | 5.467385 | 3.444633 | 3.956194 | 6.702211 | 6.424496 | 1.621206 | 4.078226 | 3.396092 | 0.717292 | 9.799361 |
| TCGA-EB-A5VU | 0.879452 | 1 | 3.711778 | 2.585303 | 2.527381 | 6.05292 | 3.021232 | 2.552372 | 3.591376 | 3.129058 | 8.334715 | 6.474385 | 2.41627 | 2.853981 | 0.949672 | 0.945952 | 8.216852 |
| TCGA-D3-A2J9 | 1.980822 | 1 | 8.421176 | 4.194116 | 9.211383 | 7.308973 | 7.737162 | 9.415135 | 5.773992 | 3.283737 | 6.907455 | 7.185193 | 4.672972 | 4.274326 | 6.106906 | 1.402776 | 9.924688 |
| TCGA-EB-A4XL | 2.128767 | 0 | 7.711737 | 1.641213 | 4.841666 | 4.815384 | 6.768916 | 6.537258 | 2.816068 | 3.43455 | 6.696849 | 5.979426 | 3.990072 | 4.715788 | 1.926459 | 0.853155 | 9.448603 |
| TCGA-BF-AAP8 | 1.224658 | 0 | 7.359111 | 1.981597 | 4.146633 | 5.498116 | 2.423795 | 2.321964 | 1.971492 | 3.567468 | 6.651794 | 3.417426 | 1.423333 | 3.072703 | 0.712267 | 0.239854 | 9.13588 |
| TCGA-D3-A3MO | 0.778082 | 1 | 6.81436 | 1.913201 | 1.638004 | 5.424907 | 0.222223 | 0.138608 | 1.663221 | 2.879549 | 6.578319 | 5.757281 | 0.640044 | 2.775106 | 1.349426 | 0.388331 | 12.2573 |
| TCGA-EE-A2GH | 18.35342 | 0 | 6.72588 | 4.484661 | 8.268444 | 7.062695 | 6.840305 | 8.289803 | 5.00579 | 2.543076 | 7.649042 | 5.134252 | 3.955781 | 3.623975 | 3.546793 | 0.649346 | 9.818344 |
| TCGA-FS-A1Z0 | 16.88767 | 1 | 8.206697 | 3.967864 | 5.882708 | 6.338468 | 7.322356 | 8.346721 | 5.352249 | 3.726952 | 6.837192 | 5.656405 | 2.864743 | 3.818891 | 3.232295 | 1.246411 | 7.698557 |
| TCGA-EB-A5SG | 5.687671 | 0 | 7.966601 | 4.819439 | 8.839039 | 7.291223 | 8.433625 | 9.305759 | 5.766811 | 2.943602 | 7.059001 | 6.707448 | 4.482758 | 5.303511 | 4.15533 | 1.843642 | 9.564239 |
| TCGA-EE-A2GP | 1.158904 | 1 | 8.48398 | 3.910443 | 3.752834 | 6.550894 | 4.141326 | 4.270023 | 4.580092 | 3.802662 | 6.10055 | 6.132505 | 1.947972 | 3.499719 | 2.338478 | 0.81223 | 9.993233 |
| TCGA-EE-A29V | 2.156164 | 1 | 3.655388 | 1.415816 | 2.612852 | 4.074919 | 0.65744 | 1.101359 | 1.168326 | 2.693194 | 8.370865 | 5.873481 | 0.841786 | 3.703908 | 1.965555 | 0.887177 | 8.167888 |
| TCGA-LH-A9QB | 30.73151 | 0 | 7.562478 | 1.715735 | 3.476386 | 4.009642 | 2.92678 | 1.738527 | 1.793202 | 3.284049 | 5.243096 | 2.560829 | 0.619639 | 2.01185 | 2.340269 | 0.283052 | 11.41333 |
| TCGA-W3-A825 | 5.252055 | 1 | 6.610932 | 3.673162 | 5.933692 | 6.982079 | 5.62799 | 6.218834 | 4.225862 | 2.796999 | 6.138973 | 6.194984 | 2.087168 | 1.303421 | 2.467108 | 0.725989 | 10.01891 |
| TCGA-EB-A3Y6 | 0.345205 | 0 | 7.081368 | 2.886473 | 4.560256 | 5.789443 | 5.091615 | 5.380945 | 2.82252 | 3.693003 | 6.99612 | 5.610215 | 1.223326 | 4.775981 | 1.598286 | 0.69122 | 8.773145 |
| TCGA-EE-A183 | 2.241096 | 1 | 7.567918 | 3.327608 | 5.346306 | 6.040997 | 2.515294 | 3.858933 | 3.73013 | 2.611045 | 7.698557 | 6.60147 | 2.098302 | 3.357041 | 5.552687 | 1.069372 | 8.89291 |
| TCGA-EB-A4P0 | 0.893151 | 1 | 7.044609 | 2.40683 | 4.527956 | 5.518588 | 3.678741 | 3.699917 | 2.100112 | 2.845418 | 8.226828 | 4.159938 | 1.002951 | 5.502088 | 0.935285 | 0.076224 | 9.289949 |
| TCGA-ER-A19M | 5.087671 | 1 | 9.530744 | 4.211375 | 7.066337 | 6.342324 | 8.408325 | 8.128757 | 4.965549 | 3.299967 | 7.062695 | 7.226721 | 2.850921 | 5.132797 | 2.374585 | 1.19987 | 8.395557 |
| TCGA-GF-A2C7 | 0.057534 | 0 | 4.9041 | 1.551302 | 3.088685 | 4.47279 | 0.395466 | 0.611922 | 2.005275 | 3.034851 | 7.260362 | 5.451578 | 1.755919 | 3.974507 | 1.006222 | 0.740716 | 8.421176 |
| TCGA-EE-A2MS | 13.53973 | 0 | 5.635556 | 3.084938 | 5.832894 | 6.594455 | 3.85708 | 4.183922 | 4.10949 | 3.254753 | 5.489424 | 6.310444 | 1.826336 | 2.824697 | 1.7926 | 0.592181 | 6.562253 |
| TCGA-EE-A3JB | 16.81644 | 0 | 8.852298 | 4.851699 | 4.39425 | 7.03403 | 5.645365 | 4.304572 | 5.924147 | 4.538894 | 6.284413 | 6.029007 | 1.963739 | 2.368575 | 2.208246 | 1.131835 | 10.24322 |
| TCGA-DA-A95Z | 1.084932 | 0 | 8.99563 | 2.418699 | 3.914275 | 4.536409 | 3.773567 | 4.565143 | 3.10381 | 2.745878 | 7.889773 | 6.171626 | 1.625443 | 3.236682 | 1.186752 | 0.858334 | 7.803093 |
| TCGA-D9-A4Z5 | 0.59726 | 0 | 6.94248 | 3.850337 | 2.82927 | 6.749759 | 2.774508 | 2.140053 | 3.738033 | 3.776602 | 7.950456 | 7.363574 | 1.489117 | 4.961771 | 1.477551 | 0.761824 | 9.706195 |
| TCGA-D3-A3CB | 13.87671 | 0 | 8.216852 | 5.022443 | 7.889773 | 7.466493 | 5.983812 | 6.587577 | 6.663957 | 3.965917 | 6.546306 | 6.694161 | 3.056838 | 4.188598 | 5.52742 | 1.980412 | 9.241958 |
| TCGA-BF-A1PX | 0.772603 | 1 | 8.523571 | 3.74887 | 8.049629 | 6.97555 | 5.895766 | 6.474385 | 3.892126 | 2.500541 | 7.999387 | 5.783432 | 4.058764 | 4.58616 | 3.401447 | 1.160929 | 9.415135 |
| TCGA-EE-A2GU | 7.90137 | 0 | 8.669115 | 3.64968 | 6.578319 | 5.252584 | 5.894443 | 7.567918 | 4.653893 | 4.341988 | 6.380644 | 6.926438 | 2.04054 | 4.692022 | 3.618256 | 1.246921 | 6.608574 |
| TCGA-D9-A6E9 | 0.824658 | 0 | 8.865828 | 4.720708 | 8.839039 | 8.084116 | 5.671445 | 6.741728 | 5.206002 | 2.857023 | 9.15014 | 7.269034 | 3.152682 | 4.774835 | 4.302827 | 2.035413 | 8.617038 |
| TCGA-GN-A4U8 | 4.073973 | 0 | 9.010101 | 3.823665 | 7.066337 | 6.895262 | 6.97555 | 7.141812 | 4.591325 | 3.580918 | 7.436078 | 5.810606 | 3.10473 | 4.866415 | 4.821765 | 0.456056 | 9.057216 |
| TCGA-DA-A3F2 | 2.827397 | 1 | 7.717973 | 5.930877 | 9.211383 | 8.70773 | 3.540993 | 5.478675 | 4.913387 | 2.006476 | 7.336058 | 5.412103 | 4.204152 | 3.396439 | 4.713121 | 2.251149 | 8.247378 |
| TCGA-FS-A4FB | 2.227397 | 1 | 9.634608 | 1.319119 | 2.281864 | 3.941502 | 0.691522 | 1.44057 | 1.433219 | 3.044881 | 7.197699 | 4.815384 | 0.675821 | 3.307686 | 2.539189 | 0.523567 | 9.226197 |
| TCGA-EB-A5FP | 1.243836 | 1 | 6.526134 | 1.288138 | 1.900096 | 2.050168 | 1.574073 | 1.985482 | 0.571435 | 3.195048 | 7.041155 | 4.701131 | 1.441788 | 4.050812 | 0.339906 | 0.531084 | 8.138531 |
| TCGA-D3-A8GK | 14.18356 | 0 | 4.636821 | 1.530358 | 2.552372 | 3.374235 | 0.244702 | 1.098798 | 0.722637 | 3.138316 | 8.007828 | 5.28474 | 0.196001 | 2.959836 | 0.596858 | 0.782784 | 7.020203 |
| TCGA-EB-A44R | 0.863014 | 1 | 8.471285 | 2.500219 | 6.404654 | 5.806795 | 3.282451 | 4.80617 | 3.084938 | 3.250568 | 7.044609 | 4.624495 | 1.25539 | 4.150773 | 2.021168 | 0.497918 | 7.551771 |
| TCGA-EE-A2M5 | 1.805479 | 1 | 5.210583 | 2.862882 | 5.455243 | 5.526426 | 5.187191 | 5.52742 | 3.629633 | 3.529338 | 8.936502 | 3.187066 | 1.249942 | 1.565794 | 3.109808 | 1.447775 | 11.16651 |
| TCGA-ER-A2NB | 2.347945 | 1 | 7.16174 | 2.42024 | 6.97863 | 5.880105 | 3.010243 | 3.745606 | 2.82587 | 2.150251 | 7.47166 | 5.053549 | 4.349173 | 4.037405 | 2.174299 | 2.255063 | 8.408325 |
| TCGA-D9-A3Z4 | 1.421918 | 1 | 9.947093 | 3.173448 | 5.788227 | 5.784634 | 6.286264 | 6.231112 | 4.293578 | 4.336935 | 6.895262 | 5.691634 | 1.51549 | 4.884012 | 2.928639 | 0.579582 | 9.780437 |
| TCGA-EE-A2GD | 28.34521 | 1 | 7.860614 | 5.142362 | 3.874933 | 7.503515 | 2.991559 | 2.22929 | 5.301885 | 3.163252 | 7.327055 | 7.107174 | 1.754104 | 3.071452 | 3.67101 | 1.318637 | 10.58227 |
| TCGA-GN-A4U7 | 0.868493 | 1 | 4.103801 | 0.98106 | 2.056783 | 3.37653 | 0.579139 | 0.814616 | 0.621784 | 2.973794 | 7.562478 | 4.193273 | 0.298854 | 2.983166 | 0.975279 | 0.472396 | 9.041563 |
| TCGA-EE-A182 | 1.224658 | 1 | 4.920814 | 3.153957 | 5.099338 | 6.779902 | 1.794983 | 2.618569 | 2.961364 | 1.994148 | 9.398771 | 5.355607 | 1.490778 | 3.273996 | 2.026847 | 1.162874 | 9.289949 |
| TCGA-GN-A8LN | 2.115068 | 0 | 6.186645 | 3.133502 | 2.779123 | 7.16174 | 5.772846 | 3.486192 | 3.697354 | 3.031168 | 6.649415 | 7.125979 | 1.445924 | 3.876069 | 1.390118 | 0.273394 | 10.09768 |
| TCGA-W3-AA21 | 8.753425 | 1 | 6.189925 | 0.986863 | 4.192874 | 3.194381 | 4.265761 | 2.887719 | 1.245618 | 2.642354 | 7.673388 | 6.147116 | 0.703213 | 4.755814 | 3.125338 | 0.289278 | 9.724841 |
| TCGA-EE-A29B | 7.090411 | 1 | 8.041226 | 3.442993 | 4.140516 | 5.670395 | 2.232552 | 2.238862 | 3.348831 | 3.232132 | 8.226828 | 5.430341 | 1.184449 | 4.568586 | 1.301353 | 0.702753 | 7.277861 |
| TCGA-D3-A3CE | 5.019178 | 1 | 5.58502 | 3.68983 | 6.589788 | 7.247635 | 6.758168 | 7.18152 | 5.132797 | 2.981614 | 6.515039 | 6.684046 | 2.09046 | 2.913103 | 3.324644 | 0.789768 | 8.300745 |
| TCGA-EE-A20I | 1.128767 | 1 | 7.596001 | 2.610133 | 4.968153 | 4.756384 | 4.003276 | 5.065059 | 2.998695 | 3.595859 | 7.942817 | 6.58054 | 1.277247 | 4.497974 | 2.5596 | 1.080467 | 6.965222 |
| TCGA-D3-A2JE | 2.30411 | 1 | 7.304577 | 1.15206 | 2.51375 | 3.249943 | 2.54434 | 3.026274 | 1.520355 | 2.91372 | 6.923118 | 4.301509 | 0.881743 | 0.611628 | 1.511837 | 0.817383 | 7.974782 |
| TCGA-EE-A3AB | 10.2274 | 0 | 8.236801 | 5.050004 | 4.518128 | 8.024331 | 2.914014 | 2.646626 | 5.549713 | 2.948027 | 5.784634 | 6.848972 | 2.91372 | 1.437382 | 1.949425 | 1.197419 | 11.24696 |
| TCGA-D3-A8GR | 10.80274 | 1 | 8.733064 | 2.279432 | 3.44364 | 5.388906 | 1.468316 | 0.821284 | 2.087456 | 3.42819 | 8.746058 | 4.133171 | 1.924331 | 3.469204 | 0.857534 | 0.311617 | 8.408325 |
| TCGA-W3-A824 | 19.0137 | 0 | 9.780437 | 3.531053 | 7.0306 | 5.630144 | 7.137556 | 5.800543 | 3.667404 | 3.851057 | 8.549656 | 6.449298 | 1.775727 | 5.417604 | 2.842345 | 0.961942 | 8.907748 |
| TCGA-DA-A95Y | 1.178082 | 1 | 7.006306 | 2.504757 | 2.418075 | 4.240088 | 1.443641 | 1.422523 | 1.596844 | 3.320022 | 6.98562 | 4.379495 | 1.062172 | 3.212863 | 2.253256 | 1.437382 | 9.057216 |
| TCGA-GN-A4U3 | 10.1589 | 0 | 8.458672 | 2.8675 | 2.819407 | 5.335305 | 1.725076 | 1.649254 | 2.087456 | 3.425431 | 6.474385 | 7.173327 | 1.005102 | 4.88759 | 0.666114 | 0.370021 | 8.839039 |
| TCGA-ER-A196 | 4.890411 | 0 | 4.541897 | 2.00766 | 5.023727 | 4.269595 | 2.357953 | 1.918607 | 2.19289 | 3.199867 | 6.641932 | 5.306915 | 2.245159 | 2.67715 | 2.06496 | 1.606853 | 10.01891 |
| TCGA-DA-A95W | 3.112329 | 0 | 9.43193 | 2.36128 | 5.435062 | 5.624821 | 4.152401 | 4.369433 | 2.534703 | 3.238635 | 6.461754 | 5.634483 | 1.634008 | 5.03317 | 1.802638 | 0.757409 | 7.660811 |
| TCGA-WE-A8ZR | 0.750685 | 1 | 6.534983 | 2.407146 | 3.678394 | 4.674527 | 2.705972 | 2.777889 | 2.46291 | 3.389878 | 8.334715 | 4.479456 | 1.075193 | 4.565143 | 2.768638 | 0.397938 | 7.950456 |
| TCGA-WE-AAA4 | 2.082192 | 0 | 8.936502 | 4.705483 | 9.398771 | 8.268444 | 7.048227 | 7.15372 | 5.162736 | 2.700473 | 9.634608 | 8.206697 | 2.518655 | 3.965917 | 4.518128 | 1.802312 | 10.12502 |
| TCGA-BF-AAOX | 1.216438 | 0 | 6.681398 | 0.607069 | 2.825304 | 3.37653 | 1.744657 | 1.880193 | 1.091104 | 2.809837 | 8.334715 | 4.383654 | 1.261175 | 2.865304 | 1.852261 | 1.266818 | 7.295768 |
| TCGA-DA-A1I8 | 4.493151 | 1 | 9.305759 | 3.835565 | 2.851534 | 7.904414 | 2.547946 | 1.171284 | 4.117993 | 3.507495 | 6.718114 | 7.39195 | 1.362683 | 3.815145 | 3.625383 | 1.382257 | 11.41333 |
| TCGA-EE-A3J5 | 3.079452 | 1 | 5.536532 | 3.971032 | 6.122744 | 5.943017 | 5.733907 | 6.715439 | 4.194116 | 3.108831 | 7.471659 | 5.790685 | 2.464447 | 2.806443 | 3.786873 | 1.14599 | 8.773145 |
| TCGA-XV-A9W2 | 1.142466 | 0 | 6.481044 | 1.079573 | 2.834595 | 4.691482 | 5.914582 | 4.969428 | 1.575824 | 2.995263 | 7.79637 | 3.941502 | 3.482835 | 4.460073 | 1.142906 | 1.117255 | 8.300745 |
| TCGA-BF-A3DJ | 1.271233 | 0 | 9.598808 | 4.542402 | 4.921421 | 7.304577 | 5.111846 | 3.917725 | 4.621883 | 3.935728 | 7.239183 | 7.53005 | 2.038687 | 4.220707 | 2.825582 | 0.899785 | 10.34278 |
| TCGA-BF-AAP2 | 1.109589 | 0 | 6.834409 | 2.115229 | 4.404437 | 5.442368 | 4.494178 | 4.378123 | 2.45353 | 3.690538 | 7.476715 | 5.278293 | 1.846649 | 4.577561 | 2.824996 | 1.786106 | 7.421277 |
| TCGA-EE-A3AA | 10.3589 | 0 | 7.095916 | 3.494259 | 5.456162 | 5.301885 | 5.995377 | 6.227571 | 4.582052 | 4.31166 | 7.860614 | 7.226721 | 2.289139 | 4.295766 | 5.258275 | 1.326482 | 10.09768 |
| TCGA-D3-A2JG | 9.460274 | 1 | 5.287281 | 1.754104 | 3.747061 | 4.169137 | 2.685518 | 2.652311 | 2.335454 | 3.277559 | 6.739135 | 6.098913 | 0.90038 | 3.156861 | 1.503668 | 0.572542 | 8.458672 |
| TCGA-GN-A8LK | 4.175342 | 1 | 3.562987 | 0.597239 | 2.434681 | 2.918624 | 0.440544 | 0.608887 | 0.285683 | 2.846931 | 8.177475 | 4.722353 | 0.232702 | 2.370335 | 0.42268 | 0.758841 | 6.99612 |
| TCGA-EE-A2MT | 5.934247 | 0 | 6.331165 | 2.193797 | 4.023035 | 3.643679 | 3.147012 | 3.864515 | 1.594267 | 3.435252 | 8.236801 | 7.081368 | 0.52732 | 2.272845 | 1.661504 | 0.611476 | 9.211383 |
| TCGA-EB-A44Q | 1.156164 | 0 | 8.922381 | 3.908603 | 7.095916 | 6.671517 | 7.426424 | 6.62994 | 4.589812 | 3.990072 | 7.471659 | 6.90424 | 2.902233 | 4.561211 | 4.179202 | 1.224609 | 6.204989 |
| TCGA-DA-A1I7 | 7.405479 | 0 | 7.251809 | 5.739733 | 4.267899 | 5.795564 | 3.874195 | 4.318265 | 3.299967 | 4.327564 | 6.523763 | 4.524509 | 1.553008 | 2.711507 | 2.281568 | 1.357408 | 12.38458 |
| TCGA-D3-A8GP | 12.70685 | 0 | 7.222808 | 1.201613 | 3.576143 | 3.645775 | 2.180613 | 2.585903 | 1.166161 | 3.099083 | 7.546676 | 5.173722 | 0.553196 | 4.710364 | 1.075691 | 0.376379 | 8.669115 |
| TCGA-EE-A2GJ | 6.219178 | 1 | 7.730674 | 4.97959 | 5.846746 | 8.445874 | 6.656557 | 5.850582 | 5.922841 | 4.08262 | 6.97863 | 6.644516 | 3.160378 | 3.787229 | 3.367954 | 1.3242 | 7.28662 |
| TCGA-EE-A2A2 | 4.969863 | 0 | 7.129786 | 2.375512 | 4.143377 | 4.640419 | 4.952819 | 5.458939 | 3.452004 | 3.662747 | 7.133649 | 6.663957 | 1.979203 | 3.443325 | 4.274326 | 0.468979 | 7.397015 |
| TCGA-GN-A9SD | 4.950685 | 1 | 6.528434 | 3.363978 | 4.982164 | 5.963834 | 4.656576 | 5.552687 | 4.501412 | 2.864399 | 6.53286 | 5.548662 | 2.753822 | 3.91579 | 6.359379 | 0.593654 | 9.780437 |
| TCGA-D3-A8GQ | 2.421918 | 1 | 6.340385 | 1.975057 | 3.48753 | 5.388906 | 3.374235 | 2.08985 | 2.466216 | 3.273009 | 5.661771 | 6.639493 | 0.459596 | 1.054254 | 1.119869 | 0.746267 | 8.433625 |
| TCGA-D3-A1Q7 | 11.10411 | 0 | 9.274058 | 4.933278 | 8.167888 | 7.466493 | 9.025559 | 9.398771 | 6.163384 | 4.715788 | 6.831594 | 6.936064 | 3.21672 | 4.831046 | 4.359423 | 1.693231 | 8.936502 |
| TCGA-EB-A44P | 2.030137 | 0 | 8.370865 | 1.587732 | 4.678232 | 4.782303 | 3.563679 | 3.875326 | 1.320687 | 3.442993 | 8.589809 | 6.62527 | 0.540759 | 5.004481 | 3.429222 | 0.59671 | 9.993233 |
| TCGA-YD-A89C | 0.575342 | 0 | 3.543331 | 1.509052 | 1.795567 | 4.88639 | 1.403333 | 1.738793 | 1.67509 | 3.064629 | 5.349734 | 4.505409 | 0.318883 | 0.772119 | 0.944342 | 0.257026 | 7.013016 |
| TCGA-W3-AA1Q | 5.756164 | 1 | 7.209836 | 2.298622 | 5.28474 | 4.885795 | 4.839901 | 5.409396 | 3.023442 | 2.957029 | 6.72588 | 6.510921 | 1.804671 | 4.392843 | 2.959214 | 1.025757 | 9.226197 |
| TCGA-WE-A8K5 | 5.09589 | 1 | 7.359111 | 2.92835 | 5.019161 | 5.001114 | 6.21546 | 6.075771 | 3.237651 | 3.233094 | 7.023737 | 4.983483 | 2.111237 | 4.110687 | 2.181775 | 0.548444 | 8.033017 |
| TCGA-GN-A265 | 8.076712 | 0 | 8.825986 | 4.889377 | 6.99612 | 7.927317 | 7.251809 | 7.625453 | 6.189925 | 3.198592 | 7.431397 | 7.037601 | 3.921225 | 3.434209 | 4.766499 | 1.312527 | 10.58227 |
| TCGA-ER-A3ES | 20.5863 | 1 | 3.141248 | 0.803807 | 1.609841 | 2.095893 | 0.608724 | 0.169733 | 0.880399 | 3.586131 | 5.51279 | 3.744508 | 0.119587 | 4.452162 | 0.373077 | 0.123859 | 5.843018 |
| TCGA-ER-A42H | 1.167123 | 1 | 7.982479 | 2.522271 | 1.811211 | 4.809578 | 1.887661 | 1.44057 | 2.319232 | 2.719167 | 7.177377 | 4.881582 | 0.869994 | 3.489855 | 0.43904 | 0.133792 | 8.196775 |
| TCGA-EE-A2ME | 8.605479 | 1 | 7.803093 | 4.743614 | 8.312242 | 6.661502 | 8.300745 | 9.025559 | 6.731229 | 3.723749 | 6.33485 | 6.161723 | 4.243505 | 4.414015 | 7.149764 | 1.091317 | 8.733064 |
| TCGA-FW-A3TU | 4.632877 | 1 | 6.736535 | 3.07053 | 4.595878 | 5.423107 | 1.30369 | 1.48969 | 2.329183 | 2.755356 | 7.809954 | 5.479689 | 1.067463 | 4.353189 | 1.190527 | 0.879439 | 8.852298 |
| TCGA-FS-A1ZS | 12.4 | 0 | 4.967484 | 4.19707 | 2.454422 | 5.816826 | 2.177416 | 1.637717 | 3.592395 | 4.532967 | 6.988917 | 2.522833 | 1.141914 | 1.262215 | 2.192578 | 1.475829 | 11.98144 |
| TCGA-EB-A5UM | 2.134247 | 0 | 6.771667 | 1.646938 | 6.078894 | 4.786798 | 4.277326 | 6.571463 | 2.705028 | 2.856448 | 8.773145 | 4.610576 | 1.968246 | 4.141938 | 4.31783 | 0.295059 | 8.549656 |
| TCGA-D3-A8GN | 13.41644 | 0 | 9.289949 | 2.876777 | 6.828652 | 6.089466 | 5.613388 | 5.785798 | 3.308653 | 2.562021 | 6.374689 | 6.98562 | 2.88393 | 5.031832 | 2.962654 | 0.902613 | 8.950546 |
| TCGA-EB-A97M | 1.134247 | 0 | 8.370865 | 3.369934 | 5.312731 | 5.742103 | 4.225862 | 4.818882 | 3.451675 | 2.620674 | 10.73066 | 5.798016 | 1.183874 | 3.32231 | 2.541575 | 0.94615 | 9.564239 |
| TCGA-D3-A2JA | 9.627397 | 0 | 9.088515 | 4.686696 | 5.50116 | 7.129786 | 6.718114 | 4.778292 | 5.465529 | 5.398703 | 7.247635 | 5.029761 | 3.087763 | 4.287014 | 3.376198 | 1.106037 | 11.24696 |
| TCGA-YG-AA3O | 3.161644 | 1 | 4.984792 | 2.918325 | 4.222926 | 4.736566 | 3.099405 | 4.36262 | 3.040455 | 3.0081 | 7.251809 | 5.205213 | 1.501183 | 3.146348 | 1.970555 | 0.758349 | 7.317853 |
| TCGA-D3-A8GC | 6.632877 | 1 | 6.744385 | 2.941746 | 3.22275 | 4.389632 | 2.475585 | 3.143169 | 3.249943 | 3.927388 | 6.62756 | 6.895262 | 1.443935 | 3.203457 | 1.944055 | 0.901619 | 9.258211 |
| TCGA-BF-A1PZ | 2.336986 | 0 | 5.890512 | 1.874332 | 4.282627 | 4.487516 | 3.79199 | 3.741678 | 1.74147 | 3.740233 | 8.617038 | 5.11905 | 0.755487 | 4.490361 | 2.341522 | 0.828764 | 9.241958 |
| TCGA-ER-A19T | 0.739726 | 1 | 7.020203 | 3.752834 | 4.906628 | 6.248828 | 3.849962 | 4.157889 | 4.193273 | 2.868418 | 7.177377 | 5.599669 | 2.413896 | 3.747772 | 2.37796 | 0.8692 | 8.471285 |
| TCGA-GF-A6C9 | 1.315068 | 0 | 9.166141 | 4.500432 | 6.851959 | 7.149764 | 5.485044 | 7.209836 | 5.406713 | 3.519033 | 6.92965 | 5.789443 | 3.997879 | 4.254682 | 5.097254 | 1.646331 | 9.669984 |
| TCGA-FW-A5DY | 1.608219 | 0 | 8.589809 | 3.224377 | 5.729288 | 5.949985 | 4.298003 | 5.148215 | 5.300255 | 3.205702 | 7.53005 | 6.910664 | 2.863812 | 3.900945 | 6.916855 | 0.941144 | 9.289949 |
| TCGA-FR-A8YC | 2.90137 | 1 | 8.746058 | 2.218721 | 4.992628 | 6.034935 | 3.979563 | 2.442892 | 3.234068 | 3.427134 | 5.642036 | 6.156901 | 1.480583 | 3.47433 | 1.207599 | 0.326074 | 8.523571 |
| TCGA-D9-A149 | 4.556164 | 0 | 10.06923 | 3.816993 | 6.62994 | 5.432151 | 6.382608 | 5.851858 | 4.553304 | 4.908506 | 7.557357 | 5.856967 | 1.892172 | 5.378328 | 3.942233 | 1.008048 | 7.524388 |
| TCGA-EE-A29G | 6.005479 | 1 | 7.103381 | 2.833343 | 5.07548 | 5.162736 | 3.525903 | 5.279117 | 3.994754 | 2.853365 | 6.733851 | 4.604945 | 4.801623 | 2.394356 | 2.00615 | 2.248151 | 8.669115 |
| TCGA-FS-A1YY | 19.04932 | 1 | 3.87646 | 2.613169 | 2.524963 | 4.710907 | 1.192809 | 1.321489 | 2.502921 | 2.64811 | 8.177475 | 4.552299 | 1.192225 | 2.709027 | 0.99518 | 0.800836 | 8.980385 |
| TCGA-EE-A3J4 | 10.6 | 1 | 9.166141 | 2.61737 | 4.383207 | 4.843982 | 6.400558 | 5.360016 | 3.169336 | 3.955781 | 6.819979 | 5.675939 | 1.363786 | 4.774271 | 1.84066 | 0.838404 | 8.196775 |
| TCGA-EE-A20B | 11.15068 | 0 | 7.291223 | 3.870067 | 5.601701 | 6.566829 | 4.555289 | 5.007785 | 3.869708 | 2.728091 | 7.027108 | 6.534983 | 2.028004 | 2.935261 | 2.134387 | 0.929164 | 8.980385 |
| TCGA-EB-A4IQ | 1.742466 | 1 | 3.833744 | 0.983609 | 3.115558 | 4.116779 | 0.224635 | 0.880791 | 1.012613 | 2.923659 | 8.128757 | 5.529475 | 3.662747 | 2.592274 | 0.684612 | 1.419846 | 7.002926 |
| TCGA-EE-A29M | 4.736986 | 0 | 9.901675 | 2.994039 | 5.458939 | 5.03317 | 5.218926 | 6.05292 | 3.648567 | 4.508773 | 6.666452 | 6.62527 | 1.286892 | 4.890622 | 4.368106 | 0.616893 | 7.461545 |
| TCGA-FS-A1ZM | 8.438356 | 0 | 6.599065 | 4.528953 | 6.718114 | 7.173327 | 5.772846 | 6.07728 | 4.99396 | 4.025791 | 8.509861 | 5.899719 | 2.508323 | 3.936106 | 2.548226 | 0.91097 | 10.27357 |
| TCGA-BF-A5ES | 1.342466 | 0 | 6.338468 | 3.11962 | 4.901586 | 5.506029 | 3.843007 | 3.830027 | 3.231814 | 2.672874 | 9.616823 | 6.438997 | 1.404704 | 4.21053 | 2.505939 | 1.291459 | 8.334715 |
| TCGA-EE-A2GK | 4.561644 | 0 | 7.205721 | 3.363006 | 7.304577 | 6.027505 | 4.26065 | 6.040997 | 6.384619 | 2.714566 | 6.277134 | 5.712048 | 3.934614 | 2.80007 | 8.359438 | 0.65707 | 7.991229 |
| TCGA-DA-A960 | 2.20274 | 0 | 4.60036 | 1.072241 | 2.082031 | 3.356564 | 0.41118 | 1.089713 | 0.992883 | 3.426122 | 9.724841 | 4.685062 | 0.788382 | 2.356478 | 0.543494 | 0.391353 | 7.373601 |
| TCGA-GN-A26A | 2.706849 | 1 | 6.118025 | 4.681845 | 5.349734 | 7.359111 | 4.450264 | 5.425822 | 6.086502 | 2.943907 | 6.394526 | 6.855134 | 2.916799 | 4.041394 | 3.161333 | 1.925868 | 12.12313 |
| TCGA-GN-A264 | 9.827397 | 1 | 4.307652 | 2.776986 | 3.021232 | 4.596887 | 2.056158 | 2.499893 | 2.915268 | 2.807377 | 6.61586 | 3.731561 | 1.27655 | 3.045833 | 1.124969 | 0.509436 | 6.965222 |
| TCGA-EB-A3XE | 0.493151 | 0 | 6.461754 | 2.059019 | 5.415779 | 4.985434 | 4.515673 | 5.745558 | 1.449764 | 2.323708 | 8.603012 | 5.142362 | 3.675273 | 4.765952 | 2.614656 | 0.749656 | 9.818344 |
| TCGA-FW-A5DX | 1.753425 | 0 | 6.704789 | 2.195256 | 2.848759 | 3.763495 | 2.747434 | 3.21513 | 2.666239 | 3.527986 | 3.40407 | 4.492787 | 0.563661 | 2.660468 | 2.275852 | 0.328167 | 9.818344 |
