# Supplementary material for: A novel inflammatory response-related signature predicts the prognosis of cutaneous melanoma and the effect of antitumor drugs
Source: World J Surg Oncol. 2022 Aug 19;20:263. doi: 10.1186/s12957-022-02726-8 (PMC9389732; doi:10.1186/s12957-022-02726-8)
Supplement: Supplementary file 3 — Additional file 3. [file 12957_2022_2726_MOESM3_ESM.docx]

| id | futime | fustat | BST2 | C3AR1 | CCL5 | CD14 | CXCL10 | CXCL9 | CYBB | EIF2AK2 | EMP3 | ICAM1 | IL18 | RTP4 | SELL | SLC31A2 | TIMP1 |
| --- | --- | --- | --- | --- | --- | --- | --- | --- | --- | --- | --- | --- | --- | --- | --- | --- | --- |
| TCGA-EB-A550 | 0.723288 | 1 | 8.603012 | 1.34888 | 4.206316 | 5.050004 | 3.229838 | 2.296967 | 1.71957 | 2.954205 | 7.982479 | 3.65926 | 3.144796 | 4.128244 | 1.336658 | 1.388472 | 8.720292 |
| TCGA-EB-A431 | 1.556164 | 0 | 7.631178 | 1.757384 | 3.128146 | 3.849596 | 3.350769 | 3.290567 | 1.703156 | 3.717236 | 6.140573 | 5.169968 | 0.384948 | 4.610032 | 1.846324 | 0.654263 | 7.0306 |
| TCGA-EE-A29H | 5.386301 | 0 | 7.749891 | 2.731468 | 4.138497 | 4.813655 | 3.773381 | 4.302418 | 3.42819 | 3.564015 | 6.843095 | 5.202875 | 2.022954 | 3.747772 | 3.211878 | 0.708107 | 9.564239 |
| TCGA-D3-A2JD | 0.989041 | 1 | 7.16174 | 2.709027 | 4.460544 | 4.808396 | 1.3242 | 2.455629 | 3.332851 | 2.0577 | 7.61381 | 5.353938 | 1.524044 | 3.490878 | 5.988206 | 0.899785 | 9.947093 |
| TCGA-BF-AAP1 | 1.120548 | 0 | 9.041563 | 1.84066 | 2.415992 | 4.668222 | 1.858245 | 1.62848 | 2.418992 | 2.517122 | 6.239887 | 5.45063 | 0.81239 | 4.485131 | 0.96028 | 0.442093 | 7.111111 |
| TCGA-GN-A268 | 5.232877 | 1 | 4.997862 | 2.41542 | 2.562021 | 4.344293 | 2.435875 | 1.71016 | 2.379128 | 4.230092 | 7.601866 | 5.204438 | 0.592552 | 2.876461 | 1.426658 | 0.653309 | 7.660811 |
| TCGA-GF-A769 | 2.931507 | 1 | 7.340424 | 2.731166 | 2.145806 | 5.699542 | 1.985801 | 1.345127 | 2.606778 | 3.147012 | 7.519161 | 6.382608 | 0.781019 | 2.071486 | 0.632614 | 1.085059 | 10.89923 |
| TCGA-FS-A1ZQ | 11.12877 | 1 | 4.98281 | 2.560681 | 3.706065 | 5.605891 | 1.742047 | 2.405914 | 2.881736 | 2.880497 | 6.620447 | 5.787002 | 0.774442 | 3.445976 | 3.216395 | 0.590901 | 9.463852 |
| TCGA-EE-A2A1 | 9.663014 | 0 | 8.024331 | 4.553304 | 6.502157 | 6.907455 | 5.686046 | 7.209836 | 5.358285 | 3.655719 | 6.861068 | 5.96242 | 2.760947 | 4.060013 | 5.035834 | 1.121271 | 7.596001 |
| TCGA-EE-A3JD | 2.279452 | 1 | 6.410422 | 4.986055 | 6.99958 | 6.347924 | 7.373601 | 7.557357 | 6.103696 | 3.892887 | 6.715439 | 6.641932 | 3.808337 | 4.129481 | 6.741728 | 1.201613 | 9.859751 |
| TCGA-EE-A3AH | 11.56712 | 1 | 6.278997 | 2.251769 | 1.856485 | 5.329508 | 1.799696 | 2.394038 | 2.227158 | 3.328267 | 5.850582 | 7.317853 | 0.369979 | 3.059914 | 1.456486 | 0.413017 | 8.359438 |
| TCGA-XV-AAZV | 1.128767 | 0 | 8.656072 | 4.956622 | 5.33779 | 7.456423 | 5.380093 | 4.770916 | 4.463359 | 2.79366 | 8.523571 | 4.373953 | 3.94299 | 3.774114 | 2.894781 | 1.598286 | 12.50929 |
| TCGA-D3-A51N | 1.884932 | 0 | 10.09768 | 3.726952 | 5.476823 | 7.81695 | 6.289969 | 6.23809 | 4.469498 | 3.818891 | 7.304577 | 6.747052 | 2.555408 | 5.172976 | 3.438617 | 0.915708 | 10.89923 |
| TCGA-EE-A29N | 1.550685 | 1 | 6.138973 | 3.471208 | 5.831696 | 6.151984 | 5.619622 | 5.664068 | 5.027048 | 2.810137 | 8.617038 | 7.336058 | 2.354949 | 4.007695 | 4.88639 | 1.282663 | 9.924688 |
| TCGA-EB-A3Y7 | 0.893151 | 1 | 4.839901 | 2.593764 | 3.939999 | 4.496554 | 1.525813 | 0.716023 | 0.724927 | 2.537122 | 10.01891 | 5.505052 | 0.214853 | 3.67597 | 1.252467 | 0.990474 | 9.838554 |
| TCGA-EE-A184 | 5.679452 | 1 | 4.533466 | 2.953905 | 5.813069 | 4.961771 | 3.773753 | 5.780976 | 3.719762 | 2.918624 | 7.660811 | 6.261111 | 1.820111 | 4.298003 | 2.753822 | 0.851609 | 7.16174 |
| TCGA-EB-A5KH | 1.69589 | 1 | 3.898671 | 2.052277 | 3.32231 | 4.14787 | 1.325728 | 2.03512 | 1.619826 | 2.834896 | 7.717973 | 4.458699 | 0.788166 | 4.628097 | 2.666239 | 0.459291 | 8.226828 |
| TCGA-D3-A2J7 | 8.591781 | 1 | 8.81294 | 4.291411 | 6.758168 | 5.292188 | 5.056886 | 6.208536 | 3.637653 | 4.490361 | 7.911462 | 6.445178 | 2.11336 | 4.641992 | 3.088057 | 1.223048 | 10.01891 |
| TCGA-EE-A2GE | 14.48219 | 0 | 6.796978 | 4.970068 | 7.769483 | 7.508591 | 7.239183 | 7.451022 | 6.005248 | 3.161989 | 6.273499 | 7.919211 | 3.154594 | 4.01763 | 4.564648 | 1.324699 | 10.27357 |
| TCGA-EE-A3J8 | 2.860274 | 1 | 6.694161 | 2.48312 | 1.621206 | 2.870886 | 1.370693 | 0.727074 | 2.251473 | 3.900197 | 6.095718 | 5.306915 | 0.845631 | 2.758389 | 0.138582 | 0.081743 | 7.831019 |
| TCGA-D3-A2JC | 7.230137 | 0 | 9.180879 | 3.530726 | 6.332961 | 6.817203 | 5.081679 | 6.161723 | 4.585658 | 2.84142 | 7.911462 | 8.576339 | 2.259543 | 4.050393 | 2.68983 | 1.043435 | 10.45413 |
| TCGA-EE-A2MF | 22.39452 | 1 | 10.34278 | 3.788696 | 4.524991 | 7.431397 | 4.986055 | 3.728385 | 4.721791 | 4.823581 | 6.369056 | 5.377476 | 1.822614 | 6.254056 | 2.657507 | 0.816154 | 8.312242 |
| TCGA-D3-A3C8 | 3.860274 | 0 | 9.274058 | 4.7306 | 10.04373 | 6.962009 | 9.780437 | 9.859751 | 5.757281 | 3.610867 | 7.201831 | 6.939334 | 4.577092 | 5.127742 | 5.537538 | 1.633665 | 9.598808 |
| TCGA-W3-AA1O | 0.334247 | 1 | 7.009619 | 2.511042 | 2.442243 | 4.391455 | 1.998625 | 1.754429 | 2.046864 | 3.435915 | 8.799477 | 5.885366 | 0.803119 | 2.719471 | 1.258626 | 1.379524 | 8.471285 |
| TCGA-FS-A1ZW | 4.123288 | 0 | 6.85809 | 4.233522 | 5.76205 | 4.995278 | 7.39195 | 7.177377 | 6.072573 | 3.56056 | 6.078894 | 5.733907 | 3.068983 | 3.750307 | 3.037302 | 0.646868 | 8.101991 |
| TCGA-FS-A1ZY | 2.257534 | 1 | 6.712726 | 1.792038 | 2.172807 | 4.322751 | 1.518595 | 0.912356 | 1.461685 | 2.957029 | 7.643215 | 6.252268 | 0.368905 | 3.460752 | 0.961107 | 0.319493 | 7.692498 |
| TCGA-D3-A2J8 | 5.457534 | 1 | 8.681574 | 3.677019 | 7.942817 | 6.649415 | 7.846293 | 8.346721 | 5.185717 | 3.986946 | 7.573321 | 5.944383 | 2.072692 | 3.895594 | 4.862844 | 1.745809 | 9.398771 |
| TCGA-EB-A1NK | 2.846575 | 0 | 4.425512 | 2.646009 | 3.857984 | 5.773992 | 2.535863 | 2.879227 | 2.29393 | 2.732996 | 7.273262 | 5.659605 | 2.184762 | 3.101634 | 1.585189 | 1.170998 | 8.89291 |
| TCGA-FS-A1ZP | 6.227397 | 1 | 5.670395 | 3.942233 | 4.360793 | 5.002503 | 3.674908 | 3.752106 | 4.678232 | 3.230497 | 5.690545 | 5.045242 | 2.126313 | 4.798207 | 4.401267 | 0.511744 | 7.440839 |
| TCGA-ER-A19K | 1.284932 | 1 | 6.027505 | 2.543405 | 5.021107 | 4.790193 | 4.845175 | 4.88639 | 2.861328 | 3.742744 | 8.669115 | 5.934947 | 2.523775 | 3.780599 | 1.924649 | 1.310142 | 7.235035 |
| TCGA-EE-A2A6 | 7.178082 | 0 | 7.551771 | 3.30512 | 6.785484 | 5.936317 | 5.333692 | 5.978058 | 3.56056 | 3.312502 | 8.033017 | 7.643215 | 2.03423 | 4.469498 | 4.402595 | 0.795586 | 9.196354 |
| TCGA-WE-A8ZT | 0.983562 | 0 | 6.166742 | 1.751768 | 2.316872 | 3.824402 | 2.346885 | 3.085264 | 2.009791 | 3.09347 | 6.105212 | 4.807313 | 0.815671 | 4.148305 | 3.315406 | 0.350629 | 8.70773 |
| TCGA-EE-A29D | 1.164384 | 1 | 6.610932 | 2.711829 | 2.584987 | 5.265428 | 1.494288 | 1.604235 | 2.31472 | 4.204152 | 8.537098 | 5.552687 | 1.364818 | 3.398386 | 1.53493 | 0.577175 | 8.370865 |
| TCGA-EE-A185 | 0.413699 | 1 | 6.659034 | 1.543139 | 1.144952 | 3.038838 | 0.725989 | 0.299319 | 0.572845 | 3.197918 | 9.180879 | 6.639493 | 0.843999 | 2.568631 | 0.617715 | 1.069808 | 7.185193 |
| TCGA-EB-A4IS | 2.120548 | 0 | 7.730674 | 3.04264 | 6.430613 | 6.204989 | 6.916855 | 7.129786 | 4.681845 | 4.27996 | 8.158476 | 5.983812 | 2.965733 | 4.945241 | 4.587197 | 0.578772 | 9.651786 |
| TCGA-ER-A19J | 0.536986 | 1 | 7.705489 | 3.004961 | 3.518044 | 6.222321 | 2.889819 | 2.115523 | 3.884151 | 3.367285 | 5.082352 | 6.030441 | 1.506581 | 3.768598 | 1.615407 | 0.633312 | 9.241958 |
| TCGA-FR-A3R1 | 1.876712 | 0 | 9.879919 | 2.941451 | 5.522496 | 4.744711 | 6.466031 | 6.394526 | 3.199867 | 4.458699 | 7.269034 | 6.316095 | 1.427864 | 5.81798 | 1.737325 | 1.07791 | 8.617038 |
| TCGA-YG-AA3N | 0.838356 | 0 | 8.289803 | 3.465535 | 8.549656 | 7.013016 | 4.102131 | 5.51091 | 3.768234 | 2.963276 | 8.167888 | 5.850582 | 3.101929 | 3.813297 | 2.839236 | 1.527073 | 8.408325 |
| TCGA-FS-A1ZU | 2.213699 | 1 | 4.581565 | 3.091858 | 1.526247 | 6.201606 | 2.024711 | 1.190262 | 3.559866 | 2.882048 | 6.236283 | 5.047933 | 1.09355 | 0.986259 | 1.164764 | 0.601914 | 6.87963 |
| TCGA-D3-A2JN | 5.539726 | 1 | 7.911462 | 2.833654 | 6.901181 | 5.976614 | 5.127009 | 5.788227 | 3.175691 | 2.997456 | 6.573981 | 5.232895 | 1.755014 | 2.463821 | 3.697354 | 0.422899 | 11.329 |
| TCGA-EB-A5SF | 1.010959 | 1 | 4.147055 | 2.028276 | 3.131884 | 4.872503 | 0.400789 | 0.583248 | 0.969737 | 1.23555 | 6.97555 | 4.373506 | 3.693721 | 3.404412 | 0.743422 | 2.585589 | 6.656557 |
| TCGA-EB-A42Y | 1.975342 | 1 | 9.13588 | 2.900991 | 3.98613 | 5.726922 | 3.088057 | 1.975622 | 2.748343 | 4.100065 | 7.584319 | 5.370323 | 0.950176 | 4.223791 | 0.941768 | 0.736982 | 6.21546 |
| TCGA-FS-A1Z7 | 0.649315 | 1 | 6.555404 | 3.694812 | 3.209917 | 6.671517 | 1.814168 | 2.669531 | 4.010395 | 3.008703 | 6.211944 | 6.289969 | 2.068779 | 2.721883 | 2.480396 | 1.03782 | 9.598808 |
| TCGA-D3-A8GI | 4.876712 | 1 | 7.145736 | 2.565945 | 3.162624 | 5.427656 | 2.33005 | 2.017854 | 1.495854 | 2.678119 | 9.547666 | 5.947206 | 1.024124 | 2.820963 | 1.347495 | 1.033988 | 10.06923 |
| TCGA-D9-A3Z3 | 1.857534 | 0 | 6.181775 | 3.422461 | 5.63984 | 6.21372 | 4.346959 | 5.543646 | 3.830411 | 2.211545 | 7.081368 | 5.655324 | 2.7921 | 4.170381 | 4.4418 | 1.504818 | 9.103504 |
| TCGA-D9-A6EC | 6.463014 | 0 | 10.34278 | 2.449949 | 4.154915 | 4.935834 | 4.678232 | 3.028717 | 2.890431 | 3.746323 | 7.436078 | 7.769483 | 1.188799 | 6.075771 | 0.725111 | 0.581846 | 9.547666 |
| TCGA-EE-A17X | 2.484932 | 1 | 8.049629 | 2.632861 | 3.009026 | 4.856951 | 1.550417 | 1.814168 | 2.247835 | 3.005266 | 7.482043 | 5.047933 | 0.854026 | 1.633116 | 1.577199 | 1.193733 | 8.537098 |
| TCGA-DA-A1I4 | 2.994521 | 1 | 7.482043 | 4.506852 | 4.255091 | 7.114687 | 2.931155 | 2.390172 | 5.159824 | 3.276915 | 8.773145 | 6.596799 | 2.71977 | 3.8975 | 3.747061 | 1.173692 | 10.41592 |
| TCGA-EE-A2A0 | 3.90137 | 1 | 5.94583 | 5.047231 | 6.018783 | 5.115437 | 6.140573 | 6.843095 | 6.071016 | 3.547145 | 5.983812 | 5.319401 | 2.873389 | 4.150773 | 5.173722 | 0.750313 | 7.974782 |
| TCGA-ER-A2ND | 1.945205 | 1 | 4.995937 | 2.64811 | 4.944608 | 5.480695 | 3.586486 | 4.63581 | 2.211545 | 3.335846 | 8.007828 | 3.86005 | 2.002568 | 4.110687 | 2.007047 | 0.20586 | 10.15165 |
| TCGA-EB-A551 | 1.616438 | 0 | 9.616823 | 4.291856 | 7.378207 | 6.98562 | 6.988917 | 8.167888 | 5.299455 | 4.328415 | 6.599065 | 6.817203 | 4.414923 | 5.255055 | 3.381555 | 1.096736 | 9.289949 |
| TCGA-YG-AA3P | 1.20274 | 0 | 4.358096 | 2.058281 | 2.594386 | 4.900339 | 4.604945 | 3.589658 | 3.379914 | 3.164196 | 5.502088 | 2.162273 | 1.041155 | 3.082125 | 1.187375 | 0.477007 | 5.316857 |
| TCGA-ER-A19F | 2.19726 | 1 | 5.632326 | 2.968828 | 4.023035 | 4.823581 | 4.590822 | 4.331996 | 3.4031 | 3.454979 | 8.226828 | 5.744404 | 2.046549 | 3.493236 | 2.437383 | 0.563407 | 8.279393 |
| TCGA-EB-A41B | 0.79726 | 0 | 3.727687 | 1.826336 | 4.866415 | 4.319616 | 4.199599 | 3.871904 | 1.723899 | 3.575111 | 7.055493 | 5.809374 | 0.805552 | 3.110448 | 0.76196 | 1.087596 | 7.982479 |
| TCGA-BF-A5ER | 0.89589 | 0 | 8.247378 | 2.230789 | 3.631373 | 4.231828 | 1.029545 | 1.120579 | 1.571825 | 2.350708 | 9.241958 | 6.861068 | 0.707767 | 3.20378 | 1.362115 | 0.823491 | 9.530744 |
| TCGA-D3-A51J | 12.09315 | 0 | 9.947093 | 4.007291 | 7.48764 | 7.749891 | 6.384619 | 7.958578 | 4.971331 | 3.633494 | 6.481044 | 7.059001 | 3.533102 | 3.965917 | 4.029376 | 0.84337 | 10.18136 |
| TCGA-D3-A8GV | 13.97534 | 1 | 5.095098 | 2.312063 | 2.910762 | 4.727299 | 1.668705 | 2.325819 | 1.542595 | 3.148267 | 8.359438 | 5.297867 | 0.49743 | 3.183843 | 2.028903 | 0.681187 | 9.289949 |
| TCGA-GN-A262 | 11.65753 | 0 | 6.097304 | 0.05263 | 0.556626 | 1.000594 | 5.085244 | 3.930439 | 2.287618 | 3.126907 | 6.445178 | 4.315645 | 0.000883 | 3.432196 | 0.384417 | 0.038203 | 8.936502 |
| TCGA-FR-A7UA | 3.189041 | 0 | 7.440839 | 5.788227 | 9.398771 | 8.48398 | 9.289949 | 9.119603 | 6.768916 | 3.260307 | 7.300321 | 7.631178 | 4.88759 | 5.110426 | 4.970068 | 2.087744 | 9.879919 |
| TCGA-EE-A180 | 7.915068 | 1 | 9.924688 | 2.497156 | 3.632453 | 4.462887 | 4.637854 | 3.713631 | 2.519863 | 4.682917 | 9.119603 | 5.616495 | 0.695351 | 4.899716 | 1.697879 | 0.490165 | 8.496625 |
| TCGA-RP-A690 | 0.016438 | 0 | 4.84106 | 2.970702 | 2.415707 | 4.783429 | 1.04671 | 1.704596 | 2.522833 | 3.267458 | 5.038563 | 4.639414 | 2.105454 | 4.301946 | 1.226053 | 0.470461 | 7.21417 |
| TCGA-DA-A95V | 6.008219 | 0 | 9.289949 | 2.909934 | 5.791894 | 6.168364 | 3.654295 | 4.615185 | 3.228875 | 2.473798 | 6.661502 | 5.963834 | 1.816788 | 4.327103 | 3.277559 | 0.714973 | 7.831019 |
| TCGA-FR-A8YE | 8.70137 | 0 | 6.220561 | 4.39009 | 4.813115 | 7.535743 | 3.739515 | 5.084513 | 5.766811 | 2.965399 | 6.418429 | 6.089466 | 3.168052 | 3.625383 | 6.822967 | 1.427736 | 9.724841 |
| TCGA-W3-AA1V | 3.506849 | 1 | 5.279117 | 3.850703 | 5.991046 | 6.369056 | 4.696811 | 4.395625 | 4.792996 | 3.67597 | 7.129786 | 3.584073 | 2.12119 | 1.939614 | 1.177324 | 1.995372 | 9.879919 |
| TCGA-3N-A9WD | 1.082192 | 1 | 7.776094 | 2.214857 | 5.50116 | 6.763519 | 6.483103 | 6.037932 | 3.070835 | 3.157822 | 7.256045 | 5.793139 | 2.397101 | 3.832286 | 4.626564 | 3.531747 | 8.936502 |
| TCGA-ER-A19D | 1.049315 | 1 | 5.568744 | 4.460973 | 5.577851 | 7.118484 | 5.107671 | 6.472349 | 5.605891 | 3.567468 | 5.207525 | 4.998482 | 2.434353 | 1.525951 | 3.00964 | 0.794365 | 9.581285 |
| TCGA-FS-A1ZZ | 2.252055 | 1 | 9.025559 | 1.85136 | 3.911984 | 2.671071 | 3.545088 | 3.190246 | 1.631073 | 5.003138 | 8.879077 | 4.040586 | 0.912617 | 5.493292 | 1.264239 | 0.787701 | 7.590229 |
| TCGA-ER-A2NF | 2.40274 | 1 | 7.756158 | 3.505499 | 5.869628 | 5.971011 | 4.87554 | 5.331182 | 3.945691 | 4.03452 | 5.297867 | 4.728934 | 2.036355 | 4.516663 | 3.421152 | 0.642079 | 7.904414 |
| TCGA-EE-A2GO | 10.56712 | 0 | 6.840305 | 2.957646 | 1.972089 | 5.296264 | 1.300221 | 1.357672 | 1.806449 | 3.291225 | 4.164179 | 4.032905 | 0.43748 | 1.206357 | 0.869404 | 3.298362 | 11.98144 |
| TCGA-D3-A5GT | 1.334247 | 0 | 7.411642 | 1.270826 | 4.219851 | 3.070228 | 0.998782 | 0.664252 | 1.087361 | 2.22143 | 8.346721 | 4.066859 | 0.948632 | 4.5538 | 0.461365 | 0.111626 | 8.158476 |
| TCGA-BF-A1Q0 | 2.276712 | 0 | 7.666866 | 2.713648 | 4.573066 | 4.519082 | 4.713641 | 4.522992 | 3.493926 | 3.194381 | 7.471659 | 5.356568 | 1.825443 | 4.059586 | 2.700163 | 1.415816 | 10.45413 |
| TCGA-EB-A3XC | 1.780822 | 0 | 7.513933 | 2.842345 | 3.905211 | 5.186453 | 4.346531 | 4.405361 | 2.697148 | 4.100065 | 7.205721 | 4.88095 | 2.502632 | 3.213533 | 1.686155 | 1.418553 | 7.982479 |
| TCGA-FS-A1ZF | 1.287671 | 1 | 7.831019 | 1.842163 | 3.904848 | 4.592808 | 3.053979 | 2.962654 | 2.107597 | 2.590479 | 6.374689 | 4.215249 | 0.710433 | 4.159938 | 1.598558 | 0.293358 | 9.119603 |
| TCGA-D3-A2JK | 1.008219 | 1 | 5.664068 | 2.591083 | 4.661816 | 6.239887 | 1.796143 | 1.320326 | 2.211099 | 2.098889 | 9.088515 | 5.063013 | 2.056642 | 3.94299 | 1.581805 | 0.333694 | 11.16651 |
| TCGA-3N-A9WC | 5.539726 | 0 | 9.947093 | 3.871172 | 7.711737 | 6.398654 | 6.744385 | 8.720292 | 4.497974 | 4.700048 | 5.566737 | 6.739135 | 3.430883 | 5.729288 | 4.028589 | 1.228231 | 9.742234 |
| TCGA-GN-A4U4 | 3.279452 | 0 | 8.119652 | 1.55527 | 2.849967 | 3.04898 | 3.09347 | 3.023733 | 1.795295 | 3.265156 | 6.75249 | 5.884054 | 0.959882 | 3.129058 | 2.691047 | 0.464097 | 6.481044 |
| TCGA-ER-A19C | 4.073973 | 1 | 5.366855 | 2.953905 | 3.144482 | 4.680326 | 1.998918 | 2.41747 | 2.618891 | 2.853669 | 7.743395 | 5.105564 | 0.894782 | 3.858157 | 1.190006 | 0.739099 | 9.838554 |
| TCGA-ER-A197 | 1.161644 | 1 | 7.535743 | 4.058005 | 7.456423 | 8.206697 | 5.51091 | 4.753596 | 5.583976 | 3.531406 | 7.698557 | 6.280835 | 2.813533 | 3.414729 | 2.866566 | 1.191375 | 9.780437 |
| TCGA-EE-A20H | 14.02192 | 1 | 5.103458 | 1.550766 | 2.475585 | 3.433869 | 2.829887 | 4.408494 | 2.069698 | 4.012809 | 6.206777 | 1.857346 | 0.919295 | 2.40683 | 1.256297 | 0.439893 | 10.09768 |
| TCGA-EE-A2MP | 20.72055 | 0 | 9.305759 | 3.902104 | 6.515039 | 7.28662 | 5.897063 | 5.979426 | 4.686696 | 2.914343 | 6.113318 | 5.954178 | 2.284002 | 4.86398 | 2.682161 | 1.281175 | 9.970523 |
| TCGA-EB-A82C | 0.046575 | 0 | 4.472309 | 1.170261 | 3.127204 | 3.121851 | 2.62663 | 1.625312 | 1.092527 | 3.147961 | 8.950546 | 5.271799 | 0.54685 | 4.682368 | 0.820736 | 0.550427 | 6.89809 |
| TCGA-WE-AAA3 | 1.783562 | 0 | 9.398771 | 4.050393 | 6.255887 | 6.834409 | 4.65075 | 5.443306 | 4.702192 | 3.108527 | 7.373601 | 5.827863 | 2.09678 | 4.773707 | 2.47442 | 1.360626 | 7.874921 |
| TCGA-EB-A24C | 1.731507 | 0 | 5.129909 | 1.972641 | 2.240915 | 3.870067 | 2.970702 | 1.077009 | 2.078408 | 3.581976 | 8.119652 | 4.315645 | 1.808806 | 3.752834 | 0.928198 | 0.885503 | 8.206697 |
| TCGA-EE-A3AF | 1.150685 | 1 | 8.643297 | 4.033703 | 5.39604 | 5.688351 | 4.499939 | 5.344514 | 4.60036 | 2.958307 | 8.99563 | 6.447309 | 2.220801 | 3.633494 | 2.391384 | 1.290169 | 9.274058 |
| TCGA-EE-A2MD | 3.939726 | 1 | 7.189117 | 2.853046 | 4.600852 | 5.303511 | 4.056432 | 5.153381 | 3.621825 | 3.71512 | 4.821765 | 6.546306 | 1.699363 | 3.122163 | 3.253125 | 1.214037 | 7.331705 |
| TCGA-EB-A6QZ | 0.964384 | 1 | 7.426424 | 1.576617 | 2.7921 | 4.574577 | 1.9084 | 0.994994 | 1.705162 | 2.828042 | 8.049629 | 5.76205 | 0.462601 | 3.818498 | 1.03782 | 0.274721 | 9.634608 |
| TCGA-ER-A19N | 3.673973 | 1 | 7.397015 | 5.600651 | 6.840305 | 7.044609 | 5.146759 | 6.512953 | 5.516616 | 2.993432 | 7.889773 | 5.744404 | 3.327938 | 3.551313 | 4.055631 | 1.754104 | 9.13588 |
| TCGA-ER-A19Q | 4.241096 | 1 | 8.421176 | 4.955954 | 5.751385 | 7.373601 | 5.567773 | 7.062695 | 6.336661 | 3.270739 | 7.660811 | 6.808567 | 3.128768 | 3.028717 | 3.011496 | 1.496952 | 8.773145 |
| TCGA-DA-A1HY | 12.07397 | 0 | 9.320966 | 3.482835 | 2.213963 | 4.707098 | 2.936452 | 1.586611 | 2.740024 | 3.959265 | 5.263041 | 6.965222 | 0.730879 | 2.391907 | 2.219908 | 3.61897 | 12.50929 |
| TCGA-DA-A1HW | 3.00274 | 1 | 8.408325 | 3.928521 | 6.447309 | 6.674014 | 7.874921 | 8.226828 | 5.242299 | 3.238948 | 6.134201 | 6.455568 | 2.861005 | 4.252077 | 4.431118 | 1.280615 | 8.279393 |
| TCGA-GF-A3OT | 0.824658 | 0 | 5.177533 | 3.672426 | 5.571769 | 6.295651 | 5.686046 | 6.771667 | 4.241791 | 3.138316 | 8.033017 | 5.7549 | 2.064322 | 1.817393 | 1.696116 | 2.075054 | 9.367006 |
| TCGA-ER-A19H | 12.69589 | 1 | 5.383614 | 2.961692 | 5.518588 | 5.76205 | 7.551771 | 6.647021 | 4.490854 | 4.193273 | 6.380644 | 6.410422 | 1.614296 | 2.391384 | 3.065554 | 0.627451 | 7.111111 |
| TCGA-ER-A1A1 | 8.756164 | 0 | 6.825853 | 3.609114 | 5.543646 | 6.280835 | 4.105821 | 5.270234 | 5.932236 | 3.255716 | 5.722254 | 5.033871 | 4.28656 | 2.849655 | 8.033017 | 0.923131 | 8.549656 |
| TCGA-FS-A1ZR | 0.950685 | 1 | 6.939334 | 2.382689 | 5.399602 | 4.713121 | 6.257653 | 6.034935 | 3.212863 | 3.701397 | 7.023737 | 5.015125 | 1.319391 | 4.202079 | 3.046468 | 0.454763 | 9.258211 |
| TCGA-WE-A8ZO | 5.876712 | 0 | 7.81695 | 4.012809 | 6.622832 | 7.273262 | 6.663957 | 6.119597 | 5.295442 | 3.755025 | 6.831594 | 6.97555 | 3.018086 | 5.235234 | 2.145806 | 1.18953 | 8.950546 |
| TCGA-EE-A2GI | 4.060274 | 0 | 9.320966 | 3.256402 | 6.528434 | 5.909228 | 7.218327 | 6.277134 | 3.967864 | 3.707511 | 6.382608 | 6.347924 | 2.595555 | 5.731575 | 3.714017 | 0.603086 | 9.119603 |
| TCGA-GN-A4U5 | 3.167123 | 0 | 8.980385 | 2.80278 | 8.033017 | 5.538602 | 7.660811 | 6.945637 | 3.429222 | 3.974507 | 8.346721 | 6.189925 | 3.165808 | 4.758027 | 4.483235 | 1.073177 | 9.103504 |
| TCGA-EE-A17Z | 0.720548 | 1 | 5.943017 | 1.107242 | 2.074204 | 4.703304 | 0.90832 | 0.95928 | 1.252467 | 2.760947 | 6.227571 | 5.081004 | 0.890505 | 2.280994 | 0.693557 | 0.225781 | 7.177377 |
| TCGA-EE-A2M7 | 2.40274 | 1 | 9.463852 | 2.594988 | 4.281264 | 5.41944 | 2.143075 | 2.357063 | 3.623256 | 4.120044 | 5.81798 | 4.641466 | 1.348321 | 2.290586 | 5.906524 | 0.312244 | 9.367006 |
| TCGA-FR-A729 | 18.4 | 0 | 9.859751 | 3.778791 | 7.081368 | 6.758168 | 7.584319 | 7.705489 | 5.091615 | 3.239925 | 6.250568 | 6.666452 | 2.713962 | 4.550368 | 4.380385 | 0.753106 | 9.547666 |
| TCGA-EE-A2MC | 5.126027 | 1 | 7.78969 | 4.416715 | 7.535743 | 7.608113 | 6.98562 | 7.927317 | 6.048457 | 3.497665 | 6.72588 | 6.744385 | 2.655382 | 3.044881 | 3.752106 | 1.642616 | 10.15165 |
| TCGA-D3-A2JF | 5.172603 | 0 | 9.724841 | 3.482835 | 6.434817 | 6.523763 | 7.114687 | 6.510921 | 3.775181 | 3.708233 | 7.129786 | 6.009687 | 2.737577 | 5.538602 | 4.420402 | 0.991295 | 11.16651 |
| TCGA-BF-AAP4 | 0.917808 | 0 | 6.472349 | 2.881103 | 6.432736 | 5.523477 | 4.609009 | 5.524526 | 3.311222 | 2.896345 | 8.922381 | 6.046929 | 1.443381 | 4.7306 | 2.569803 | 1.014899 | 7.685976 |
| TCGA-ER-A19P | 13.50685 | 1 | 8.825986 | 5.147506 | 8.395557 | 7.145736 | 7.756158 | 8.669115 | 6.668834 | 3.251839 | 6.359379 | 6.29189 | 4.673509 | 4.428298 | 6.277134 | 1.248679 | 9.13588 |
| TCGA-EB-A4OY | 2.676712 | 0 | 5.107671 | 1.657501 | 5.360909 | 5.698423 | 2.104744 | 2.662876 | 1.475021 | 2.485859 | 9.415135 | 5.147506 | 1.034498 | 3.546793 | 1.3847 | 1.034852 | 8.799477 |
| TCGA-BF-A3DL | 2.106849 | 0 | 5.139475 | 2.231671 | 3.788341 | 4.832812 | 1.57121 | 2.569509 | 1.699363 | 2.415992 | 8.936502 | 5.546695 | 0.832887 | 3.920829 | 1.00229 | 0.884139 | 9.901675 |
| TCGA-EE-A181 | 2.810959 | 1 | 8.346721 | 3.780236 | 6.487264 | 6.342324 | 7.368556 | 7.551771 | 5.085952 | 3.679828 | 7.041155 | 6.777163 | 2.492855 | 4.460073 | 4.032905 | 1.093899 | 8.289803 |
| TCGA-ER-A19W | 12.34795 | 1 | 9.336361 | 5.438682 | 8.576339 | 7.966601 | 9.547666 | 9.463852 | 6.353506 | 3.739515 | 7.942817 | 6.694161 | 4.194532 | 4.999821 | 5.576846 | 1.732343 | 8.359438 |
| TCGA-EB-A85I | 0.991781 | 0 | 9.859751 | 3.079568 | 6.89809 | 5.263041 | 6.453496 | 5.100034 | 2.562304 | 3.438617 | 7.660811 | 6.53286 | 0.937352 | 4.982164 | 1.840336 | 1.868506 | 10.45413 |
| TCGA-EE-A2GM | 6.290411 | 0 | 6.336661 | 2.566247 | 3.810381 | 5.237616 | 3.470906 | 3.970644 | 2.942359 | 3.518044 | 7.239183 | 5.035168 | 1.116997 | 3.296091 | 1.238368 | 0.596601 | 9.196354 |
| TCGA-EE-A2GN | 8.509589 | 1 | 8.206697 | 2.908097 | 3.626814 | 5.50116 | 3.995519 | 3.782062 | 3.083978 | 3.487843 | 5.988206 | 5.587088 | 1.118399 | 3.021232 | 1.575824 | 0.637809 | 8.936502 |
| TCGA-BF-AAP7 | 0.871233 | 0 | 9.274058 | 3.782426 | 7.673388 | 6.828652 | 5.969603 | 6.116462 | 3.890592 | 3.469548 | 9.970523 | 6.07728 | 3.241524 | 4.055631 | 4.319184 | 0.860652 | 9.879919 |
| TCGA-D3-A3C6 | 4.838356 | 1 | 9.352444 | 2.447202 | 4.194962 | 5.198414 | 8.092881 | 2.187428 | 2.317176 | 3.549904 | 8.312242 | 6.257653 | 0.833946 | 5.192472 | 1.646938 | 0.685441 | 9.072918 |
| TCGA-DA-A3F3 | 0.873973 | 1 | 7.235035 | 2.594988 | 3.788696 | 5.102106 | 5.013166 | 4.649197 | 3.720832 | 3.378214 | 6.072573 | 6.037932 | 1.861425 | 4.752487 | 1.592835 | 0.56124 | 7.247635 |
| TCGA-ER-A2NE | 1.679452 | 1 | 7.133649 | 1.802638 | 1.970856 | 2.656253 | 0.633379 | 1.04866 | 0.8692 | 3.473979 | 7.273262 | 6.785484 | 0.301571 | 3.494908 | 1.333667 | 0.721516 | 8.216852 |
| TCGA-DA-A1I2 | 14.71233 | 1 | 9.088515 | 2.684892 | 4.664993 | 6.304881 | 7.436078 | 6.661502 | 3.548544 | 4.78 | 6.808567 | 5.593221 | 1.512419 | 5.690545 | 2.282472 | 0.629813 | 8.865828 |
| TCGA-ER-A195 | 2.953425 | 1 | 7.513933 | 4.358096 | 6.278997 | 7.16174 | 5.707547 | 7.584319 | 5.594292 | 3.844109 | 7.15767 | 5.708662 | 2.636493 | 2.862264 | 3.914651 | 1.128141 | 9.103504 |
| TCGA-D3-A3BZ | 10.89315 | 0 | 6.546306 | 3.420472 | 6.788251 | 6.065015 | 5.290607 | 6.092531 | 4.527956 | 2.996812 | 7.349502 | 6.121249 | 1.935023 | 3.165147 | 5.081679 | 1.141914 | 10.49427 |
| TCGA-XV-AAZW | 1.076712 | 1 | 6.241663 | 1.267728 | 2.588931 | 4.754656 | 2.879549 | 2.385714 | 1.205581 | 2.7912 | 8.694418 | 5.57988 | 1.817974 | 4.024213 | 0.491843 | 0.714973 | 9.651786 |
| TCGA-D3-A2J6 | 3.619178 | 1 | 9.3829 | 2.448709 | 6.000929 | 4.721791 | 4.509791 | 4.235674 | 2.317176 | 3.532755 | 7.608113 | 6.855134 | 1.36328 | 4.402595 | 1.737038 | 1.940469 | 7.349502 |
| TCGA-BF-A3DN | 1.964384 | 0 | 6.99958 | 1.930978 | 3.080171 | 5.329508 | 2.429518 | 2.304835 | 2.012469 | 3.183551 | 8.049629 | 6.851959 | 0.74644 | 3.863381 | 2.624032 | 0.43076 | 9.48095 |
| TCGA-WE-AAA0 | 3.367123 | 0 | 9.463852 | 2.40778 | 4.125788 | 4.771476 | 4.721791 | 3.710732 | 3.134122 | 3.709693 | 8.70773 | 5.09796 | 1.483919 | 4.737662 | 4.086252 | 0.817873 | 9.166141 |
| TCGA-XV-AAZY | 1.109589 | 0 | 8.496625 | 2.66414 | 5.776347 | 5.361799 | 2.779742 | 3.61259 | 2.578884 | 2.949907 | 8.359438 | 4.810162 | 1.433552 | 3.893662 | 4.320525 | 0.359572 | 9.651786 |
| TCGA-WE-A8K1 | 4.087671 | 0 | 10.67807 | 2.841096 | 7.137556 | 5.22129 | 6.889013 | 6.678986 | 3.557445 | 3.690174 | 8.289803 | 5.796755 | 2.533454 | 4.298877 | 3.260307 | 0.66456 | 7.295768 |
| TCGA-ER-A3ET | 7.750685 | 1 | 8.950546 | 4.670343 | 3.622355 | 8.334715 | 4.834045 | 3.701397 | 5.809374 | 3.831168 | 7.16174 | 7.340424 | 2.197081 | 4.224218 | 2.886171 | 1.016339 | 10.30748 |
| TCGA-FS-A1ZT | 4.430137 | 0 | 6.234527 | 3.545787 | 5.566737 | 5.657501 | 6.040997 | 6.691635 | 4.841666 | 4.267048 | 6.476693 | 6.18826 | 2.268334 | 2.607102 | 4.329335 | 0.747626 | 7.340424 |
| TCGA-D3-A51T | 2.241096 | 0 | 8.25787 | 2.688276 | 5.171486 | 4.495624 | 4.887011 | 5.545684 | 2.730242 | 2.704453 | 8.041226 | 5.936317 | 1.735896 | 4.844586 | 3.076744 | 0.306947 | 9.651786 |
| TCGA-EE-A29T | 30.8274 | 0 | 8.445874 | 4.601902 | 6.259385 | 6.634665 | 4.257685 | 4.627563 | 5.373023 | 3.508164 | 6.72588 | 7.251809 | 3.101634 | 4.041793 | 3.41173 | 1.190527 | 10.12502 |
| TCGA-ER-A3EV | 3.915068 | 1 | 6.336661 | 3.123109 | 5.62275 | 4.587197 | 3.340738 | 3.370926 | 2.924289 | 3.134122 | 7.503515 | 5.649767 | 0.853592 | 2.463821 | 1.585189 | 0.700171 | 8.058297 |
| TCGA-XV-AB01 | 1.10411 | 0 | 7.114687 | 3.278214 | 5.400459 | 5.735031 | 2.653084 | 3.653244 | 2.615591 | 1.852261 | 7.466493 | 5.948588 | 1.503668 | 3.887949 | 3.264498 | 0.588366 | 9.947093 |
| TCGA-WE-A8K6 | 1.49589 | 0 | 5.745558 | 3.044881 | 3.208277 | 7.503515 | 3.50766 | 1.342188 | 3.005582 | 3.203125 | 7.300321 | 5.423995 | 1.790551 | 3.67101 | 0.61414 | 0.88124 | 12.64537 |
| TCGA-D3-A8GJ | 20.11507 | 0 | 9.688332 | 3.836286 | 8.907748 | 7.363574 | 9.320966 | 8.773145 | 3.988871 | 3.344026 | 7.546676 | 6.526134 | 3.614739 | 5.508969 | 3.10473 | 0.810021 | 10.04373 |
| TCGA-QB-A6FS | 0.60274 | 0 | 8.268444 | 3.845553 | 6.755326 | 6.21546 | 3.799677 | 4.333344 | 3.532084 | 2.146119 | 8.509861 | 6.87963 | 2.47121 | 3.537224 | 4.595878 | 1.204837 | 9.180879 |
| TCGA-GN-A266 | 0.843836 | 1 | 7.578904 | 4.918986 | 6.13737 | 8.236801 | 7.853397 | 7.363574 | 6.539507 | 4.129059 | 6.002421 | 6.316095 | 3.092827 | 2.860691 | 2.5599 | 1.471065 | 10.27357 |
| TCGA-RP-A693 | 0.027397 | 0 | 9.398771 | 3.958889 | 5.790685 | 7.349502 | 6.163384 | 6.394526 | 5.013829 | 3.846675 | 7.431397 | 5.884054 | 2.740024 | 4.151995 | 2.998366 | 1.29587 | 9.010101 |
| TCGA-ER-A19L | 10.9589 | 1 | 5.162018 | 3.230161 | 2.643286 | 5.661771 | 1.787325 | 1.496697 | 3.04898 | 4.290962 | 6.264617 | 5.587088 | 0.85733 | 3.894037 | 2.296043 | 0.606318 | 9.581285 |
| TCGA-D3-A2JB | 14 | 1 | 7.492978 | 2.786837 | 6.731229 | 6.39247 | 5.643166 | 6.386552 | 4.738727 | 3.544748 | 8.89291 | 6.059103 | 3.401277 | 4.071725 | 6.649415 | 0.795989 | 7.974782 |
| TCGA-EE-A29E | 5.315068 | 0 | 2.770804 | 1.798496 | 1.85315 | 3.956952 | 1.187873 | 1.54986 | 1.385467 | 2.92143 | 7.831019 | 5.983812 | 0.386368 | 3.829243 | 1.338973 | 1.488546 | 9.258211 |
| TCGA-D3-A51E | 14.56986 | 0 | 8.852298 | 3.577141 | 6.16013 | 5.998214 | 7.625453 | 7.631178 | 4.829938 | 3.462781 | 7.83865 | 7.006306 | 2.555408 | 4.570606 | 1.902193 | 1.108549 | 7.48764 |
| TCGA-FS-A1ZE | 3.871233 | 1 | 2.773209 | 1.835278 | 2.14729 | 3.227268 | 1.894528 | 3.113949 | 2.034517 | 2.826201 | 6.749759 | 4.792996 | 0.58607 | 4.328005 | 0.8657 | 0.497003 | 7.882242 |
| TCGA-FS-A4F0 | 6.484932 | 0 | 10.62899 | 1.109331 | 1.828729 | 2.131075 | 2.193193 | 1.558454 | 0.652866 | 5.597486 | 9.041563 | 5.969603 | 0.170335 | 6.470104 | 1.91533 | 0.56052 | 9.463852 |
| TCGA-WE-A8ZX | 2.983562 | 0 | 8.549656 | 2.956432 | 6.317951 | 5.868327 | 6.23283 | 6.720752 | 3.8207 | 3.311222 | 7.095916 | 6.264617 | 2.155984 | 2.648405 | 2.960157 | 0.788166 | 9.651786 |
| TCGA-FR-A2OS | 1.008219 | 1 | 5.821676 | 2.152646 | 1.912915 | 5.433148 | 0.765291 | 0.494435 | 2.918325 | 2.958307 | 6.958602 | 4.976377 | 0.72762 | 3.869708 | 0.690931 | 0.678385 | 9.088515 |
| TCGA-D9-A1X3 | 1.509589 | 0 | 2.852751 | 2.010976 | 2.031211 | 3.729065 | 0.476439 | 0.367426 | 1.576308 | 3.285395 | 8.630449 | 4.586687 | 0.536734 | 4.503425 | 1.609702 | 0.228991 | 7.882242 |
| TCGA-WE-A8ZN | 4.915068 | 0 | 8.408325 | 3.236682 | 7.349502 | 6.720752 | 4.933278 | 5.515665 | 3.112668 | 2.757516 | 9.564239 | 4.806751 | 2.628628 | 3.850337 | 2.833654 | 1.413896 | 9.547666 |
| TCGA-EB-A57M | 1.293151 | 1 | 5.562685 | 3.001542 | 4.203338 | 5.905183 | 2.893239 | 2.287034 | 3.139319 | 2.61799 | 7.226721 | 5.744404 | 1.788456 | 4.400788 | 2.77635 | 1.107682 | 7.769483 |
| TCGA-EE-A2MI | 17.05479 | 1 | 7.562478 | 4.709836 | 5.341168 | 8.445874 | 5.356568 | 6.526134 | 5.834206 | 3.846276 | 5.764414 | 5.191714 | 3.138316 | 3.265494 | 2.018443 | 1.681475 | 10.41592 |
| TCGA-WE-AA9Y | 1.013699 | 0 | 5.660635 | 3.157175 | 4.175824 | 6.180146 | 0.779923 | 0.943222 | 3.471565 | 2.32252 | 6.271724 | 3.357041 | 1.80677 | 3.590006 | 4.378591 | 0.849358 | 9.367006 |
| TCGA-D9-A1JX | 0.591781 | 1 | 9.274058 | 3.749969 | 6.259385 | 6.699641 | 5.418532 | 5.849268 | 4.851699 | 3.2237 | 7.308973 | 6.892185 | 2.267135 | 3.79199 | 3.094093 | 0.932387 | 8.226828 |
| TCGA-ER-A19A | 6.479452 | 0 | 7.692498 | 6.180146 | 8.138531 | 9.320966 | 8.799477 | 9.581285 | 7.673388 | 3.868199 | 7.411642 | 7.546676 | 4.129481 | 3.95657 | 4.973876 | 2.360392 | 9.48095 |
| TCGA-ER-A42K | 1.079452 | 1 | 7.625453 | 2.25027 | 4.290962 | 5.007785 | 2.36672 | 2.525871 | 2.930525 | 3.779899 | 5.799272 | 6.982079 | 0.816005 | 2.434681 | 4.15533 | 0.877045 | 12.12313 |
| TCGA-D3-A5GR | 14.86027 | 0 | 7.711737 | 2.92519 | 5.392477 | 5.853116 | 5.541686 | 7.21417 | 5.092322 | 3.510863 | 5.764414 | 7.062695 | 3.124396 | 3.083032 | 5.404973 | 0.85655 | 10.41592 |
| TCGA-WE-A8ZQ | 5.268493 | 0 | 7.590229 | 1.696116 | 0.919721 | 4.986696 | 0.689086 | 0.942393 | 1.908937 | 2.423509 | 5.815556 | 6.091038 | 0.363645 | 2.691047 | 1.362683 | 0.518714 | 9.196354 |
| TCGA-EE-A2MG | 8.6 | 1 | 8.158476 | 3.475715 | 6.078894 | 6.185003 | 5.340305 | 6.487264 | 5.404089 | 3.766004 | 6.199999 | 6.150391 | 3.148925 | 4.469953 | 7.295768 | 0.582286 | 9.799361 |
| TCGA-QB-AA9O | 1.50411 | 1 | 5.321875 | 3.152364 | 6.870341 | 6.510921 | 4.899716 | 5.917329 | 3.529688 | 2.660764 | 8.058297 | 4.738727 | 2.871181 | 4.255091 | 1.808806 | 0.582137 | 9.616823 |
| TCGA-ER-A194 | 3.709589 | 1 | 6.082023 | 3.059914 | 6.758168 | 5.654199 | 3.825171 | 4.527444 | 2.652639 | 2.394954 | 8.562525 | 4.809006 | 1.875237 | 4.424117 | 2.871487 | 0.49925 | 9.76173 |
| TCGA-ER-A19S | 4.123288 | 0 | 9.057216 | 4.634257 | 8.589809 | 7.451022 | 7.93477 | 9.398771 | 5.969603 | 3.824783 | 6.526134 | 7.099723 | 3.91542 | 4.295766 | 3.799677 | 1.563786 | 9.088515 |
| TCGA-D3-A3ML | 1.156164 | 1 | 7.867894 | 1.314701 | 1.763887 | 2.111906 | 1.781664 | 1.435145 | 0.791886 | 3.897905 | 5.178268 | 4.943333 | 0.34932 | 4.493248 | 1.219015 | 0.778233 | 7.304577 |
| TCGA-FS-A1ZB | 4.071233 | 1 | 8.25787 | 4.809578 | 4.262793 | 7.141812 | 3.662409 | 3.91163 | 4.883424 | 3.853725 | 8.226828 | 5.553772 | 3.051529 | 3.333868 | 2.986005 | 1.264482 | 9.564239 |
| TCGA-EE-A2MM | 13.99178 | 1 | 9.838554 | 3.157822 | 3.389878 | 6.014162 | 5.948588 | 3.535904 | 3.93726 | 5.200661 | 8.268444 | 6.840305 | 1.139916 | 4.340157 | 1.570371 | 1.22177 | 7.584319 |
| TCGA-EE-A3JE | 4.279452 | 0 | 8.825986 | 5.14018 | 8.879077 | 6.441018 | 9.513137 | 7.705489 | 5.671445 | 3.572659 | 7.999387 | 6.802687 | 3.865619 | 5.522496 | 4.721242 | 1.269617 | 9.336361 |
| TCGA-GF-A4EO | 1.619178 | 0 | 8.187025 | 4.8022 | 7.782923 | 7.743395 | 7.451022 | 8.99563 | 6.13737 | 3.251216 | 6.534983 | 7.313439 | 4.614148 | 3.95657 | 4.174136 | 1.480941 | 8.70773 |
| TCGA-FS-A4F9 | 2.835616 | 0 | 4.385929 | 1.455336 | 2.056502 | 3.614739 | 1.912002 | 3.097247 | 1.543139 | 2.881736 | 6.948914 | 6.264617 | 0.713629 | 2.517703 | 1.082995 | 0.310594 | 7.201831 |
| TCGA-WE-A8ZY | 4.126027 | 1 | 10.18136 | 2.374873 | 3.043313 | 4.773117 | 2.919572 | 2.622526 | 2.217528 | 4.851699 | 7.776094 | 6.728404 | 0.542188 | 4.783957 | 2.584056 | 0.377548 | 8.128757 |
| TCGA-FR-A44A | 14.51781 | 0 | 8.346721 | 5.307732 | 9.211383 | 7.373601 | 9.088515 | 10.01891 | 6.217105 | 3.618256 | 5.484554 | 7.295768 | 4.16742 | 5.189458 | 3.863381 | 1.799424 | 9.497298 |
| TCGA-W3-AA1R | 9.257534 | 1 | 7.243384 | 2.029189 | 5.123399 | 4.611623 | 4.781178 | 4.504424 | 2.101634 | 2.99187 | 7.743395 | 6.029007 | 2.067305 | 3.914275 | 1.663221 | 0.368591 | 7.78969 |
| TCGA-EB-A299 | 1.035616 | 0 | 6.312323 | 1.701664 | 4.756384 | 6.254056 | 5.057571 | 5.006465 | 2.129578 | 2.779123 | 6.656557 | 5.238407 | 4.417182 | 4.154071 | 1.364265 | 1.133071 | 10.2121 |
| TCGA-EE-A2ML | 18.05479 | 1 | 8.825986 | 3.259 | 5.503072 | 6.62527 | 4.859854 | 5.008452 | 4.149929 | 3.337804 | 7.239183 | 8.359438 | 2.14488 | 4.684509 | 3.797115 | 1.05696 | 9.547666 |
| TCGA-YD-A9TA | 4.09863 | 0 | 6.587577 | 3.673846 | 5.418532 | 6.718114 | 5.112564 | 5.98679 | 4.607519 | 3.50315 | 5.745558 | 5.206002 | 2.50083 | 1.572108 | 2.004068 | 0.882344 | 9.799361 |
| TCGA-D3-A1Q3 | 1.389041 | 1 | 6.873441 | 2.753219 | 4.583106 | 5.238407 | 3.731189 | 6.400558 | 3.985358 | 3.622542 | 8.922381 | 3.057153 | 2.381779 | 4.564156 | 4.97765 | 1.104586 | 8.786252 |
| TCGA-D9-A1JW | 0.30411 | 1 | 8.879077 | 4.000558 | 6.546306 | 5.679336 | 5.04325 | 6.278997 | 4.569619 | 3.092827 | 7.643215 | 5.386298 | 2.619234 | 4.826453 | 4.646611 | 1.457003 | 7.782923 |
| TCGA-EE-A2GC | 5.619178 | 0 | 8.359438 | 4.381305 | 6.428485 | 6.60147 | 8.865828 | 7.243384 | 5.023065 | 3.570208 | 6.892185 | 5.811817 | 3.342409 | 4.246922 | 2.621281 | 1.156538 | 8.216852 |
| TCGA-FR-A7U8 | 2.320548 | 0 | 9.497298 | 2.591239 | 2.126632 | 4.65917 | 2.340858 | 2.072073 | 1.868929 | 4.06525 | 7.584319 | 5.783432 | 0.678796 | 4.091098 | 1.131361 | 0.416142 | 8.312242 |
| TCGA-EE-A29A | 5.279452 | 1 | 7.382795 | 2.232552 | 1.923087 | 4.578055 | 1.30469 | 1.569805 | 2.356478 | 4.314319 | 5.701828 | 1.898923 | 0.593222 | 2.848422 | 1.223048 | 2.110646 | 9.859751 |
| TCGA-W3-AA1W | 18.26301 | 0 | 9.669984 | 4.622924 | 9.463852 | 8.058297 | 8.236801 | 9.3829 | 5.98679 | 4.25509 | 7.239183 | 7.698557 | 3.687291 | 5.653083 | 5.167093 | 1.88888 | 9.166141 |
| TCGA-FS-A1ZA | 2.309589 | 1 | 4.677684 | 2.740024 | 4.301946 | 5.265428 | 4.821179 | 6.323705 | 4.030144 | 3.56092 | 6.651794 | 3.083978 | 1.744929 | 2.670423 | 2.123202 | 1.099779 | 10.27357 |
| TCGA-EB-A44N | 0.561644 | 1 | 7.503515 | 1.703734 | 4.619336 | 5.007785 | 5.199216 | 5.072697 | 2.665334 | 2.965105 | 7.39195 | 5.929582 | 4.597365 | 3.41303 | 3.513216 | 1.416102 | 7.999387 |
| TCGA-FS-A1Z4 | 2.339726 | 1 | 8.075346 | 3.758688 | 6.130924 | 5.821676 | 6.293738 | 6.317951 | 5.074088 | 4.545305 | 5.269422 | 6.231112 | 2.750789 | 4.162484 | 6.340385 | 1.085294 | 9.547666 |
| TCGA-D9-A6EA | 2.09863 | 0 | 8.839039 | 2.393158 | 3.594823 | 4.885795 | 3.933082 | 2.878328 | 2.382403 | 3.902104 | 8.458672 | 5.57988 | 0.600813 | 4.035742 | 1.033552 | 0.84299 | 9.274058 |
| TCGA-EE-A3JI | 12.73425 | 1 | 8.99563 | 3.40407 | 3.071772 | 6.736535 | 4.495149 | 2.022061 | 3.345311 | 5.539643 | 6.534983 | 6.466031 | 1.557872 | 4.85405 | 3.011496 | 0.520976 | 7.264612 |
| TCGA-ER-A199 | 0.764384 | 1 | 8.562525 | 3.858157 | 6.510921 | 6.495597 | 4.988005 | 5.524526 | 5.210583 | 2.928941 | 6.916855 | 7.824028 | 1.998625 | 3.918115 | 6.380644 | 1.250655 | 9.634608 |
| TCGA-DA-A1IC | 5.673973 | 1 | 6.676534 | 2.3519 | 4.35629 | 4.556812 | 3.09381 | 4.404437 | 2.535863 | 3.386928 | 6.892185 | 5.329508 | 1.745515 | 3.01936 | 2.015214 | 0.840333 | 8.206697 |
| TCGA-D3-A5GN | 11.31233 | 0 | 8.289803 | 2.620356 | 5.666139 | 4.962358 | 6.363285 | 6.948914 | 3.462781 | 3.233404 | 6.707448 | 6.478848 | 1.815035 | 3.494908 | 2.323708 | 0.993766 | 9.651786 |
| TCGA-3N-A9WB | 1.419178 | 1 | 7.149764 | 1.456213 | 1.975351 | 3.01745 | 1.089476 | 0.76108 | 0.632014 | 3.213193 | 9.448603 | 4.560256 | 0.828556 | 4.664993 | 0.888775 | 0.838979 | 8.101991 |
| TCGA-EB-A553 | 0.619178 | 0 | 6.851959 | 2.126313 | 5.795564 | 4.757495 | 2.730517 | 5.528397 | 1.975622 | 2.983489 | 9.15014 | 4.668765 | 1.991747 | 2.870886 | 2.81139 | 0.166934 | 10.58227 |
| TCGA-EB-A5UL | 2.441096 | 0 | 7.062695 | 1.948216 | 6.206777 | 4.423677 | 5.146759 | 6.210251 | 2.562021 | 3.549904 | 7.649042 | 5.85953 | 2.031532 | 3.328562 | 3.592062 | 0.87125 | 8.617038 |
| TCGA-FS-A4F4 | 5.556164 | 1 | 7.03403 | 3.364629 | 5.898357 | 6.178403 | 3.125022 | 3.863381 | 2.965399 | 2.489555 | 8.922381 | 5.111129 | 1.995372 | 4.283483 | 2.632563 | 0.398989 | 9.780437 |
| TCGA-EB-A3XB | 2.180822 | 0 | 6.758168 | 3.042973 | 4.584118 | 5.63658 | 2.933328 | 3.220193 | 3.15655 | 2.759366 | 8.408325 | 6.651794 | 1.790816 | 3.973374 | 2.018443 | 0.437998 | 8.81294 |
| TCGA-DA-A95X | 6.161644 | 0 | 7.596001 | 3.918524 | 4.422718 | 6.176738 | 5.124828 | 6.254056 | 4.791302 | 4.055631 | 5.505052 | 7.919211 | 1.947356 | 4.282157 | 1.447775 | 1.127242 | 9.211383 |
| TCGA-D3-A1Q8 | 2.339726 | 1 | 8.041226 | 4.511262 | 3.307373 | 8.279393 | 2.364908 | 2.223805 | 5.167803 | 3.295777 | 7.239183 | 6.576047 | 2.129312 | 1.7897 | 3.635582 | 0.799425 | 10.78293 |
| TCGA-HR-A2OH | 5.490411 | 1 | 9.724841 | 5.184257 | 8.746058 | 7.853397 | 8.980385 | 9.497298 | 6.739135 | 3.221477 | 7.769483 | 7.705489 | 3.51287 | 5.414836 | 4.803905 | 1.902499 | 9.398771 |
| TCGA-D9-A3Z1 | 1.282192 | 1 | 6.189925 | 4.415836 | 6.530735 | 6.404654 | 5.760813 | 5.920111 | 5.129173 | 3.540303 | 7.803093 | 6.048457 | 3.488868 | 3.211878 | 3.21513 | 1.598886 | 6.526134 |
| TCGA-D9-A4Z6 | 1.536986 | 1 | 5.646451 | 1.225899 | 2.737292 | 3.377537 | 1.996706 | 1.59116 | 1.214527 | 4.150773 | 7.114687 | 5.214405 | 0.280633 | 2.792717 | 0.317203 | 0.816553 | 8.471285 |
| TCGA-EE-A29S | 5.106849 | 1 | 8.066661 | 3.087146 | 5.203658 | 5.309322 | 3.647542 | 4.809006 | 3.651852 | 3.228544 | 6.771667 | 7.264612 | 1.613398 | 3.892887 | 2.745571 | 0.877249 | 10.15165 |
| TCGA-EB-A82B | 1.068493 | 0 | 8.825986 | 3.233718 | 5.902494 | 6.59212 | 5.274982 | 5.015794 | 3.484185 | 3.500739 | 7.471659 | 6.828652 | 1.906325 | 4.173724 | 2.075054 | 0.819088 | 7.882242 |
| TCGA-EB-A85J | 0.986301 | 0 | 7.492978 | 3.120891 | 7.273262 | 6.573981 | 7.451022 | 5.58909 | 4.410808 | 2.658386 | 7.874921 | 6.132505 | 2.376691 | 3.953878 | 3.418083 | 0.890505 | 7.277861 |
| TCGA-D3-A2JL | 14.29863 | 0 | 8.395557 | 3.054302 | 7.803093 | 6.206777 | 5.294618 | 6.699641 | 3.333191 | 3.889083 | 7.222808 | 6.161723 | 3.399765 | 5.054897 | 3.616474 | 0.843187 | 8.907748 |
| TCGA-D3-A3MV | 3.775342 | 0 | 7.982479 | 2.859133 | 2.342412 | 5.941667 | 2.421375 | 0.633047 | 3.192489 | 3.804463 | 6.794023 | 6.135805 | 0.82716 | 2.306051 | 1.681475 | 0.362341 | 8.879077 |
| TCGA-ER-A19B | 8.2 | 1 | 2.692898 | 2.000455 | 3.314396 | 4.728934 | 1.489967 | 2.101305 | 2.228351 | 3.533102 | 6.487264 | 8.268444 | 0.624201 | 2.880163 | 4.394719 | 0.51326 | 9.448603 |
| TCGA-EE-A3AG | 3.465753 | 1 | 3.752834 | 1.800578 | 2.425011 | 4.745876 | 1.796143 | 1.280138 | 2.458088 | 2.783134 | 5.905183 | 5.863333 | 0.553711 | 1.236088 | 0.37545 | 0.242748 | 9.724841 |
| TCGA-EE-A2GR | 3.564384 | 1 | 3.056838 | 0.959668 | 1.588329 | 2.061312 | 2.31145 | 2.489247 | 1.048412 | 3.150795 | 8.509861 | 3.993594 | 0.188671 | 3.742744 | 0.381217 | 0.399099 | 7.461545 |
| TCGA-D3-A2JO | 5.506849 | 0 | 8.289803 | 4.514692 | 8.049629 | 7.048227 | 6.594455 | 7.737162 | 5.747916 | 3.629293 | 6.457635 | 6.138973 | 3.162323 | 2.392855 | 3.248968 | 1.650672 | 9.616823 |
| TCGA-BF-AAOU | 1.30411 | 0 | 8.523571 | 2.577983 | 3.869333 | 5.979426 | 1.623894 | 2.01998 | 2.376691 | 2.840187 | 7.846293 | 5.490445 | 0.981502 | 4.966847 | 2.180304 | 0.740716 | 11.08967 |
| TCGA-DA-A3F8 | 3.613699 | 0 | 9.598808 | 4.73166 | 9.010101 | 7.476715 | 8.799477 | 9.305759 | 5.717757 | 3.695923 | 7.466493 | 7.79637 | 3.486876 | 5.481674 | 4.988676 | 1.728555 | 9.547666 |
| TCGA-FR-A728 | 1.59726 | 0 | 9.859751 | 3.874557 | 6.694161 | 6.224058 | 6.140573 | 4.927591 | 4.172462 | 2.814167 | 8.110368 | 7.625453 | 1.978034 | 4.579597 | 3.271062 | 1.120579 | 11.16651 |
| TCGA-EE-A3JA | 4.432877 | 1 | 5.472167 | 4.208439 | 5.924147 | 4.420403 | 6.206777 | 6.125933 | 5.231342 | 3.266466 | 6.196628 | 5.568744 | 3.170866 | 5.347142 | 3.026274 | 0.527237 | 7.16174 |
| TCGA-FS-A1YW | 18.07671 | 1 | 4.652333 | 2.367634 | 3.1162 | 4.445584 | 2.770479 | 3.119302 | 2.971296 | 3.181994 | 4.861033 | 6.191603 | 1.100676 | 3.373526 | 2.991265 | 0.516104 | 7.340424 |
| TCGA-FS-A1ZG | 0.808219 | 1 | 4.810162 | 2.566829 | 2.860083 | 4.124963 | 2.179112 | 2.416555 | 2.509194 | 3.73013 | 9.43193 | 4.125381 | 0.683189 | 2.740947 | 1.534337 | 0.921265 | 5.609125 |
| TCGA-EE-A2M6 | 10.7726 | 0 | 5.370323 | 3.822158 | 3.563679 | 7.173327 | 4.332862 | 3.706775 | 5.127009 | 3.005266 | 6.314155 | 7.831019 | 2.292421 | 1.292807 | 1.659731 | 0.866281 | 7.991229 |
| TCGA-DA-A1HV | 6.380822 | 0 | 9.367006 | 4.056013 | 6.779902 | 6.867298 | 7.125979 | 7.897175 | 5.270985 | 3.71003 | 6.608574 | 6.557743 | 3.493594 | 4.436261 | 3.476386 | 0.975279 | 9.598808 |
| TCGA-D3-A5GU | 10.43288 | 0 | 8.458672 | 3.489528 | 5.772846 | 6.487264 | 5.66932 | 5.897063 | 3.843743 | 3.425778 | 7.608113 | 5.188749 | 2.265626 | 5.167803 | 2.481303 | 0.442006 | 8.549656 |
| TCGA-D9-A6EG | 1.912329 | 1 | 4.359881 | 2.086857 | 1.787048 | 4.403515 | 1.006012 | 0.882803 | 1.823072 | 3.215774 | 7.966601 | 5.162018 | 0.680531 | 2.57737 | 1.018946 | 0.451155 | 5.969603 |
| TCGA-BF-A5EO | 1.926027 | 0 | 8.247378 | 2.729946 | 5.044575 | 6.026011 | 3.340431 | 3.927005 | 2.559026 | 2.452633 | 8.965263 | 6.282684 | 0.962751 | 3.602165 | 1.816788 | 0.641759 | 10.34278 |
| TCGA-EB-A6R0 | 1.665753 | 1 | 10.27357 | 2.735133 | 6.972049 | 4.542402 | 9.879919 | 6.50657 | 3.647177 | 4.9041 | 7.982479 | 6.072573 | 1.281722 | 6.398654 | 2.397692 | 0.503958 | 7.557357 |
| TCGA-Z2-A8RT | 2.29863 | 0 | 8.773145 | 3.194721 | 5.420319 | 5.775164 | 5.682712 | 6.351632 | 4.237891 | 4.071322 | 6.873441 | 6.011146 | 1.949709 | 5.116166 | 3.499719 | 0.997007 | 9.025559 |
| TCGA-EB-A6L9 | 3.038356 | 0 | 8.049629 | 3.011804 | 6.357439 | 5.515665 | 5.092322 | 5.698423 | 4.088651 | 2.83427 | 6.459664 | 5.633394 | 2.562918 | 4.406265 | 6.661502 | 1.105623 | 8.603012 |
| TCGA-ER-A2NG | 4.082192 | 1 | 7.535743 | 4.301509 | 8.825986 | 6.557743 | 7.129786 | 7.551771 | 5.799272 | 3.818108 | 7.205721 | 7.666866 | 2.658968 | 4.28656 | 3.152047 | 1.782231 | 9.742234 |
| TCGA-FS-A1YX | 4.049315 | 1 | 6.478848 | 3.164839 | 3.259 | 6.416277 | 4.33604 | 2.582201 | 3.616474 | 3.600417 | 7.608113 | 7.044609 | 1.056005 | 3.290567 | 1.892517 | 0.750831 | 10.89923 |
| TCGA-D3-A8GD | 1.967123 | 0 | 9.180879 | 3.92275 | 8.279393 | 6.733851 | 6.739135 | 8.433625 | 5.402294 | 4.176259 | 6.649415 | 5.905183 | 4.379495 | 5.572789 | 5.738534 | 1.042747 | 9.3829 |
| TCGA-ER-A19E | 1.084932 | 1 | 5.339459 | 2.706883 | 3.990072 | 5.647627 | 3.475381 | 4.216545 | 3.500397 | 3.344343 | 5.046524 | 5.015794 | 1.362683 | 1.038377 | 1.475331 | 0.650005 | 9.274058 |
| TCGA-EB-A3HV | 0.106849 | 0 | 7.81695 | 3.486876 | 3.938824 | 5.988206 | 0.784268 | 1.333667 | 3.10005 | 2.629513 | 6.98562 | 5.583976 | 3.344343 | 4.120457 | 1.884706 | 1.749692 | 10.41592 |
| TCGA-EB-A5SE | 1.09863 | 1 | 8.058297 | 1.37283 | 3.439615 | 3.654295 | 3.511191 | 3.193747 | 1.506581 | 3.105068 | 8.922381 | 5.64421 | 1.160472 | 3.351085 | 1.00017 | 0.567629 | 6.758168 |
| TCGA-FR-A8YD | 3.021918 | 1 | 6.119597 | 2.384497 | 3.766386 | 5.011195 | 1.469254 | 2.502632 | 2.536807 | 2.034517 | 7.551771 | 5.596475 | 1.146793 | 3.512189 | 1.503369 | 0.569449 | 8.075346 |
| TCGA-WE-A8K4 | 1.682192 | 0 | 9.901675 | 2.903169 | 8.101991 | 5.085245 | 6.689249 | 5.808119 | 3.066174 | 4.200413 | 7.643215 | 5.213665 | 2.269829 | 5.261451 | 2.435875 | 2.08985 | 6.774378 |
| TCGA-EE-A29L | 0.216438 | 1 | 6.892185 | 0.734883 | 1.557007 | 1.779834 | 1.428844 | 1.572389 | 0.599589 | 4.042928 | 8.312242 | 4.521544 | 0.159884 | 2.914343 | 1.194512 | 1.017053 | 6.16013 |
| TCGA-FS-A4F5 | 2.394521 | 1 | 7.426424 | 2.028566 | 2.528023 | 4.419934 | 5.226694 | 5.073416 | 2.386 | 3.028717 | 7.803093 | 6.225822 | 0.854251 | 4.587197 | 1.373644 | 0.304886 | 9.241958 |
