# Supplementary material for: A novel inflammatory response-related signature predicts the prognosis of cutaneous melanoma and the effect of antitumor drugs
Source: World J Surg Oncol. 2022 Aug 19;20:263. doi: 10.1186/s12957-022-02726-8 (PMC9389732; doi:10.1186/s12957-022-02726-8)
Supplement: Supplementary file 4 — Additional file 4. [file 12957_2022_2726_MOESM4_ESM.docx]

| gene | HR | HR.95L | HR.95H | pvalue |
| --- | --- | --- | --- | --- |
| BST2 | 0.863827 | 0.80764 | 0.923924 | 1.99E-05 |
| C3AR1 | 0.791781 | 0.705239 | 0.888942 | 7.71E-05 |
| CCL5 | 0.860815 | 0.806536 | 0.918748 | 6.48E-06 |
| CD14 | 0.844397 | 0.77306 | 0.922318 | 0.000173 |
| CXCL10 | 0.851113 | 0.802131 | 0.903085 | 9.78E-08 |
| CXCL9 | 0.870617 | 0.824397 | 0.919429 | 6.42E-07 |
| CYBB | 0.819068 | 0.750814 | 0.893526 | 6.93E-06 |
| EIF2AK2 | 0.713835 | 0.567128 | 0.898491 | 0.004081 |
| EMP3 | 1.275461 | 1.114141 | 1.460139 | 0.000421 |
| ICAM1 | 0.842278 | 0.766017 | 0.926131 | 0.000393 |
| IL18 | 0.798134 | 0.706096 | 0.902168 | 0.00031 |
| RTP4 | 0.86353 | 0.774351 | 0.962979 | 0.008333 |
| SELL | 0.868222 | 0.793913 | 0.949487 | 0.001966 |
| SLC31A2 | 0.754304 | 0.571642 | 0.995333 | 0.046259 |
| TIMP1 | 0.85445 | 0.770694 | 0.947308 | 0.002805 |
